# Colorectal carcinoma peritoneal metastases-derived organoids: results and perspective of a model for tailoring hyperthermic intraperitoneal chemotherapy from bench-to-bedside

**DOI:** 10.1186/s13046-024-03052-5

**Published:** 2024-05-02

**Authors:** Luca Varinelli, Davide Battistessa, Marcello Guaglio, Susanna Zanutto, Oscar Illescas, Ewelina J. Lorenc, Federica Pisati, Shigeki Kusamura, Laura Cattaneo, Giovanna Sabella, Massimo Milione, Alessia Perbellini, Sara Noci, Cinzia Paolino, Elisabetta Kuhn, Margherita Galassi, Tommaso Cavalleri, Marcello Deraco, Manuela Gariboldi, Dario Baratti

**Affiliations:** 1 Department of Experimental Oncology, Molecular Epigenomics Unit, Fondazione IRCCS Istituto Nazionale Tumori, Via G. Venezian 1, Milan, 20133 Italy; 2 Peritoneal Surface Malignancies Unit, Colorectal Surgery, Fondazione IRCCS Istituto Nazionale Tumori, Via G. Venezian 1, Milan, 20133 Italy; 3 Cogentech Ltd. Benefit Corporation With a Sole Shareholder, Via Adamello 16, Milan, 20139 Italy; 4 Pathology and Laboratory Medicine Department, Fondazione IRCCS Istituto Nazionale Dei Tumori Di Milano, Via G. Venezian 1, Milan, 20133 Italy; 5 Department of Biomedical, Surgical and Dental Sciences, University of Milan, Milan, 20122 Italy; 6 Pathology Unit, Foundation IRCCS Ca’ Granda Ospedale Maggiore Policlinico, Milan, 20122 Italy; 7 Centrale Produzione Farmaci, Hospital Pharmacy, Fondazione IRCCS Istituto Nazionale Dei Tumori Di Milano, Via G. Venezian 1, Milan, 20133 Italy

**Keywords:** Peritoneal metastases, Colorectal cancer, Organoids, HIPEC, Tailored therapies, Chemotherapy, Personalized medicine

## Abstract

**Background:**

Peritoneal metastases from colorectal cancer (CRCPM) are related to poor prognosis. Cytoreductive surgery (CRS) and hyperthermic intraperitoneal chemotherapy (HIPEC) have been reported to improve survival, but peritoneal recurrence rates are still high and there is no consensus on the drug of choice for HIPEC. The aim of this study was to use patient derived organoids (PDO) to build a relevant CRCPM model to improve HIPEC efficacy in a comprehensive bench-to-bedside strategy.

**Methods:**

Oxaliplatin (L-OHP), cisplatin (CDDP), mitomycin-c (MMC) and doxorubicin (DOX) were used to mimic HIPEC on twelve PDO lines derived from twelve CRCPM patients, using clinically relevant concentrations. After chemotherapeutic interventions, cell viability was assessed with a luminescent assay, and the obtained dose–response curves were used to determine the half-maximal inhibitory concentrations. Also, induction of apoptosis by different HIPEC interventions on PDOs was studied by evaluating CASPASE3 cleavage.

**Results:**

Response to drug treatments varied considerably among PDOs. The two schemes with better response at clinically relevant concentrations included MMC alone or combined with CDDP. L-OHP showed relative efficacy only when administered at low concentrations over a long perfusion period. PDOs showed that the short course/high dose L-OHP scheme did not appear to be an effective choice for HIPEC in CRCPM. HIPEC administered under hyperthermia conditions enhanced the effect of chemotherapy drugs against cancer cells, affecting PDO viability and apoptosis. Finally, PDO co-cultured with cancer-associated fibroblast impacted HIPEC treatments by increasing PDO viability and reducing CASPASES activity.

**Conclusions:**

Our study suggests that PDOs could be a reliable in vitro model to evaluate HIPEC schemes at individual-patient level and to develop more effective treatment strategies for CRCPM.

**Supplementary Information:**

The online version contains supplementary material available at 10.1186/s13046-024-03052-5.

## Background

Colorectal cancer (CRC) is the third leading cause of cancer death in western countries. The peritoneum is the second site of metastatic spread of CRC after the liver, and about 25.000 new cases per year are expected in Western Countries [1]. Peritoneal metastases from colorectal cancer (CRCPM) are still associated with a worse prognosis and lower responsiveness to systemic chemotherapy (sCT) and targeted cancer drugs than the other CRC-derived metastases [2]. Cytoreductive surgery (CRS) combined with Hyperthermic intraperitoneal chemotherapy (HIPEC) is a curative-intent approach that has been shown to improve overall survival (OS) in selective retrospective cohorts [2].

The added value of HIPEC is still a matter of debate, and the European Society of Medical Oncology (ESMO) defines the procedure as merely investigational [3]. Moreover, the recent randomized trial Prodige-7, has failed to demonstrate a survival advantage related to oxaliplatin-based HIPEC in CRCPM patients undergoing optimal CRS (residual disease < 1 mm) [4]. Nevertheless, the clinical evidence that HIPEC can effectively target microscopic residual disease has been provided by three randomized trials for ovarian, gastric and, in the adjuvant setting, colorectal cancer [5]. These trials strongly suggest that efforts should be made to improve HIPEC efficacy in CRCPM rather than omitting HIPEC from treatment [6–8].

Oxaliplatin (OXL) efficacy issues have been pointed out as a possible reason for the failure of Prodigy-7 trial [9], but there is currently no consensus on the drug of choice for HIPEC. Mitomycin-C (MMC) alone or combined with cisplatin is largely used, but has never been directly tested against OXL, and retrospective studies have provided conflicting results [2]. We hypothesized that resistance to the drugs routinely used for HIPEC is related to the relatively high relapse rates still experienced after CRS/HIPEC, and that selecting the most active drug(s) at the individual-patient level can improve HIPEC efficacy. Patient-derived organoids (PDOs) are more specific and relevant human cancer models [10]. PDOs retain the genetic and phenotypic characteristics of the tumor of origin and more closely reflect the original cancer. PDOs derived from different tumor types have been shown to represent an in vitro surrogate for predicting therapeutic responses over a clinically actionable time frame [10–15]. Furthermore PDOs have been used to develop new therapeutic strategies to circumvent drug resistance [16]. Most importantly, concordance between molecular and metabolic features in PDOs and CRCPMs [10–17] provides an opportunity to study treatment response at the individual patient level.

We developed a comprehensive strategy involving CRCPM-derived PDOs and an *in-vitro* HIPEC model to select the most active drug among a set of agents suitable for intraperitoneal delivery.

## Experimental procedures

### Study design

This study complied with the Declaration of Helsinki and was approved by the Ethics Committee of the Fondazione IRCCS Istituto Nazionale dei Tumori of Milan, Italy (INT134/13; INT149/19; INT06/21). Written informed consent from each patient was obtained. The study included twelve patients with CRCPM selected according to the following criteria: i) pathologically confirmed CRCPM; ii) limited and surgically resectable peritoneal disease; iii) absence of distant metastases; iv) absence of severe morbidities contraindicating major surgery and v) signing of informed consent. Three representative CRCPM samples of 1 × 1 cm were collected for each patient. PDOs were developed, expanded and evaluated for concordance with the patient’s tissue following established protocols [18, 19]. PDOs were treated with five different HIPEC schemes. Different drug concentrations were analyzed to generate reproducible dose–response curves and determine IC_50_ values for each PDO line. The IC_50s_ were compared by assembling a heat-map consisting of the normalized Log plot of the median concentrations of all IC_50_ values obtained for each PDO line.

**Fig. 1:**
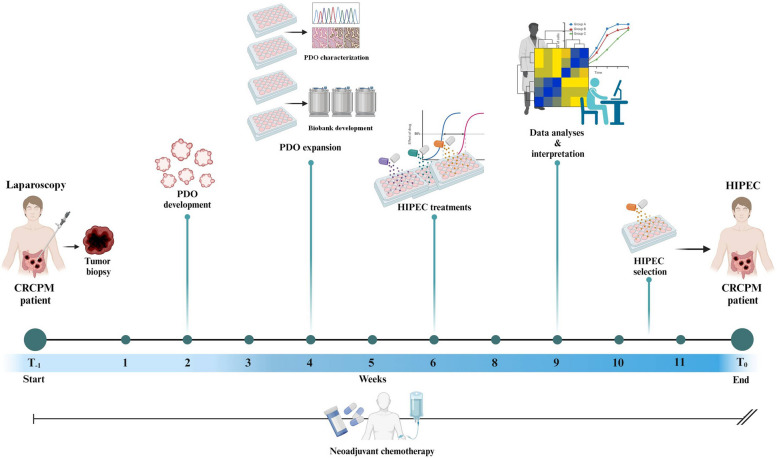
Study design. Flow chart representing the different phases of the HIPEC tailored treatment we have developed. *HIPEC* Hyperthermic IntraPEritoneal Chemotherapy, *CRCPM* Colorectal Cancer Peritoneal Metastasis, *PDO* Patient-derived Organoid

Tissue samples for PDO development were collected at different time points during patients’ history: at primary tumor resection (three patients with synchronous PM), at repeat surgery (one patient with recurrent PM), at CRS/HIPEC (three patients), and during laparoscopic procedures performed to confirm the presence of CRCPM and stage the disease (five patients). These five patients were included in a prospective phase II clinical study (Clinicaltrials.gov # NCT06057298) assessing the efficacy in controlling peritoneal disease of CRS with individual patient-tailored HIPEC, based on drug sensitivity tests performed in an in vitro HIPEC model on CRC-PM-derived organoids. After laparoscopy, patients receive 3–6 month preoperative s-CT with targeted agents, according to current guidelines. CRS and HIPEC techniques were described elsewhere [1–3]. Then, patients undergo clinical-radiological follow-up to record the occurrence of peritoneal recurrences, systemic (extra-peritoneal) metastases, and delayed treatment-related toxicities. A summary of the protocol is reported in Fig. 1.

### Human tissue collection

The specimens were placed in physiological solution, supplemented with 50 ng/ml gentamicin and 50 ng/ml amphotericin B and immediately transferred to the laboratory for developing PDOs and cancer-associated fibroblasts (CAFs). A part of the specimen was frozen in liquid nitrogen for molecular and histopathological analyses. Clinical information and characteristics of the patients enrolled in this study were reported in Table 1. All surgical procedures were done at Fondazione IRCCS Istituto Nazionale dei Tumori of Milan, from the Peritoneal Surface Malignancies Unit, Colorectal Surgery.

**Table 1:** Main pathological and clinical characteristics of the patients from whom PDOs were obtained

PDO line	Gender	Age	Site	Diagnosis	Stage	Grade	Mutations	Interval (months)	Previous CT	Tissue sampling for PDO development	PDO treatment	Patient treatment	Survival (months)	Recurrence site
C1	F	54	Left	Moderately differentiated infiltrating adenocarcinoma	pT3 N1b M1c	G2	*KRAS;* *TP53*	Synchronous	None	Primary surgery + CRS	MMC	ND	^c^17	-
C2	M	72	Right	Poorly differentiated mucinous multiple intestinal adenocarcinoma	pT4a N2b M1c	G3	*BRAF; TP53*	Synchronous	None	Primary surgery + CRS	MMC + CDDP	ND	^c^6	-
C3	F	58	Right	Intestinal mucinous adenocarcinoma	pT4b N2b M1c	G3	*KRAS; TP53;* *FGFR1* amplification	Synchronous	Folfox; Folfiri + Bevacizumab; Bevacizumab (maint)	CRS for recurrent PM	MMC	ND	^c^48	-
C4	F	71	Right	Intestinal adenocarcinoma	pT4a N2b M1c	G3	*KRAS*	Synchronous	None	Primary surgery + CRS	OXA low dose	ND	^c^26	-
C6	M	41	Sigmoid	Intestinal mucinous adenocarcinoma	pT4 N1c M1c	G3	*KRAS; TP53; APC; SMAD4*	Synchronous	Folfoxiri + Bevacizumab; Capecitabine + Bevacizumab	CRS/HIPEC	MMC	MMC	^a^8	Peritoneum, liver
PM1	F	67	Left	Intestinal adeonocarcinoma	pT4aN2bM1c	G2	*KRAS; TP53; APC*	Synchronous	Folfox + Bevacizumab 5-Fu + Bevacizumab	CRS/HIPEC	MMC	MMC + CDDP	21	None
PM2	M	52	Rectum	Mucinous adenocarcinoma	ypT3N0 M0	G2	*KRAS; TP53; APC*	15	Capox (adjv)	Diagnostic VLS	OXA low dose	OXA low dose	^a^10	Peritoneum
PM3	F	76	Rectum	Poorly differentiated mucinous adenocarcinoma	pT1N0 M0	G3	*KRAS*	25	None	Diagnostic VLS	MMC	ND	^b^3	-
PM4	M	43	Left	Signet ring cells mucinous adenocarcinoma	Tx Nx M1c	G2	*KRAS; TP53*	Synchronous	None	Diagnostic VLS	MMC	ND	^c^2	-
PM5	F	66	Right	Intestinal adenocarcinoma	pT3N1bM0	G3	*KRAS*	7	Capecitabine (adjv)	Diagnostic VLS	MMC	MMC	3	None
PM6	M	61	Right	Intestinal adenocarcinoma	Tx Nx M1c	G3	*BRAF; TP53; SMAD4;* *KDR*	Synchronous	None	Diagnostic VLS	Resitant	ND	^c^3	-
PM7	F	44	Sigmoid	Intestinal adenocarcinoma	pT4N1bM0	G2	*TP53; APC*	9	Folfox (adjv); Folfox; Folfiri Aflibercet	CRS/HIPEC	MMM + CDDP	MMC	^a^22	Lung

*CRCPM* Colorectal Cancer Peritoneal Metastasis, *PDO* Patient-derived Organoid, *CT* Chemotherapy, *CRS* Cytoreductive Surgery, *PM* Peritoneal Metastasis, *MMC* Mitomicyn-C, *CDDP* Cisplatin, *OXA* Oxaliplatin, *CRS/HIPEC* Cytoreductive Surgery plus Hyperthermic IntraPeritoneal Chemotherapy, *5-Fu* 5-Fluorouracil, *VLS* Video-Laparoscopy, *Adjv* Adjuvant, *Maint* Maintainment, *FOLFOX, FOL* Folinic Acid, *F* Fluorouracil, *OX* Oxaliplatin, *FOLFIRI*, *FOL* Folinic acid, *F* Fluorouracil, *IRI* Irinotecan

^a^recurrence/relapse after CRS/HIPEC

^b^disease progression out of peritoneum

^c^death of disease before CRS/HIPEC

### Development of CRCPM-derived PDO

Twelve PDO lines, C1, C2, C3, C4, C6, PM1, PM2, PM3, PM4, PM5, PM6 and PM7 were used (Table 1). PDO were developed as previously reported [18, 19]. Briefly tumour tissues were cut into small specimens, washed ten-times with cold PBS 1 X supplemented with 50 ng/ml gentamicin and then digested with 1 mg/ml collagenase type II for 1 h at 37 °C. The cells were recovered, resuspended in commercial basement membrane matrix (Matrigel, Corning, USA) and dispensed into a 24 multi-well (40 µl/well), that was filled with 500 µl of culture medium. PDOs were grown in basal cell culture medium consisting of Advanced DMEM-F12 (Thermo Fisher Scientific, USA) supplemented with the colonic-specific factors to mimic the corresponding niche conditions [18] (Supplementary Table S1 and Supplementary Material and Methods). All the factors were added as reported in Supplementary Table S1. PDOs were incubated at 20% of O_2_ and 5% CO_2_. After 2/3 weeks, the optimal PDO culture medium conditions were determined separately for each PDO culture (Supplementary Table S2 and Supplementary Material and Methods). PDOs were split every 3–4 weeks as follows: they were mechanically removed from the Matrigel by pipetting, incubated in Cell Recovery Solution (Corning, USA) for 1 h at 4 °C, washed trice with ice-cold PBS and seeded as described above. Concordance of PDOs with their tumor of origin was evaluated as described in Varinelli et al*.* [18].

### Cancer-associated fibroblast (CAF) isolation and characterization

CAFs were isolated from patient’s tissue as described in Walterskirchen et al*.* [20]. Tumor tissues were cut into small pieces (~ 3–4 mm), washed five times with PBS 1 × supplemented with 50 ng/ml gentamicin, and digested with 1 mg/ml collagenase type B for 1 h at 37 °C. Digested tissues were filtered with a 100 µm cell strainer and the filtered cells were centrifuged at 450 g for 5 min and then seeded into a 6 wells multiwell (~ 350,000 cells/well) and cultured in EGM™ MV2 medium (PromoCell GmbH, Germany) as in Walterskirchen et al*.* [20]. After 2–3 days the cells in suspension were removed and the attached fibroblasts were expanded and cultured in EGM™ MV2 medium. CAFs were characterized by western blotting analysis using the following antibodies: anti-SMA, anti-FAP, anti-E-Cadherin and anti-Vinculin as previously reported [18] (Supplementary Material and Methods and Supplementary Table S3).

### Chemotherapeutic agents used for HIPEC simulation

Mitomycin-c (MMC) (Kyowa Kirin Co., Ltd., Japan), oxaliplatin (L-OHP), cisplatin (CDDP), and doxorubicin (DOX) (Accord Healthcare Limited, UK) were used for the in vitro simulation of HIPEC treatment. MMC was dissolved in dimethyl sulfoxide (DMSO) to obtain a 60 mM stock solution. L-OHP, CDDP and DOX were diluted in physiological solution (0.45% sodium chloride and 2.5% glucose) to obtain a 15 mM, 3.32 mM and 3.68 mM stock solution, respectively. The doses of the drugs administered to the PDOs were the same as those of the patients, but converted to μM instead of mg/m^2^ and mg/L (due to the smaller volume of the PDOs compared to the peritoneal cavity of the patient, Table 2 and Supplemetary Table S4).

**Table 2:** The five main HIPEC schemes used in the clinical practice with the corresponding cytotoxic drugs adopted. Clinically relevant concentrations, dilutions, perfusion time and hyperthermia conditions were obtained from recorded perfusion data from the Peritoneal Malignancy Unit of Fondazione IRCCS Istituto dei Tumori di Milano. The mechanism of action of each cytotoxic drugs, the synergistic effect with heat and penetration index are also reported

HIPEC schemes							
**Scheme**	**Drug/s**	**Mechanism of action**	**Clinically relevant dose**	**Synergistic with heat**	**Penetration index (mm)**	**Perfusion time (min)**	**T (C°)**
#1	Mitomycin-C	Antitumor antibiotic (methylazirinopyrroloindoledione antineoplastic)	35 mg/m^2^	Yes	2	60	42.5
#2	Mitomycin-C + Cisplatin	Mitomycin-C: Antitumor antibiotic (methylazirinopyrroloindoledione antineoplastic) Cisplatin: Alkylating agent	Mitomicyn-C: 3.5 mg/m^2^ Cisplatin: 25 mg/m^2^	Yes	Mitomycin-C: 2 Cisplatin: 1–3	60	42.5
#3	Doxorubicin + Cisplatin	Doxorubicin: Antitumor antibiotic (anthracycline topoisomerase inhibitor) Cisplatin: Alkylating agent	Doxorubicin: 15 mg/L Cisplatin: 43 mg/L	Yes	Doxorubicin: 4–6 cell layers Cisplatin: 1–3	90	42.5
#4	Oxaliplatin_low-dose_	Alkylating agent	200 mg/m^2^	Yes	1–2	120	42.5
#5	Oxaliplatin_high-dose_	Alkylating agent	460 mg/m^2^	Yes	1–2	30	42.5

### In vitro simulation of HIPEC on CRCPM-derived PDO and IC_50_ evaluation

To determine the IC_50_ value of drugs and relative schemes, 5 × 10^3^ PDOs were suspended in culture medium and seeded in 96-well plates (100 µl/well, Costar 3904; Corning, USA) previously coated with 40 µl of Matrigel (30 – 50 PDO/well). After two days, PDOs were incubated with the different drugs. Briefly, all drugs were diluted at the working concentration in basal cell culture medium (DMEM-F12 supplemented with 15 mM HEPES, 2 mM GlutaMAX and 50 mg/ml of gentamicin and amphotericin B respectively). The final solvent concentration was < 0.1% for all samples, including controls (DMSO for MMC and physiological solution for the other drugs). PDOs were incubated at 42.5 °C or at 37 °C in 100 µl of basal cell culture medium containing the drugs, in a cell culture incubator for the specific time required for each HIPEC scheme (Table 2). Time and temperature used for the HIPEC simulation were the same as in the patient (Table 2). Afterwards, the drugs were removed by washing the wells three times with 1X PBS and cells were incubated for 48 h with cell growth medium. The drug doses routinely used during HIPEC perfusion, concentrations and perfusion time were calculated from the perfusion data recorded during the HIPEC surgery performed at our institute to treat CRCPM patients (Table 2 and Supplementary Table S4). The concentrations used to construct the dose–response curves were selected by scaling up and down the concentrations used for patients. For each HIPEC scheme, the clinically relevant drug concentrations were converted in µM for the in vitro experiments. Concentrations were obtained by normalizing each concentrations using 1 L as the final reference dilution volume; the clinically relevant doses were obtained using 1 L (scheme 1 and 2), 5 L (scheme 3) and 2 L (scheme 4 and 5) as the final reference dilution volumes (Supplementary Table S4). We used the same specific perfusion time already employed in the clinical practice to treat PDO (Table 2 and Supplementary Table S4). All the experiments were performed in triplicate.

### Co-culture development

Co-cultures were developed as in [21]. Briefly, four days after splitting, 200 PDOs were resuspended in 20 µl of co-culture matrix, consisting of 1.25 mg/ml Collagen-I (Corning, USA), neutralized with NaOH 1N and 25% Matrigel (Corning, USA) and seeded on a 48 wells plate. A total of 28.000 CAFs were resuspended in 200 µl of co-culture medium, consisting in DMEM-F12, 1 × B27 (Thermo Fisher, USA), 20 ng/ml human recombinant IGF protein, 10 ng/ml human recombinant FGF protien and 5 ng/ml human recombinant EGF protein (PromoCell GmbH, Germanyr). After 5 days 100 µl of fresh co-culture medium was added to each wells. After 8 days the co-cultures were formed.

### HIPEC simulation using co-culture models

CAFs were harvested using trypsin, centrifuged at 1200 rpm at 4 C° for 5 min, and the cell pellet was resuspended in EGM™ MV2 medium (PromoCell GmbH, Germany). CAFs were then seeded into a 96-Multiwell at a density of ~ 12,000 cells per well and let grow for 24 h. After that, PDOs resuspended in Matrigel were plated in 10 µl droplets directly onto CAFs (~ 50 PDO/well). Co-cultures formed by CRCPM-derived CAFs and PDO were then grown in co-culture medium (DMEM-F12, 1X B27 (Thermo Fisher, USA), 20 ng/ml human recombinant IGF protein, 10 ng/ml human recombinant FGF protein and 5 ng/ml human recombinant EGF protein (PromoCell GmbH, Germany)) for four days. HIPEC simulation and the IC_50_ calculation was performed as described above. All the experiments were performed in triplicate.

### Statistical analyses

Statistical analyses were performed using GraphPad Prism software (version 9.4.1 (676), GraphPad Software, San Diego, USA). Data are expressed as mean and SD. The best curve in the dose–response experiments was determined using *R*^2^ test. A two-tailed Student's *t*-test was used to compare paired groups. Differences among groups were evaluated using two-way ANOVA. A *p-*value < 0.05 was considered statistically significant. A heat-map for drug response sensitivity was assembled by calculating the corresponding Log of each median IC_50_ value for all PDO cultures treated with the different HIPEC schemes. The Log [IC_50_] values were then normalized and the patterns were aggregated column-wise into a matrix. The obtained heat-map was used to determine the relative sensitivity/resistance of each PDO line.

The data generated and analysed during the current study are available from the corresponding author on reasonable request.

## Results

### CRCPM-PDOs retain the main characteristics of their corresponding tumour of origin

**Fig. 2:**
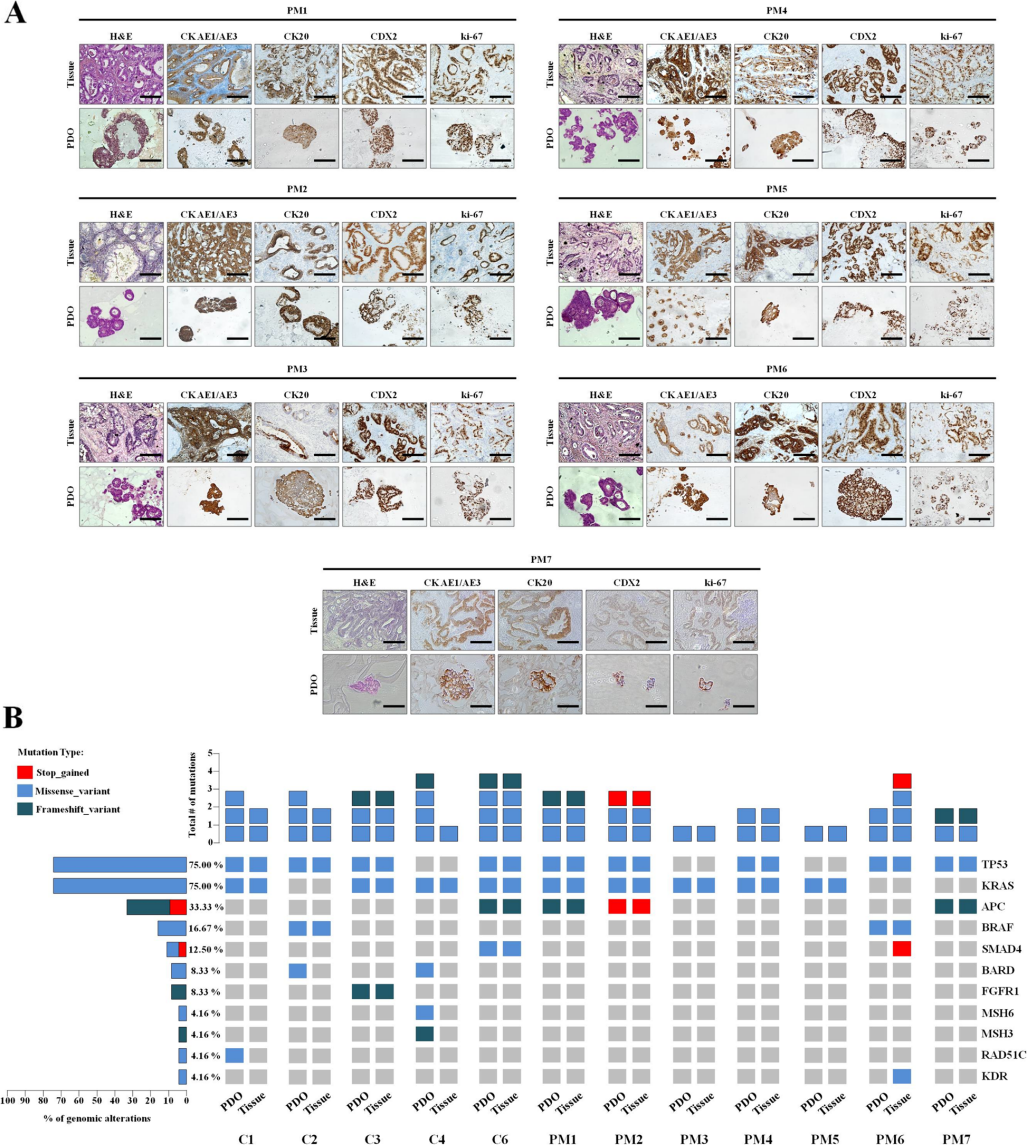
Characterization of CRCPM-derived PDOs. **A** Comparative histochemistry (HC) and IHC analysis of CRCPM-derived PDO and their tissue of origin using the CRC-derived protein markers CK, AE1/AE3, CK20, CDX2 and Ki67. Total magnification: 100 x. Scale bar, 100 µm. Surgical samples and the derived PM1, PM2, PM3, PM4, PM5, PM6 and PM7 PDOs (passage numbers: P10, P14, P9, P7, P11, P10 and P7 respectively) were developed from patients 4, 5, 6, 7, 8, 9 and 10 who underwent investigative laparoscopy for CRCPM. **B** Mutational analysis showing the concordance between PDOs and their corresponding tissues. On the left, percentage of genomic alterations detected across the samples analyzed (bottom) and total number of mutations/sample. Data from C1, C2, C3, C4 and C6 have been already published [18]. *CRCPM* Colorectal Cancer Peritoneal Metastasis, *PDO* Patient-derived, *CRC* Colorectal Cancer, *CK AE1/AE3* Cytokeratin EA1 and EA3, *CK20* Cytokeratin 20, *CDX2* Caudal Type Homeobox 2 protein, *Ki-67* marker of proliferation Kiel 67

The twelve CRCPM-derived PDOs used in the study include five already characterized lines (C1, C2, C3, C4 and C6) developed from tissue collected during CRS-HIPEC [18] and seven PDOs (PM1, PM2, PM3, PM4, PM5, PM6 and PM7) developed from tumour biopsies collected during laparoscopy intervention, characterized following the already established protocols (Supplementary Fig. S1) [18]. Two PDO lines grew in basal culture medium, four in the same medium enriched in the basic colonic-specific niche factors N-acetylcysteine, prostaglandin-E2 and gastrin-I; three other lines also required A83-01, SB202190 and Noggin, while two PDO line A83-01 plus Noggin. Only one PDO line grew in medium enriched in the basic colonic-niche factors plus A83-01 inhibitor (Supplementary Fig. S2). The PDO cultures showed specific morphology in vitro, with tubular formation and typical glandular features observed in the corresponding surgical sample, such as signet-ring cells, nest-like growth pattern, nuclear atypia, cuboidal nuclear morphology and pleomorphism (Fig. 2A). All PDOs expressed major colorectal cancer specific markers, such as CK AE1/AE3, CK20 and CDX2 as the corresponding clinical samples (Fig. 2A). Indeed, they were positive for Ki-67 expression, mirroring an active proliferative status (Fig. 2A). These results indicate that CRCPM-derived PDOs closely mimic the histology of the tissue of origin. NGS analysis also confirmed mutational status concordance between PDOs and tissue of origin (Fig. 2B and Supplementary Table S5).

### PDOs response to HIPEC simulation

**Fig. 3:**
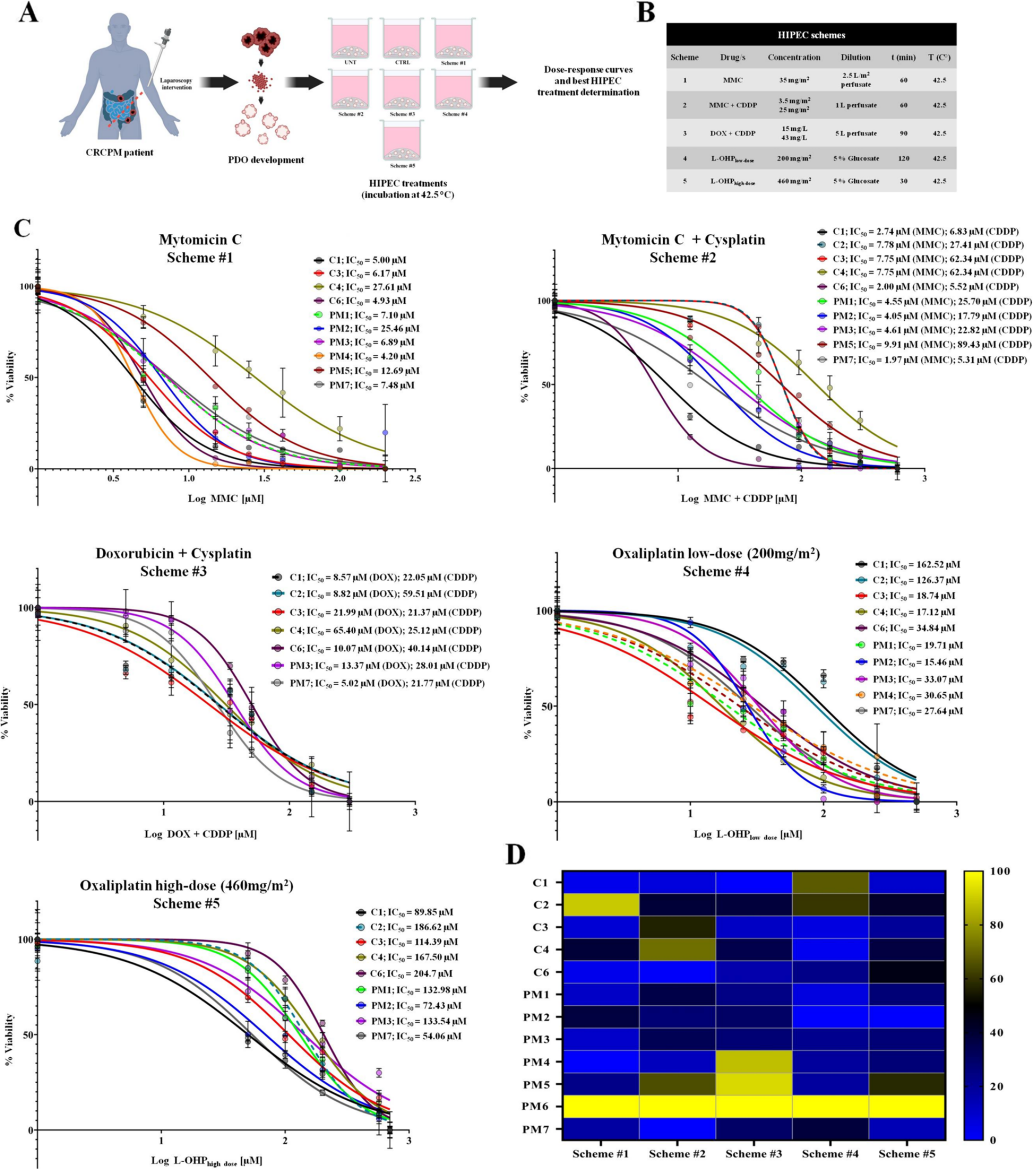
PDO response to HIPEC simulation. **A** Schematic representation of the protocol used to perform the dose–response curves experiments. **B** Description the five HIPEC schemes tested. **C** Dose–response curves of CRCPM-derived PDOs illustrating the variation in sensitivity to MMC (scheme 1), MMC + CDDP (scheme 2), DOX + CDDP (scheme 3), L-OHP_low-dose_ (scheme 4) and L-OHP_high-dose_. (scheme 5). **D** Normalized Log of IC_50_ mean values heat-map for standard HIPEC treatments (yellow: no response; blue: good response). *HIPEC* Hyperthermic IntraPEritoneal Chemotherapy, *CRCPM* Colorectal Cancer Peritoneal Metastasis, *PDO* Patient-derived Organoid, *MMC* Mitomycin-C, *CDDP* Cisplatin, *DOX* Doxorubicin, *L-OHP* Oxaliplatin, *IC*_*50*_ Half maximal inhibitory concentration

To evaluate the efficacy of PDOs as a model for assessing the most effective HIPEC scheme in a patient-personalized manner, we tested several therapeutic schemes commonly used for the treatment of CRCPM (Fig. 3, Supplementary Fig. S3) [2]. The clinically relevant dose for each scheme was derived converting the concentration of perfusion to molarity (Supplementary Table S4). Based on the converted doses, the half maximal inhibitory concentration (IC_50_) related to each HIPEC scheme, was determined in all PDOs. Drug response ranged notably among the different PDOs (Fig. 3C-D, Supplementary Fig. S3). The MMC IC_50_ ranged from 4.20 µM to 25.46 μM for most PDO lines, except for C2 and PM6, which resulted as non-responders (Fig. 3C-D, scheme 1, Table 3 and Supplementary Fig. S3). The IC_50_ for MMC administrated in combination with CDDP ranged from 1.97 µM MMC + 5.31 µM CDDP to 9.91 μM MMC + 89.43 µM CDDP, where C6 and PM7 were the most sensitive PDOs and PM6 did not respond (Fig. 3C-D, schemes 2, Table 3 and Supplementary Fig. S3). Concerning CDDP treatments combined with DOX, IC_50_ ranged from 5.02 µM CDDP + 21.77 µM DOX for PM7, to 25.12 µM CDDP + 65.40 µM DOX for C4. Data showed that C1, C2, C3, C4, C6, PM3 and PM7 partially responded to CDDP combined with DOX, while PM1, PM2, PM4, PM5 and PM6 were resistant (Fig. 3C-D, scheme 3, Table 3 and Supplementary Fig. S3).

**Table 3:** Estimated half-maximal inhibitory concentrations (IC_50_) for MMC (scheme 1), MMC + CDDP (scheme 2), DOX + CDDP (scheme 3), L-OHP_low-dose_ (scheme 4) and L-OHP_high-dose_ (scheme 4) in CRCPM-derived PDO. For each PDO line, the IC_50_ corresponding to the best HIPEC scheme is reported in bold

IC_50_ Values for HIPEC schemes					
**PDO Line**	**IC**_**50**_** scheme 1 (µM)**	**IC**_**50**_** scheme 2 (µM)**	**IC**_**50**_** scheme 3 (µM)**	**IC**_**50**_** scheme 4 (µM)**	**IC**_**50**_** scheme 5 (µM)**
C1	5.00	2.74 (MMC) 6.83 (CDDP)	8.57 (DOX) 22.05 (CDDP)	162.52	89.85
C2	NA	7.78 (MMC) 27.41 (CDDP)	8.82 (DOX) 59.51 (CDDP)	126.37	186.62
C3	6.17	7.55 (MMC) 62.34 (CDDP)	21.99 (DOX) 21.37 (CDDP)	18.74	114.39
C4	27.61	17.17 (MMC) 111.5 (CDDP)	65.40 (DOX) 25.12 (CDDP)	17.12	167.5
C6	4.93	2 (MMC) 5.52 (CDDP)	10.07 (DOX) 40.14 (CDDP)	34.84	204.7
PM1	7.10	4.55 (MMC) 25.70 (CDDP)	7.99 (DOX) 26.16 (CDDP)	19.17	132.98
PM2	25.46	4.05 (MMC) 17.79 (CDDP)	9.68 (DOX) 37.62 (CDDP)	15.46	72.43
PM3	6.89	4.61 (MMC) 22.82 (CDDP)	13.37 (DOX) 28.01 (CDDP)	33.07	133.54
PM4	4.20	2.27 (MMC) 9.98 (CDDP)	NA	30.65	134.15
PM5	12.69	9.91 (MMC) 89.43 (CDDP)	NA	26.93	269.25
PM6	NA	NA	NA	NA	NA
PM7	7.48	1.97 (MMC) 5.31 (CDDP)	5.02 (DOX) 21.77 (CDDP)	27.64	54.06

*MMC* Mitomycin-C, *CDDP* Cisplatin, *DOX* Doxorubicin, *L-OHP* Oxaliplatin, *CRCPM* Colorectal Cancer Peritoneal Metastasis, *PDO* Patient-derived Organoid, *HIPEC* Hyperthermic IntraPEritoneal Chemotherapy

Considering oxaliplatin-based schemes, the IC_50_ for the low-dose regimen (200 mg/m^2^ for 120 min) ranged from 15.46 µM to 162.52 µM, PDOs showing the highest sensitivity were C4 and PM2 (Fig. 3C-D, scheme 4, Table 3 and Supplementary Fig. S3). Conversely, the IC_50_ for the high-dose regimen (460 mg/m^2^ for 60 min) ranged on higher values, from 54.06 µM for PM7 to 269.25 µM for PM5 (Fig. 3C-D, scheme 5, Table 3 and Supplementary Fig. S3). Moreover, PM6 PDO resulted resistant to both oxaliplatin-based schemes (Fig. 3C-D, schemes 4, 5, Table 3 and Supplementary Fig. S3).

### L-OHP_low-dose_ is more effective than L-OHP_high-dose_

We treated PDOs with both L-OHP administered at low dose (L-OHP _low-dose_) for 120 min and high dose (L-OHP _high-dose_) for 30 min (Fig. 3C-D, schemes 4, 5, Table 3 and Supplementary Fig. S3): L-OHP-based schemes were more effective in all PDO lines when administered at low dose for prolonged perfusion time (Fig. 3C-D, scheme 4, Table 3 and Supplementary Fig. S3). The IC_50_ value was lower for all PDOs when treated with L-OHP _low-dose_, with values ​​ranging from 15.46 µM for PM2 to 269.25 µM for PM5 (Fig. 3C-D, Table 3 and Supplementary Fig. S3). Overall, L-OHP _low-dose_ scheme was the most effective for C4 and PM2 PDOs; the other PDOs, while responding to this scheme, showed greater sensitivity to MMC-based schemes, either alone or in combination with CDDP (Fig. 3C-D, Table 3 and Supplementary Fig. S3).

### PDO models as a tool for simulating HIPEC treatments

**Fig. 4:**
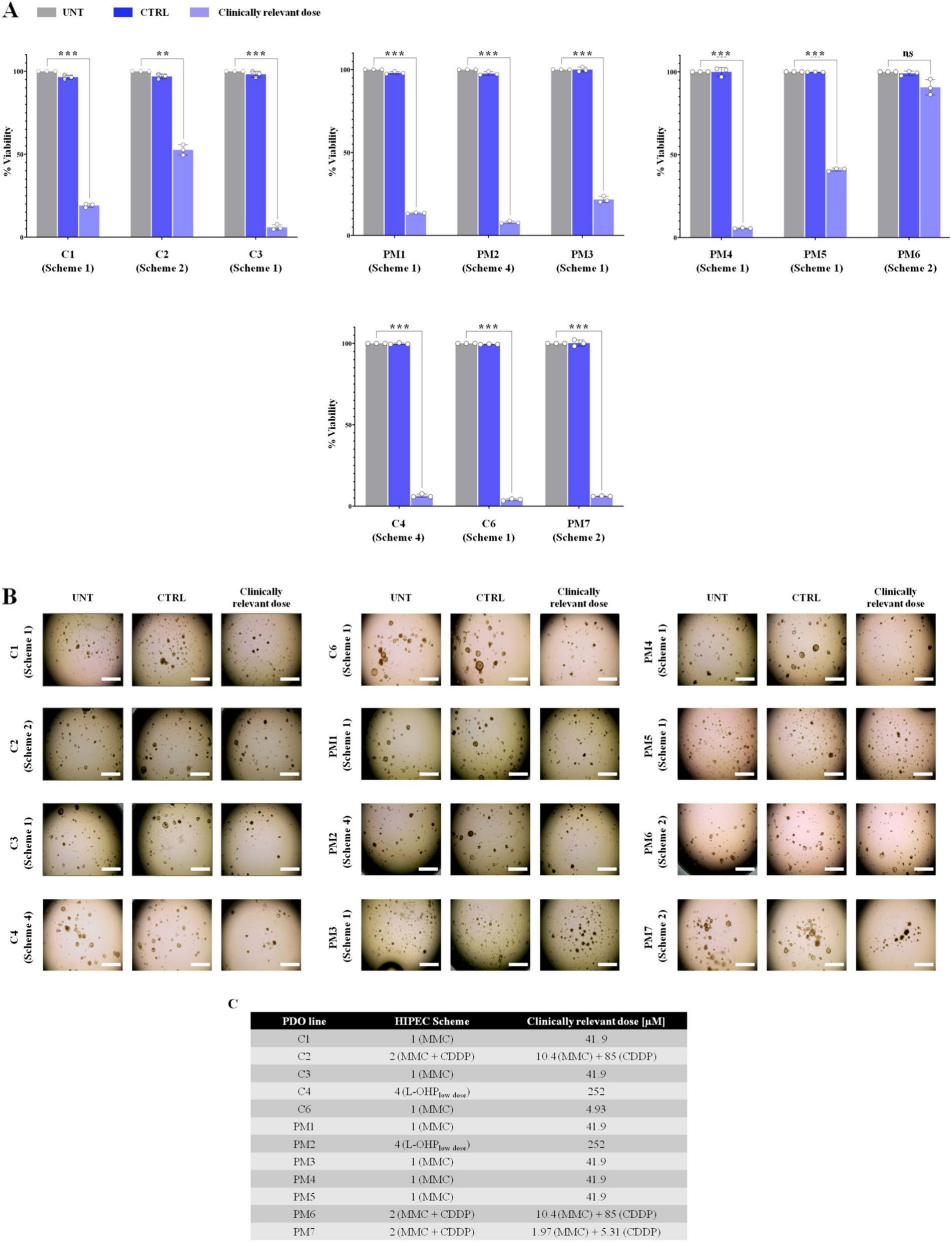
The PDO model system is an effective tool for tailor HIPEC treatments. **A** Percentage of live PDOs after HIPEC treatments, measured as chemiluminescent signal of the concentration of intracellular ATP. PDOs were treated with the most effective HIPEC scheme using drug concentrations corresponding to the calculated clinically relevant dose. Data are presented as median and SD and the experiments were performed in triplicate. *Student’s t-test* (**p < 0.01; ***p < 0.001). UNT: PDO treated with medium only at 42.5 °C; CTRL: PDO treated with 0.1% of physiological solution at 42.5 °C. **B** Micrographs showing PDOs treated with the best HIPEC scheme using the calculated clinically relevant dose. Total magnification: 40 x. Scale bar: 100 µM. UNT: PDO treated with medium only at 42.5 °C; CTRL: PDO treated with 0.1% of physiological solution at 42.5 °C. **C** The best HIPEC treatment for each PDO line analysed; calculated clinically relevant dose for each scheme reported in µM. *PDO* Patient-derived Organoid, *HIPEC* Hyperthermic IntraPEritoneal Chemotherapy, *ATP* Adenosine triphosphate, *SD* Standard Deviation, *UNT* Untreated, *CTRL* Control

We determined the most promising HIPEC treatment for each PDO line (Fig. 3 and Fig. 4A) by interpolating the IC_50_ value from the dose–response curves on the x-axis (concentration axis, Fig. 3C). The scheme that exhibited the lowest concentration value, combined with an *R*^2^ value ≥ 0.90, was chosen as the most efficient scheme for a specific PDO (Fig. 3C, Fig. 4, Table 3, Table 4 and Supplementary Table S4). Response to the respective best HIPEC treatment of nine PDO lines (C1, C3, C4, C6, PM1, PM2, PM3, PM4 and PM7) measured as the percentage of viable cells ranged from about 5% in C3 and PM4 to about 20% in C1 and PM3 PDOs (Fig. 4A-B, Supplementary Fig. S4). C2 and PM5 PDOs showed only partial response, with about 50% viable cells after the corresponding best HIPEC schemes (1 and 2, respectively) (Fig. 4, Supplementary Fig. S4). PM6 PDO line was resistant to all HIPEC schemes, being slightly responsive only to scheme 2 (Fig. 4, Supplementary Fig. S4). Notably, the viability values showed that the clinically relevant doses of all HIPEC schemes were insufficient to eliminate all cancer cells (Fig. 4A).

**Table 4:** *R*^2^ values calculated for each PDO line treated with the five HIPEC schemes

PDO line	*R*^2^ Scheme #1	*R*^2^ Scheme #2	*R*^2^ Scheme #3	*R*^2^ Scheme #4	*R*^2^ Scheme #5
C1	0.97	0.97	0.93	0.90	0.98
C2	0.27	0.97	0.93	0.91	0.96
C3	0.99	0.97	0.94	0.94	0.97
C4	0.94	0.94	0.90	0.99	0.97
C6	0.99	0.99	0.99	0.98	0.99
PM1	0.96	0.95	0.82	0.93	0.91
PM2	0.93	0.97	0.20	0.89	0.95
PM3	0.96	0.96	0.90	0.93	0.93
PM4	0.97	0.89	0.17	0.90	0.008
PM5	0.99	0.97	0.79	0.99	0.89
PM6	0.12	0.23	0.16	0.09	0.13
PM7	0.96	0.97	0.98	0.99	0.99

*PDO* Patient-derived Organoid, *HIPEC* Hyperthermic IntraPEritoneal Chemotherapy

### The PDO-based assay is robust and reproducible to determine the best HIPEC treatment

**Fig. 5:**
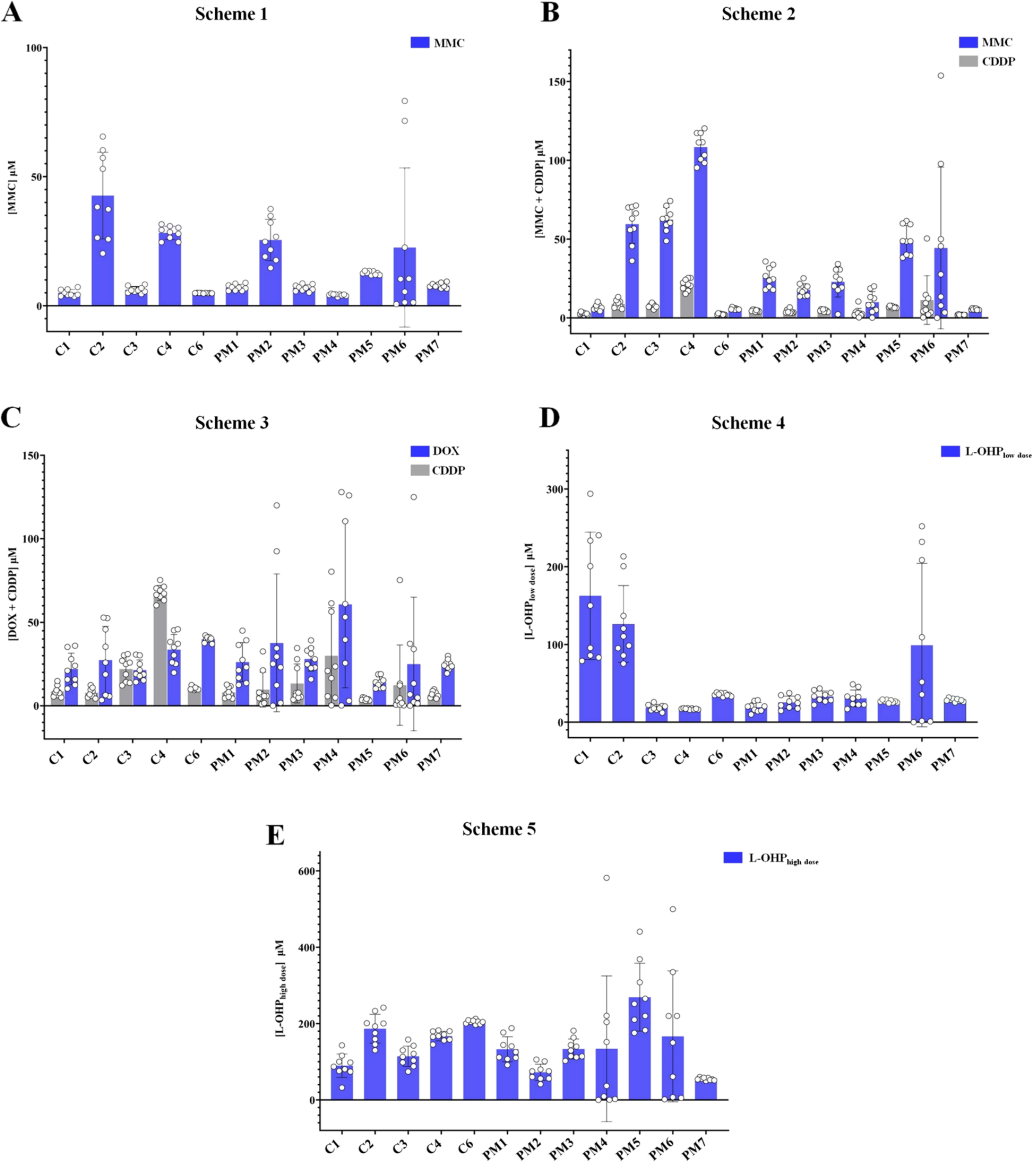
The PDO based assay is robust and reproducible to determine the best HIPEC treatment. Estimated variation among different in vitro HIPEC experiments for (**A**) MMC, (**B**) MMC + CDDP, (**C**) CDDP + DOC, (**D**) L-OHP_low dose_, and (**E**) L-OHP_high dose_ schemes. Bars show SD in IC_50_ values, and circles indicate estimated single IC_50_ values. *HIPEC* Hyperthermic IntraPEritoneal Chemotherapy, *MMC* Mitomycin-C, *CDDP* Cisplatin, *DOX* Doxorubicin, *L-OHP* Oxaliplatin, *SD* Standard Deviation, *IC*_*50*_ Half maximal inhibitory concentration

We compared the IC_50_ values ​​obtained from several independent dose–response experiments in which the twelve PDO lines were treated with the five different HIPEC schemes tested (Fig. 5). PDOs treated with their best HIPEC scheme, as determined through their respective IC_50_ values ​​ (Fig. 4 and Table 3), showed limited variation in IC_50_ values, indicating good reproducibility and effectiveness in determining the best treatment scheme when compared with other HIPEC treatments (Fig. 5), also showing an *R*^2^ value ≥ 0.90 (Table 4). Treatments based on MMC alone, MMC in combination with CDDP and L-OHP_low-dose_ (Fig. 5A-B-D), showed less overall variability in IC_50_ values ​​than treatments based on CDDP in combination with DOX and those based on L-OHP_high-dose_ (Fig. 5C-E), where the corresponding *R*^2^ values were ≤ 0.90 (Table 4). These results indicate that HIPEC schemes based on the use of MMC alone or in combination with CDDP and protocols based on L-OHP_low-dose_ administration are more effective. In contrast, HIPEC schemes based on treatment with CDDP + DOX and L-OHP_high-dose_ showed marked variability in IC_50_ values, suggesting their lower efficacy in treating CRCPM disease (Fig. 5). Finally, PM6 PDO line has high variability in IC_50_ values with all five HIPEC schemes (*R*^2^ ≤ 0.23) (Fig. 5 and Table 4).

### Hyperthermia synergizes with HIPEC drugs and increases the impact of treatment on PDO viability and apoptosis

**Fig. 6:**
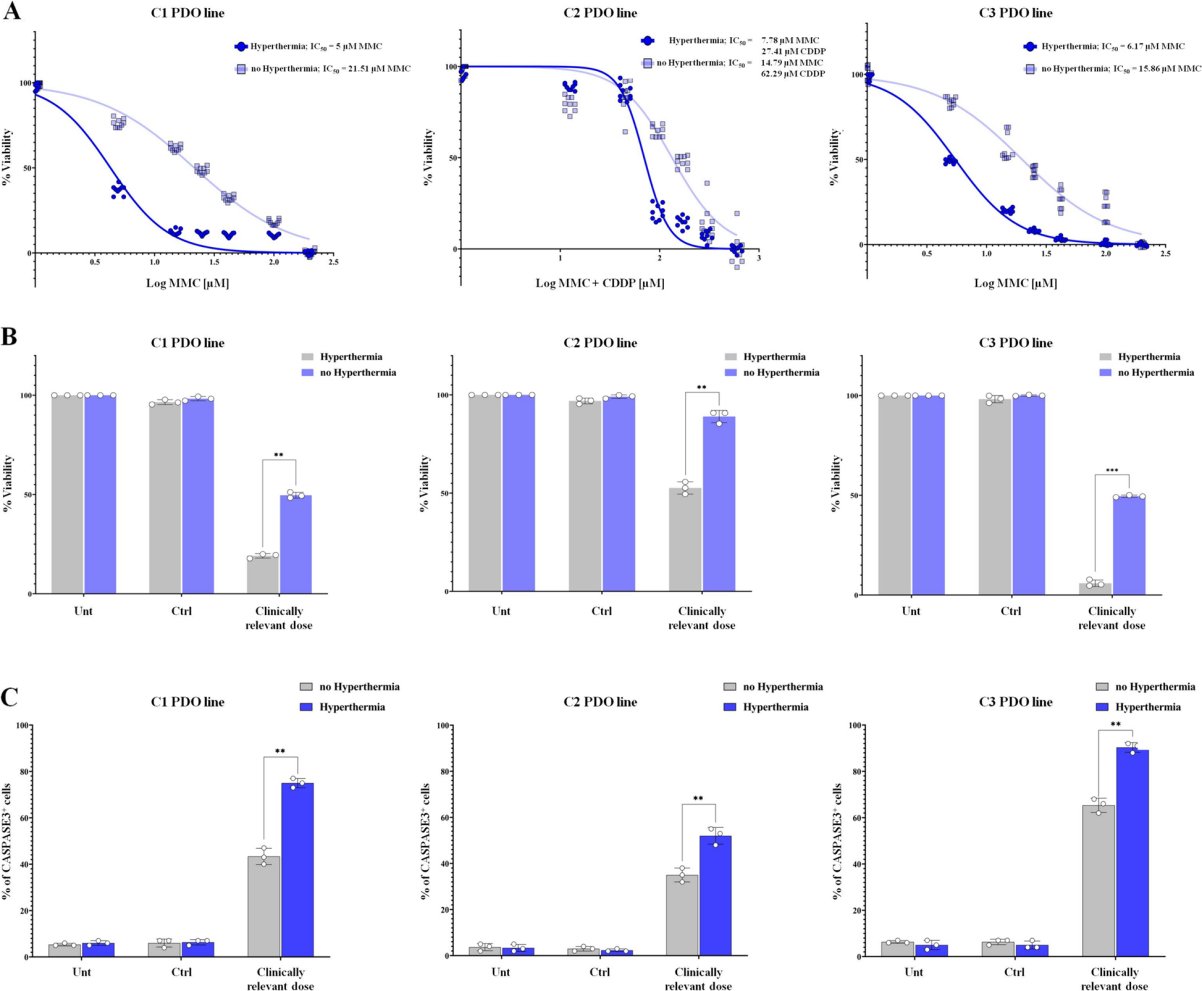
Hyperthermia synergized with HIPEC drugs enhancing treatment impact on PDO viability and apoptosis. **A** Dose–response curves of CRCPM-derived PDOs (C1, C2 and C3) illustrating the variation in sensitivity to MMC (scheme 1, C1 and C3 PDO lines) and MMC + CDDP (scheme 2, C2 PDO line) in hyperthermia and non-hyperthermia conditions. **B** percentage of live PDOs after HIPEC treatments, measured as chemiluminescent signal of the concentration of intracellular ATP. PDOs were treated with the most effective HIPEC scheme using drug concentrations corresponding to the calculated clinically relevant dose and in hyperthermia condition and not. Data are presented as median and SD and the experiments were performed in triplicate. *Student’s t-test* (**p < 0.01; ***p < 0.001). UNT: PDO treated with medium only at 42.5 C or 37 C; CTRL: PDO treated with 0.1% of physiological solution at 42.5 C or 37°C. **C** percentage of CASPASE3^+^ cells in PDOs after HIPEC treatments, measured as chemiluminescent signal of the activity of CASAPSE3. PDOs were treated with the most effective HIPEC scheme using drug concentrations corresponding to the calculated clinically relevant dose and in hyperthermia condition and not. Data are presented as median and SD and the experiments were performed in triplicate (**p < 0.01; ***p < 0.001; ANOVA, hyperthermia Vs non-hyperthermia). UNT: PDO treated with medium only at 42.5 C or 37 C; CTRL: PDO treated with 0.1% of physiological solution at 42.5 C or 37 C. *CRCPM* Colorectal Cancer Peritoneal Metastasis, *PDO* Patient-derived Organoid, *MMC* Mitomycin-C, *CDDP *Cysplatin, *HIPEC* Hyperthermic IntraPEritoneal Chemotherapy, *ATP* Adenosine triphosphate, *SD* Standard Deviation, *UNT* Untreated, *CTRL* Control, *CASPASE3* Cysteine-Aspartic Acid Protease 3

Intraperitoneal chemotherapy is given under hyperthermia to enhance the effect of chemotherapy drugs against neoplastic cells. We tested whether hyperthermia could synergize with drugs commonly used for HIPEC treatments in our models. Three PDO lines (C1, C2 and C3) were treated with the corresponding best-determined HIPEC scheme, under hyperthermia and non-hyperthermia conditions (Fig. 6). Under non-hyperthermia conditions, the three PDO lines showed a clear shift in the dose–response curves, with a significant increase in IC_50_ values, which increased from 5 µm MMC to 21.51 µM MMC for C1, from 7.78 µM MMC and 14.79 µM CDDP to 27.41 µM MMC and 62.29 µM CDDP for C2 and from 6.17 µM MMC to 15.86 µM for C3, respectively (Fig. 6A and Supplementary Fig. S5). Also, the percentage of viable cells went from 20 to 50% for C1, from 50 to 85% for C2 and from 5 to 50% for C3 PDO line, respectively (Fig. 6B, **p < 0.01*;* ***p < 0.001, *ANOVA*). In addition, PDO lines treated at the corresponding clinically relevant dose under non-hyperthermia conditions showed significantly fewer CASPASE3 positive cells than PDOs treated with hyperthermia, ranging from 43% instead of 78% for C1, 36% instead of 53% for C2, and 65% instead of 95% for line C3, (Fig. 6C, **p < 0.01; ANOVA). Overall, these data indicate the relevance of hyperthermia in HIPEC schemes.

### CAFs impact HIPEC treatments by improving PDO viability and reducing CASPASES activity

**Fig. 7:**
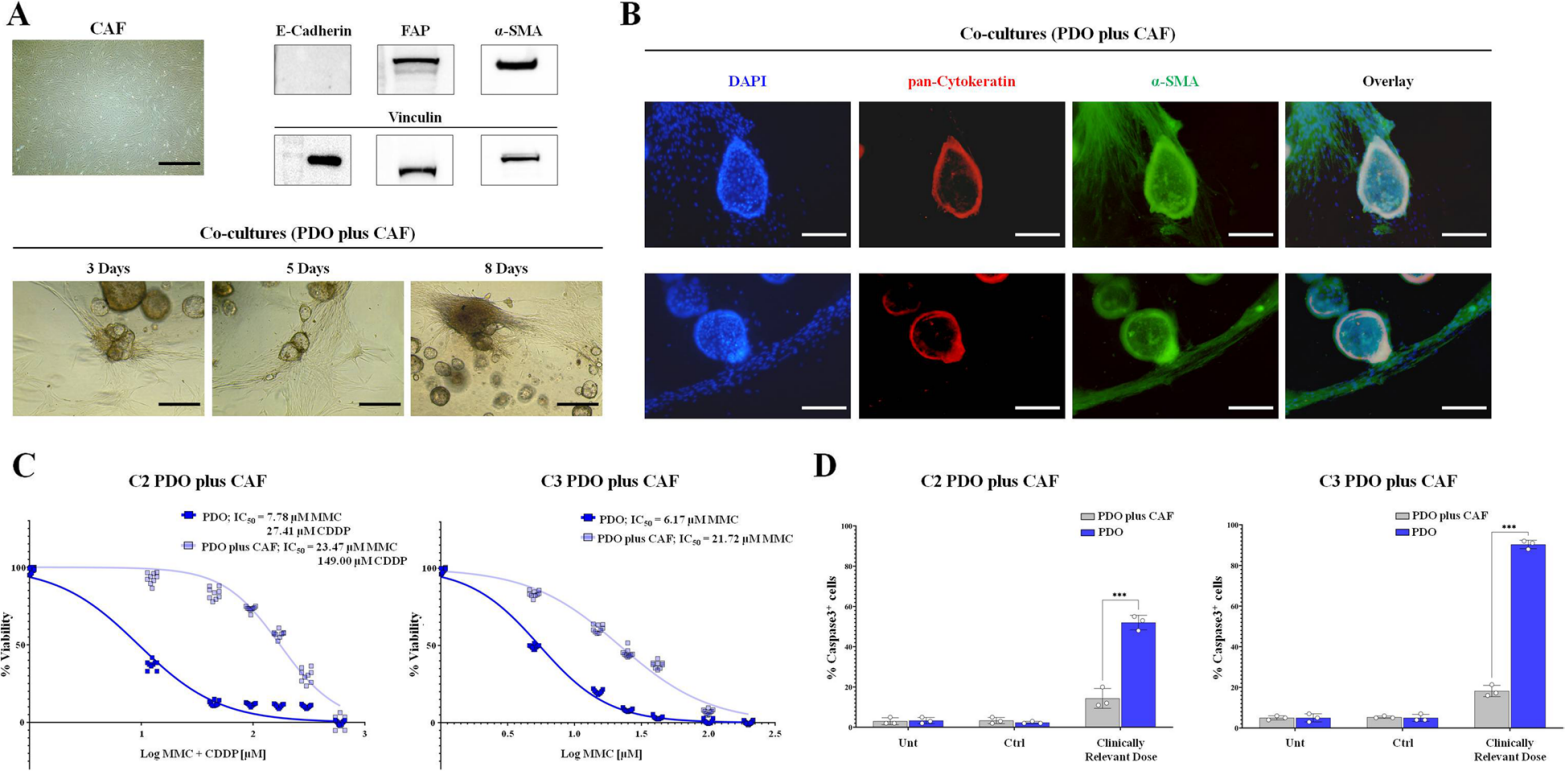
CAFs impact HIPEC treatments enhancing PDO viability and reducing CASPASES activity. **A** micrograph showing CRCPM-derived CAF (total magnification 40 x, scale bar: 200 µM). Western blotting analysis of E-Cadherin, FAP and α-SMA expression in CRCPM-derived CAFs. Vinculin was used as loading control. Bright field images showing CRCPM-derived PDO cultured with CAF after 3, 5 and 8 days (total magnification: 100 x, scale bar: 100 µM). **B** Fluorescence images showing CRCPM-derived PDO co-cultured with CAF. Cells were stained with DAPI: blue, α-SMA: green, and pan-cytokeratin: red (total magnification 100 x, scale bar 100 µM). **C** Dose–response curves of CRCPM-derived PDO (C2 and C3) illustrating the variation in sensitivity to MMC + CDDP (scheme 2, C2) and MMC (scheme 1, C3 PDO line) cultured with CAF or alone. **D** Percentage of CASPASE3 positive cells in PDOs after HIPEC treatments, measured as chemiluminescent signal of the activity of CASPASE3. PDOs were treated with the most effective HIPEC scheme using drug concentrations corresponding to the calculated clinically relevant dose and presence of CAF or not. Data are presented as median and SD and the experiments were performed in triplicate. (**p < 0.01; ***p < 0.001; ANOVA, co-culture group Vs monoculture group). *CRCPM* Colorectal Cancer Peritoneal Metastases, *CAF* Cancer-associated Fibroblast, *FAP* Fibroblast Activated Protein, *α-SMA* Smooth Muscle Actin alpha, *PDO* Patient-derived Organoid, *DAPI* 4′,6-diamidino-2-phenylindole, *MMC* Mitomycin-c, *CDDP* Cysplatin, *CASPASE3* Cysteine-Aspartic Acid Protease 3, *HIPEC* Hyperthermic IntraPEritoneal Chemotherapy, *ANOVA* Analysis of Variance

We established CAF cultures from patients with CRCPM (Fig. 7A). Isolated CAFs presented the typical fibroblast morphology with a stromal-track like organization (Fig. 7A), expressing high levels of both the myofibroblast marker FAP and the CAF marker α-SMA and they were negative for the epithelial marker E-Cadherin (Fig. 7A, Supplementary Fig S6). Next we developed co-cultured models using PDOs (C2 and C3 PDO lines) and CAFs using the basal PDO medium (DMEM-F12, HEPES, L-glutamin) and added different supplement able to sustain PDO and fibroblast growth, such as B27, FGF and EGF (Co-culture medium, see *“*Co-culture development*”* in Material and Methods section). The co-cultured CAFs organized into a continuous circle surrounding the PDOs matrix dome, and after 3 days of culture invaded the dome (Fig. 7A, lower panel). After 5 days, within the dome, CAFs organized into stromal tracks and PDOs reorganized along these tracks (Fig. 7A, lower panel). After 8 days of co-culture, the PDOs formed aggregates surrounded by CAF fibers (Fig. 7A, lower panel). IF analysis with anti α-SMA and anti pan-cytokeratin, performed to visualize CAF and epithelial-derived cells, showed the presence of clusters of PDOs residing in α-SMA-positive CAF fields with co-localization of tumor-derived cells and CAF fibers, indicating the development of a single structure (Fig. 7B).

**Fig. 8:**
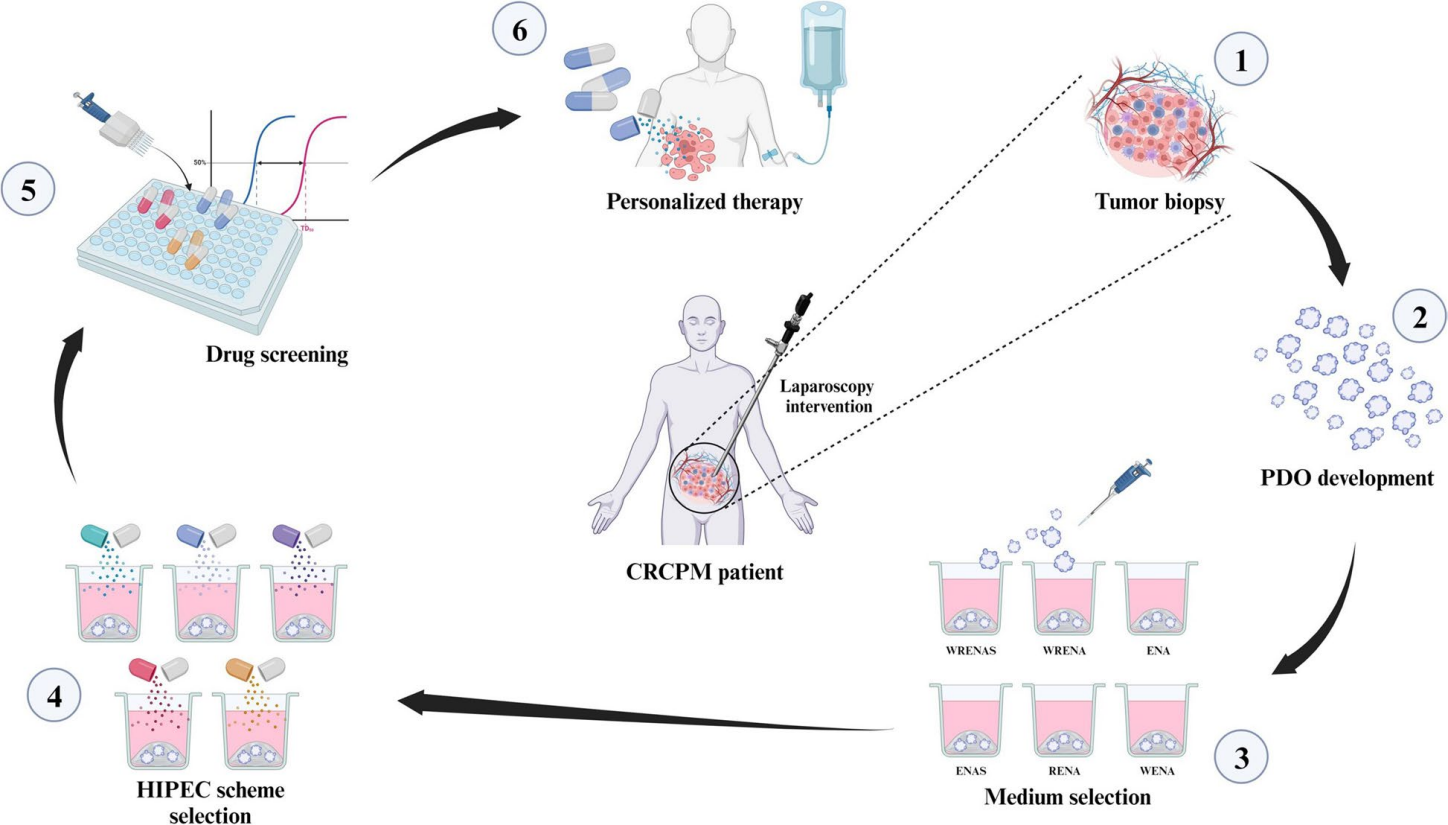
PDO technology as a tool for tailored drug screening assays. The figure summarizes the pipeline for using CRCPM-PDOs to perform personalized therapies. *CRCPM* Colorectal Cancer Peritoneal Metastasis, *PDO* Patient-derived Organoid, *WRENAS*, *W* Wnt family member 3A, *R* R-spondin 1 protein, *E* Epiderma Grow Factor, *N* Noggin protein, *A A83-01* – anti-p38 inhibitor, *S SB 202190* – anti-ROCK inhibitor, *HIPEC* Hyperthermic IntraPEritoneal Chemotherapy

For two PDO lines (C2 and C3), we tested the best HIPEC scheme (scheme #2 and #1) on co-culture models with CAF. We determined IC_50_ concentrations and compared them with the corresponding IC_50_ values determined using PDO monocultures, observing a significant increase in IC_50_ values for both PDOs (Fig. 7C). Specifically, in presence of CAF, IC_50_ values increased from 7.78 µM to 23.47 µM for MMC and from 27.41 µM to 149.00 µM for CDDP in line C2 (Fig. 7C, right panel). C3 PDOs showed a similar trend, with a 3.5-fold increase in IC_50_ when grown in co-culture, with an IC_50_ of 21.72 µM instead of 6.17 µM when grown in monoculture (Fig. 7C, left panel). In addition, the presence of CAF strongly decreased CASPASE3 activation in C2 and C3 lines treated with their best HIPEC models, with a percentage of CASPAS3-positive cells of about 20% in both PDO lines instead of 50% and 90% in C2 and C3 grown in monoculture, respectively (Fig. 7D, ***p < 0.001, ANOVA) Fig. 8.

### Preliminary clinical results

Amongst the twelve patients included in this series, five underwent CRS/HIPEC (see Table 1). Two of them were included in our prospective phase II clinical study (Clinicaltrials.gov # NCT06057298) and treated with CDDP and MMC, respectively, based on the drug sensitivity tests performed on PDOs. For the remaining three, PDOs were developed from PM samples collected during the procedure of CRS/HIPEC and, consequently, chemo-sensitivity test results were not available to select HIPEC drug schedules. However, MMC was the most active drug for two patients, and the combination of MMC and CDDP for one, based on PDOs, and all of them were treated with MMC alone or combined with CDDP. Overall, at the last review of follow-up, three patients were free of peritoneal recurrences.

## Discussion

Currently, the choice of drug(s) to be administered intraperitoneally to treat CRCPM is based on pharmacokinetic and pharmacodynamic data, but we are unable to predict at an individual patient level the efficacy of a given antiblastic agent to destroy the residual tumor cells [22]. In addition, the Prodige 7 randomized clinical trial reported the ineffectiveness of the oxaliplatin-based HIPEC after optimal CRS, as compared with CRS alone in improving overall survival [4]. These results support the need to design new treatment approaches to increase HIPEC efficacy [23–25].

Most preclinical studies have been conducted using two-dimensional (2D) cell cultures and animal cancer models. However, 2D cell cultures do not capture the complexity and heterogeneity of the original tumor, and animal models are limited by species-related differences, often showing limited translational ability [26]. As a result, many drugs that appear to perform well in the preclinical phase fail in the later stages of clinical development [27]. On the contrary, PDO models provide a 3D context closer to the tumour and are patient-specific, representing an exciting step toward personalized medicine. In retrospective studies, PDOs responded to standard/clinical therapies mimicking the initial response exerted by patients treated with the same agents [16]*.* The twelve PDO lines we developed from CRCPM patients responded differently to the five HIPEC schemes tested, highlighting patients’ heterogeneity in treatment response. In addition, we found that standard doses of HIPEC cannot completely eradicate all cancer cells, as already observed in similar studies [28–30]. In our experiments, the short-duration high-dose oxaliplatin (L-OHP _high-dose_) regimen was significantly less effective than the long-duration low-dose oxaliplatin (L-OHP _low-dose_) regimen. This result could explain the poor clinical response observed in the Prodige7 trial, prompting consideration of whether the L-OHP _high-dose_ regimen should still be considered in clinical practice. MMC alone or combined with CDDP proved to be the most effective. Indeed, seven of the twelve PDO lines showed a pronounced cell death rate after treatment with MMC alone, PM7 PDO was responsive to MMC + CDDP regimen, and one of the two non-responder lines (C2) was partially responsive to treatment with MMC + CDDP. Among the five patients included in our study who had CRS/HIPEC, four were treated with MMC (one also in combination with CDDP), and at the last clinical follow-up only one peritoneal recurrence occurred. Our data are consistent with previous retrospective clinical series treated with MMC [31], providing promising support for the development of new studies specifically based on intraperitoneal administration of MMC. Moreover, the additional efficacy of CDDP in MMC-based schemes would benefit from evaluation through dedicated clinical trials.

Concerning PDOs not responding to MMC-based regimens, one (C4) showed sensitivity to L-OHP at low-dose regimen, confirming the value of this individualized therapeutic approach. The other (PM2) was derived from a patient enrolled in our prospective phase II clinical study and experienced peritoneal recurrence 10 months after CRS/HIPEC with L-OHP.

In line with data from the literature, where there is a clear global consensus on the adoption of hyperthermia during intraperitoneal chemotherapy [32, 33], we have shown this treatment is most effective when performed under hyperthermic conditions (intra-abdominal operative temperature of 42.5 °C). Chemotherapy combined with hyperthermia has been proposed to eliminate microscopic disease, thus improving the outcome of CRCPM patients [34], with the added benefit of a direct cytotoxic effect on tumor cells [35]. Indeed, some studies have clarified that hyperthermia can increase drug concentration in intra-abdominal tissues and the rate of systemic absorption [36, 37]. Similarly to in vitro studies showing that hyperthermia increases apoptosis and tumor cell arrest in G1 and G2 phases [38], we have observed an increased rate of apoptotic cells. Overall, our data indicate that PDO models are able to reproduce the results observed in the patient, as also previously reported by Papaccio et al*.* [39], demonstrating the technical feasibility of performing HIPEC treatments with PDOs in a clinically relevant setting.

As for our prospective phase II clinical study, which plans to recruit 25 patients, we have no informative results yet, as at present only two patients undewent CRS/HIPEC, and one of them (treated with MMC) has too short follow-up, while the other (treated with L-OHP) relapsed after 10 months. Of the five patients included in our work who performed CRS/HIPEC, four were treated with MMC (one also in combination with CDDP), and at the last follow-up three of them had no metastases to the peritoneum. The chemosensitivity test on PDOs, although performed after CRS/HIPEC for three patients, confirmed their sensitivity to MMC.

The PDO model, however, lacks the components of the metastatic microenvironment (fibroblasts and macrophages, endothelial cells, immune cells, inflammatory cells, and extracellular matrix), which may support tumor growth and influence the response to therapeutic strategies [40–43]. In particular, the role of CAFs, an important component of the tissue microenvironment (TME), in the initiation of CRC [44–46], its progression, metastatic spread, and the development of a chemotherapy-resistant phenotype has been clearly demonstrated [44–50]. Most importantly, CAFs may contribute to the induction of an immunosuppressive TME [51–54]. Our results confirm that CAFs may influence the response to current therapeutic strategies, suggesting, on the one hand, that an effective *ex-vivo* model for PDO-based therapeutic choice should include components of the TME and, on the other hand, that effective HIPEC treatment should include drugs that also selectively target components of the tumor microenvironment. We are developing advanced co-culture models in which non-epithelial cell types are represented within PDOs, which can represent the biology of their tumor of origin much better than current models.We have already combined PDOs with patient-derived ECM, and showed that presence of the ECM affects treatment response [18]. Further implementation of our model will include ECM components and different subpopulations of the TME to evaluate treatment response. Because PDOs can replicate the stage and interactions within TME of an individual patient, these models represent a good opporunity to be potential platforms for drug screening in translational medicine [18, 55].

## Conclusions

Our results highlight how a PDO-based preclinical model allows for the administration of HIPEC schemes in a biologically relevant environment, as well as the development of new drug combination strategies and in-depth data analyses that will provide targets for tailored therapies. Model implemenation through the additon of TME components that could influence the response to drug treatments, will increase the chance to test HIPEC treatments in a more relevant and realistic environment [56].

### Supplementary Information

**Supplementary Material 1**.

## Data Availability

The data generated and analysed during the current study are available from the corresponding author on reasonable request.

## Abbreviations

3D: Three Dimensional
3D-dECM: Three Dimensional decellularized Extracellular Matrix
ANOVA: Analysis of Variance
CAF: Cancer Associated Fibroblast
CDDP: Cisplatin
CDX2: Homeobox protein CDX-2
CK AE1/AE3: Cytokeratin AE1 and AE3
CK20: Cytokeratin 20
CRC: Colorectal Cancer
CRCPM: Colorectal-derived Peritoneal Metastasis
CRS: Cytoreductive Surgery
CTRL: Control group
DAPI: 4API: Ol group-2-phenylindole
DMEM-F12: Dulbecco's Modified Eagle Medium F12
DMSO: Dimethyl sulfoxide
DOX: Doxorubicin
ECM: Extracellular Matrix
EGF: Epidermal growth factor
EGFR: Epidermal growth factor receptor
ERBB2: Receptor tyrosine-protein kinase erbB-2
FAP: Fibroblast activated protein
FFPE: Formalin-Fixed Paraffin-Embedded
FGF: Fibroblast growth factor
H&E: Hematoxylin and Eosin
HIPEC: Hyperthermic Intraperitoneal Chemotherapy
IC_50_: Concentration required to eliminate 50% of the tumor cells
IF: Immunofluorescence
IGF: Insulin growth factor
IHC: Immunohistochemistry
Ki67: Antigen Kiel 67
L-OHP: Oxaliplatin
MMC: Mitomycin-c
MPP: Metalloproteases
MSCs: Mesenchimal-derived cells
OS: Overall Survival
PBS: Phosphate Buffer Saline
PDO: Patient-derived Organoids
sCT: systemic Chemotherapy
SD: Standard Deviation
α-SMA: Smooth muscle actin alpha
TGFβ: Transforming growth factor beta proprotein
TME: Tumor Microenvironment
UNT: Untreated group
VEGFR: Vascular endothelial growth factor receptor
