# Correction: Colorectal carcinoma peritoneal metastases‑derived organoids: results and perspective of a model for tailoring hyperthermic intraperitoneal chemotherapy from bench‑to‑bedside

**DOI:** 10.1186/s13046-024-03092-x

**Published:** 2024-06-11

**Authors:** Luca Varinelli, Davide Battistessa, Marcello Guaglio, Susanna Zanutto, Oscar Illescas, Ewelina J. Lorenc, Federica Pisati, Shigeki Kusamura, Laura Cattaneo, Giovanna Sabella, Massimo Milione, Alessia Perbellini, Sara Noci, Cinzia Paolino, Elisabetta Kuhn, Margherita Galassi, Tommaso Cavalleri, Marcello Deraco, Manuela Gariboldi, Dario Baratti

**Affiliations:** 1 Department of Experimental Oncology, Molecular Epigenomics Unit, Fondazione IRCCS Istituto Nazionale Tumori, Via G. Venezian 1, Milan, 20133 Italy; 2 Peritoneal Surface Malignancies Unit, Colorectal Surgery, Fondazione IRCCS Istituto Nazionale Tumori, Via G. Venezian 1, Milan, 20133 Italy; 3 Cogentech Ltd. Benefit Corporation With a Sole Shareholder, Via Adamello 16, Milan, 20139 Italy; 4 Pathology and Laboratory Medicine Department, Fondazione IRCCS Istituto Nazionale Dei Tumori Di Milano, Via G. Venezian 1, Milan, 20133 Italy; 5 Department of Biomedical, Surgical and Dental Sciences, University of Milan, Milan, 20122 Italy; 6 Pathology Unit, Foundation IRCCS Ca’ Granda Ospedale Maggiore Policlinico, Milan, 20122 Italy; 7 Centrale Produzione Farmaci, Hospital Pharmacy, Fondazione IRCCS Istituto Nazionale Dei Tumori Di Milano, Via G. Venezian 1, Milan, 20133 Italy

**Correction:** ***J Exp Clin Cancer Res *43, 132 (2024)**

10.1186/s13046-024-03052-5

Following publication of the original article [1], the authors identified an error in the family name of Elisabetta Kuhn.

The incorrect author name is: Elisabetta Khun

The correct author name is: Elisabetta Kuhn

The author group has been updated above and the original article [1] has been corrected.
