# Supplementary material for: Colorectal carcinoma peritoneal metastases-derived organoids: results and perspective of a model for tailoring hyperthermic intraperitoneal chemotherapy from bench-to-bedside
Source: J Exp Clin Cancer Res. 2024 May 2;43:132. doi: 10.1186/s13046-024-03052-5 (PMC11064374; doi:10.1186/s13046-024-03052-5)
Supplement: Supplementary file 1 — Supplementary Material 1. [file 13046_2024_3052_MOESM1_ESM.pdf]

# Supplementary Material and Methods

***Development of CRCPM-derived PDO***

PDOs were seeded in 24-multi well plates and cultured using the 6 different media combinations. The impact of these six media on PDOs growth was determined by counting the number of PDOs present in each well (Supplementary Fig. S2, Supplementary Table S1) using Qupath software (https://qupath.github.io, version 0.2.3). Images used for Qpath analyses were acquired with Aperio Leica ScanScope XT (Leica Biosystems, Wetzlar, Germany). Three different fields were counted for each experiment.

The specific composition of each medium was the following:
- Medium #1: DMEM-F12; B27; Glutamax
- Medium #2: DMEM-F12; B27; Glutamax; N-acetylcysteine; prostaglandin-E2; gastrin-I
- Medium #3: DMEM-F12; B27; Glutamax; N-acetylcysteine; prostaglandin-E2; gastrin-I; A83-01
- Medium #4: DMEM-F12; B27; Glutamax; N-acetylcysteine; prostaglandin-E2; gastrin-I; EGF; A83-01; Noggin
- Medium #5: DMEM-F12; B27; Glutamax; N-acetylcysteine; prostaglandin-E2; gastrin-I; A83-01; SB202190; Noggin
-Medium #6: DMEM-F12; B27; Glutamax; N-acetylcysteine; prostaglandin-E2; gastrin-I; A83-01; Noggin

***Nucleic acids extraction and Mutational analysis***

DNA was extracted from PDOs (PM1, PM2, PM3, PM4, PM5, PM6 and PM7) and from formalin-fixed paraffin-embedded (FFPE) sections of the tumors from which they were derived using the DNeasy Blood&Tissue kit (QIAGEN, Germany) and the Masterpure Complete DNA Purification Kit (Lucigen-Biosearch Technologies, Middleton, WI, USA) respectively. DNA was quantified with Nanodrop™ 1000 (ThermoFisher Scientific, USA) and Qubit 2.0 flurorimeter (Thermofisher Scientific, USA) and used for mutational analysis with the 50-gene Ion AmpliSeq Cancer Hotspot Panel v2 (Life Technologies, USA). The mutational status of a group genes was assessed using targeted next-generation sequencing (T-NGS), with the Ion-Torrent™ Personal Genome Machine platform (Life Technologies, USA, genes analyzed: *ABL1, AKT, ALK, APC, ATM, BRAF, CDH1, CDKN2A, CSF1R, CTNB1, EGFR, ERBB2, ERBB4, EZH2, FBXW7, FGFR1, FGFR2, FGFR3, FLT3, GNA11, GNAQ, GNAS, HNF1A, HRAS, IDH1, IDH2, JAK2, JAK3, KDR (VEGFR2), KIT, KRAS, MET, MLH1, MPL, NOTCH1, NPM1, NRAS, PDGFRA, PIK3CA, PTEN, PTPN11, RB1, RET, SMAD4, SMARCB1, SMO, SRC, STK11, TP53* and *VHL*).
The Ion AmpliSeq Library Kit 2.0 (Life Technologies) was used to amplify 40 ng of DNA according to the manufacturer's instructions (MAN0006735 rev 5.0). Libraries were purified using AMPure Beads XP (Beckman Coulter) and PCR-amplified for a total of five cycles. Emulsion PCR and sample enrichment were completed using the IonOne Touch 2 instrument. Sequencing was done on Ion Torrent PGM using Ion 316 Chips and the Ion-PGM 200 sequencing kit (Life Technologies), according to the manufacturer's instructions.
PGM sequencing data were initially processed using the Ion Torrent platform-specific Torrent Suite software to generate sequence readouts, align them on the reference genome Hg19, trim the adapter sequences, filter and discard any poor signal-profile readouts. Variant calling from the sequencing data was done with the Variant Caller plug-in. The filtered variants were examined visually using the Integrative Genomic Viewer tool to check their quality level and confirm the variant's presence on both the "+" and the "-" strand. The resulting variants were then recorded using the Ensemble Variant Effect Predictor pipeline, COSMIC database, dbSNP database and MyCancerGenome database (http://www.mycancergenome.org/).

# Supplementary Material and Methods

***Immunohistochemistry analyses***

FFPE blocks were prepared for immunohistochemistry (IHC) as previously described [1]. PDOs were included in paraffin and cut into slices (3 µm); paraffin was removed with xylene and slices were rehydrated using decreasing concentrations of ethanol, and washed with water. IHC was performed with the following antibodies: CK AE1/AE3, CK20, CDX2 and ki-67, following the specifications reported in Supplementary Table S6. Images were acquired with a DM6000B microscope (Leica). Antigen retrieval was carried out using preheated target retrieval solution (pH 6.0) for 30 minutes. Tissue sections were blocked with FBS serum in PBS for 60 min and incubated overnight with primary antibody. The antibody binding was detected using a polymer detection kit (GAM/GAR-HRP, Microtech, Italy) followed by a diaminobenzidine chromogen reaction (Peroxidase substrate kit, DAB, SK-4100; Vector Lab, USA). All sections were counterstained with Mayer's hematoxylin.

***QuPath analyses***

Cell counting and percentage estimation of cCASPASE3-positive cells were performed using Qupath software (https://qupath.github.io, version 0.2.3). The percentage of positive cells was calculated by dividing the number of positive cells present in each field by the total number of cells (cCASPASE$^{+}$ + cCASPASE3$^{-}$) in the same field.

***CellTiterGlo® 3D Cell Viability Assay and Caspase-Glo® 3 Assay protocol***

PDOs and co-culture viability and CASPASE 3 activation were assessed using a CellTiterGlo® 3D Cell Viability Assay kit and a Caspase-Glo® 3 assay, respectively (Promega, USA) on a TECAN spark microplate reader (Tecan Trading AG, Switzerland) following the manufacturers' instructions. Data were normalized to the mean of the untreated group (UNT: PDO treated with DMEM-F12 only). All the experiments were performed in triplicate.

***Immunofluorescence analyses on co-culture models***

Immunofluorescence analyses on co-cultures were performed as previously reported [2-3]. CAFs were seeded in an 8-wells chambered coverslip (Ibidi) as described above. Co-cultures were directly fixed in plate with 4 % paraformaldehyde (Sigma-Adrich) for 15 min at 4 °C, then the plate was swirled and the co-cultures were incubated in 4 % paraformaldehyde for other 15 min at 4 °C. Co-cultures were permeabilized with 0.5 % Triton X-100 (Sigma-Aldrich) for 30 min at RT on a rotor (100 rpm) and blocked for one hour with 5 % BSA and 0.1 % Triton X-100 in PBS 1X at RT on a rotor (100 rpm). Primary antibody mouse anti α-Smooth Muscle Actin (α-SMA) conjugated with Alexaflour 488 dye (1:500, Cell Signalling Thecnology, clone 1A4, #46489) and rabbit anti anti-pan cytokeratin (1:100, Abcam, clone C-11, ab7753) were used overnight at 4 °C and detected using secondary goat anti rabbit AlexaFluor 568 (1:500, Thermo Fisher, A11036) for one hour at RT. Nuclei were stained with DAPI (1:25000, Thermo Fisher, D1306). Images were acquired on DM600B microscope (Leica, Germany) equipped with a 100 W mercury lamp and analyzed using Cytovision software (Leica, Germany).

***Immunoblotting: Cancer –associated fibroblast characterization***

CAF were lysed and their protein content was extracted as in [1]. For each sample, 40 µg of protein extract were separated on 4–12% polyacrylamide gels and incubated with the following primary antibodies: α-SMA; FAP; E-Cadherin and Vinculin. The signals were detected using enhanced chemiluminescence, and protein levels were quantified using Image Lab Software (Bio-Rad, Hercules, CA, USA) (Supplementary Table S3).

***References***


1. Varinelli L, Guaglio M, Brich S, Zanutto S, Belfiore A, Zanardi F, et al. Decellularized Normal and Tumor Extracellular Matrix as Scaffold for Cancer Organoid Cultures of Colorectal Peritoneal Metastases. J Moll Cell Biol. 2023;6;14(11):mjac064.
2. Bergdorf KN, Phifer CJ, Bechard ME, Lee MA, McDonald OG, Lee E, et al. Immunofluorescent staining of cancer spheroids and fine-needle aspiration-derived organoids. STAR Protoc. 2021;18,2(2):100578.
3. Kodba S and Chaigne A. A quick, cheap, and reliable protocol for immunofluorescence of pluripotent and differentiating mouse embryonic stem cells in 2D and 3D colonies. STAR Protoc. 2023;17;4(1):102000.

# Supplementary informations

## Supplementary Table S1

| Factor | Description | Vendor | Working Concentration |
|---|---|---|---|
| Gentamicin | Antibiotic | ThermoFisher Scientific | 50 ng/ml |
| HEPES | Buffer | ThermoFisher Scientific | 10 mM |
| L-Glutamine (GlutaMAX) | Cell culture supplement | ThermoFisher Scientific | 2 mM |
| B27 | Cell culture supplement | ThermoFisher Scientific | 1:50 |
| Gastrin-1, recombinant human | Recombinant protein | Sigma Aldrich | 10 nM |
| N-acetylcysteine | Colonic niche factor | Wako | 1 mM |
| EGF, recombinant human | Recombinant protein | ThermoFisher Scientific | 50 ng/ml |
| Noggin, recombinant human | Recombinant protein | Preprotech | 100 ng/ml |
| Prostaglandin E2 | Colonic niche factor | Tocris | 100 nM |
| A83-01 | p38 inhibitor | Tocris | 500 nM |
| SB202190 | ROCK inhibitor | Sigma Aldrich | 10 µM |

**Supplementary Table S1:** The complete list of growth factors and media supplements, with their working concentrations used for TDOs culturing.

# Supplementary information

## Supplementary Table S2

| PDO culture | Medium composition |
|---|---|
| **C1** | DMEM-F12; B27; Glutamax; N-acetylcysteine; prostaglandin-E2; gastrin-I |
| **C2** | DMEM-F12; B27; Glutamax |
| **C3** | DMEM-F12; B27; Glutamax; N-acetylcysteine; prostaglandin-E2; gastrin-I; A83-01 |
| **C4** | DMEM-F12; B27; Glutamax; N-acetylcysteine; prostaglandin-E2; gastrin-I; EGF; A83-01; Noggin |
| **C6** | DMEM-F12; B27; Glutamax; N-acetylcysteine; prostaglandin-E2; gastrin-I; A83-01; Noggin |
| **PM1** | DMEM-F12; B27; Glutamax; N-acetylcysteine; prostaglandin-E2; gastrin-I |
| **PM2** | DMEM-F12; B27; Glutamax; N-acetylcysteine; prostaglandin-E2; gastrin-I |
| **PM3** | DMEM-F12; B27; Glutamax; N-acetylcysteine; prostaglandin-E2; gastrin-I; A83-01; SB202190; Noggin |
| **PM4** | DMEM-F12; B27; Glutamax; N-acetylcysteine; prostaglandin-E2; gastrin-I; A83-01; SB202190; Noggin |
| **PM5** | DMEM-F12; B27; Glutamax; N-acetylcysteine; prostaglandin-E2; gastrin-I |
| **PM6** | DMEM-F12; B27; Glutamax |
| **PM7** | DMEM-F12; B27; Glutamax; N-acetylcysteine; prostaglandin-E2; gastrin-I; A83-01; SB202190; Noggin |

**Supplementary Table S2**: The specific media formulation for C1, C2, C3, C4, C5, C6 PM1, PM2, PM3, PM4, PM5, PM6 and PM7 PDO cultures.

## Supplementary Table S3

| Antigen (human) | Host | Vendor | Dilution | Incubation |
|---|---|---|---|---|
| **α-SMA** | Mouse | Cell Signaling Technology | 1:1000 | O/N at 4 °C |
| **FAP** | Rabbit | Cell Signaling Technology | 1:1000 | O/N at 4 °C |
| **E-Cadherin** | Mouse | Cell Signaling Technology | 1:1000 | O/N at 4 °C |
| **Vinculin** | Mouse | Cell Signaling Technology | 1:1000 | O/N at 4 °C |

**Supplementary Table S3:** The primary antibodies and the experimental conditions used for WB analyses of CAFs.

# Supplementary table S4: Determination of the clinically relevant doses

| Employed Drugs | HIPEC Clinical Scheme | Normalized Concentration (mg/L)* | mol (g/MW) | Clinically relevant dose [M]** |
|---|---|---|---|---|
| 1<br>(MMC) | 35 mg/m$^2$ MMC in 2.5 L/m$^2$ perfusate | 35mg/m$^2$ / 2.5L/m$^2$ = 14mg/L | 0.014 g/334.33 g/mol = 4.19 x 10$^{-5}$ mol | 4.19 x 10$^{-5}$ mol / 1L = 41.9 µM |
| 2<br>(MMC + CDDP) | 3.5mg/m$^2$/L MMC + 25 mg/m$^2$/L CDDP in perfusate | 3.5mg/m$^2$ / 1L/m$^2$ =<br>3.5mg/L MMC<br>25mg/m$^2$ / 1L/m$^2$ =<br>25mg/L CDDP | 0.0035g / 334.33g/mol =<br>1.04 x 10$^{-5}$ mol (MMC)<br>0.025g / 301.11g/mol =<br>8.30 x 10$^{-5}$ mol (CDDP) | 1.04 x 10$^{-5}$ / 1L =<br>10.4 µM MMC<br>8.30 x 10$^{-5}$ mol / 1L =<br>83 µM CDDP |
| 3<br>(DOX + CDDP) | 15.25 mg/L DOX + 43 mg/L CDDP in perfusate | 15.25mg/L / 1L =<br>15.25mg/L (DOX)<br>43mg/L / 1L = 43<br>mg/L (CDDP) | 0.015250g / 543.52g/mol<br>= 2.81 x 10$^{-5}$ mol (DOX)<br>0.043g / 301.11 g/mol =<br>1.43 x 10$^{-4}$ mol (CDDP) | 2.81 x 10$^{-5}$ mol / 5L =<br>5.61 µM DOX<br>1.43 x 10$^{-4}$ mol / 5L =<br>28.6 µM CDDP |
| 4<br>(L-OHP$_{Low\text{-}dose}$) | 200 mg/m$^2$ in 2 L 5 % Glucosate | 200mg/m$^2$ / 2L/m$^2$ = 100mg/L | 0.100g / 397.28g/mol = 5.03 x 10$^{-4}$ mol | 5.03 x 10$^{-4}$ mol / 2L = 252 µM |
| 5<br>(L-OHP$_{High\text{-}dose}$) | 460 mg/m$^2$ in 2 L 5 % Glucosate | 460mg/m$^2$ / 2L/m$^2$ = 230mg/L | 0.230g / 397.28g/mol = 1.16 x 10$^{-3}$ mol | 1.16 x 10$^{-3}$ mol / 2L= 579 µM |

**Supplementary table S4:** Determination of the clinically relevant dose for each HIPEC scheme tested. * Concentrations were obtained by normalizing each concentrations using 1 L as the final reference dilution volume. ** Clinically relevant doses were obtained using 1 L (scheme 1 and 2), 5 L (scheme 3) and 2 L (scheme 4 and 5) as the final reference dilution volumes.

# Supplementary table S5:  NGS analysis (PDOs Vs Tissues)

| ID | Mutation in PDO | Mutation in Tissue |
|---|---|---|
| C1 [18] | *KRAS*; p.G12S<br>*TP53*; p.R175H<br>*RAD51C*; p.D253H | *KRAS*; p.G12S<br>*TP53*; p.R175H |
| C2 [18] | *BRAF*; p.V600E<br>*TP53*; p.P152L<br>*BARD*; p.A40V | *BRAF*; p.V600E<br>*TP53*; p.P152L |
| C3 [18] | KRAS; p.G12S<br>TP53; p.R273C<br>*FGR1*; amp | *KRAS*; p.G12S<br>*TP53*; p.R273C<br>*FGR1*; amp |
| C4 [18] | *KRAS*; p.G12S<br>*BARD*; p.V507M<br>*MSH3*; p.A55A60del<br>*MSH6*; p.R62R | *KRAS*; p.G12S |
| C6 [18] | *KRAS*; p.G12S<br>*TP53*; p.G245V<br>*APC*; p.Q1406Fs<br>*SMAD4*; pD315H | *KRAS*; p.G12S<br>*TP53*; p.G245V<br>*APC*; p.Q1406Fs<br>*SMAD4*; pD315H |
| PM1 | *APC*; p.E1309KFsTer5<br>*KRAS*; p.G12D<br>*TP53*; p.R248W | *APC*; p.E1309KFsTer5<br>*KRAS*; p.G12D<br>*TP53*; p.R248W |
| PM2 | *APC*; p.R1450$^{*}$<br>*KRAS*; p.Q61H<br>*TP53*; p.R2486Q | *APC*; p.R1450$^{*}$<br>*KRAS*; p.Q61H<br>*TP53*; p.R2486Q |
| PM3 | *KRAS*; p.G12C | *KRAS*; p.G12C |
| PM4 | *KRAS*; p.Q61H<br>*TP53*; p.R175H | *KRAS*; p.Q61H<br>*TP53*; p.R175H |
| PM5 | *KRAS*; p.G12S | *KRAS*; p.G12S |
| PM6 | *BRAF*; p.V600E<br>*TP53*; p.P152L | *BRAF*; p.V600E<br>*TP53*; p.P152L<br>*SMAD4*; pL146$^{*}$<br>*KDR*; p.R962C |
| PM7 | *APC*; p.M1383dup<br>*TP53*; p.R248Q | *APC*; p.M1383dup<br>*TP53*; p.R248Q |

**Supplementary Table S5:** The mutational profile of PDOs and their corresponding tissue.

Amp: amplified; del: deleted; dup: duplication; Fs: frameshift mutation; FsTer5: frameshift muation tahta generates a stop codon; $^{*}$ stop codon.

# Supplementary Table S6

| Antigen (human) | Host | Clone | Vendor | Dilution | Antigen retrieval solution |
|---|---|---|---|---|---|
| **Ki-67** | Mouse | MIB-1 | Dako | 1:400 | 5 mM EDTA (pH 8), 10 min, 96 °C |
| **CK20** | Mouse | Ks20.8 | Dako | 1:500 | 5 mM EDTA (pH 8), 30 min, 96°C |
| **CK AE1/AE3** | Mouse | AE1+AE3 | Dako | 1:100 | 10 mM Citrate (pH 6), 15 min, 96°C |
| **CDX2** | Mouse | CDX2_88 | Dako | 1:50 | 5 mM EDTA (pH 8), 30 min, 96°C |

**Supplementary Table S6:** Primary antibodies and experimental conditions used for IHC analyses.

# Supplementary Figure S1

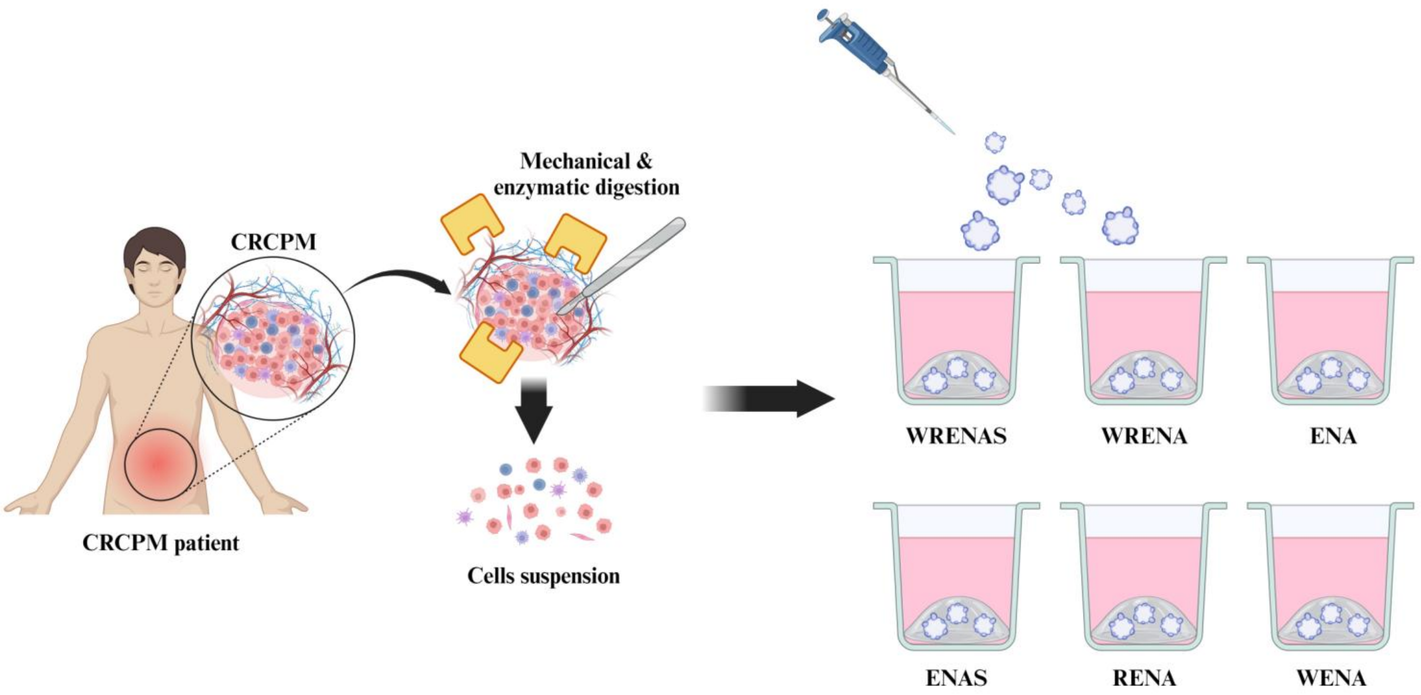


| Entry | Entry name | Description |
|---|---|---|
| **W** | Wnt3A | Recombinant protein |
| **R** | R-spondin-1 | Recombinant protein |
| **E** | EGF | Recombinant protein |
| **N** | Noggin | Recombinant protein |
| **A** | A83-01 | Alk inhibitor |
| **S** | SB202190 | Anti-p38 inhibitor |

## Supplementary Figure S2

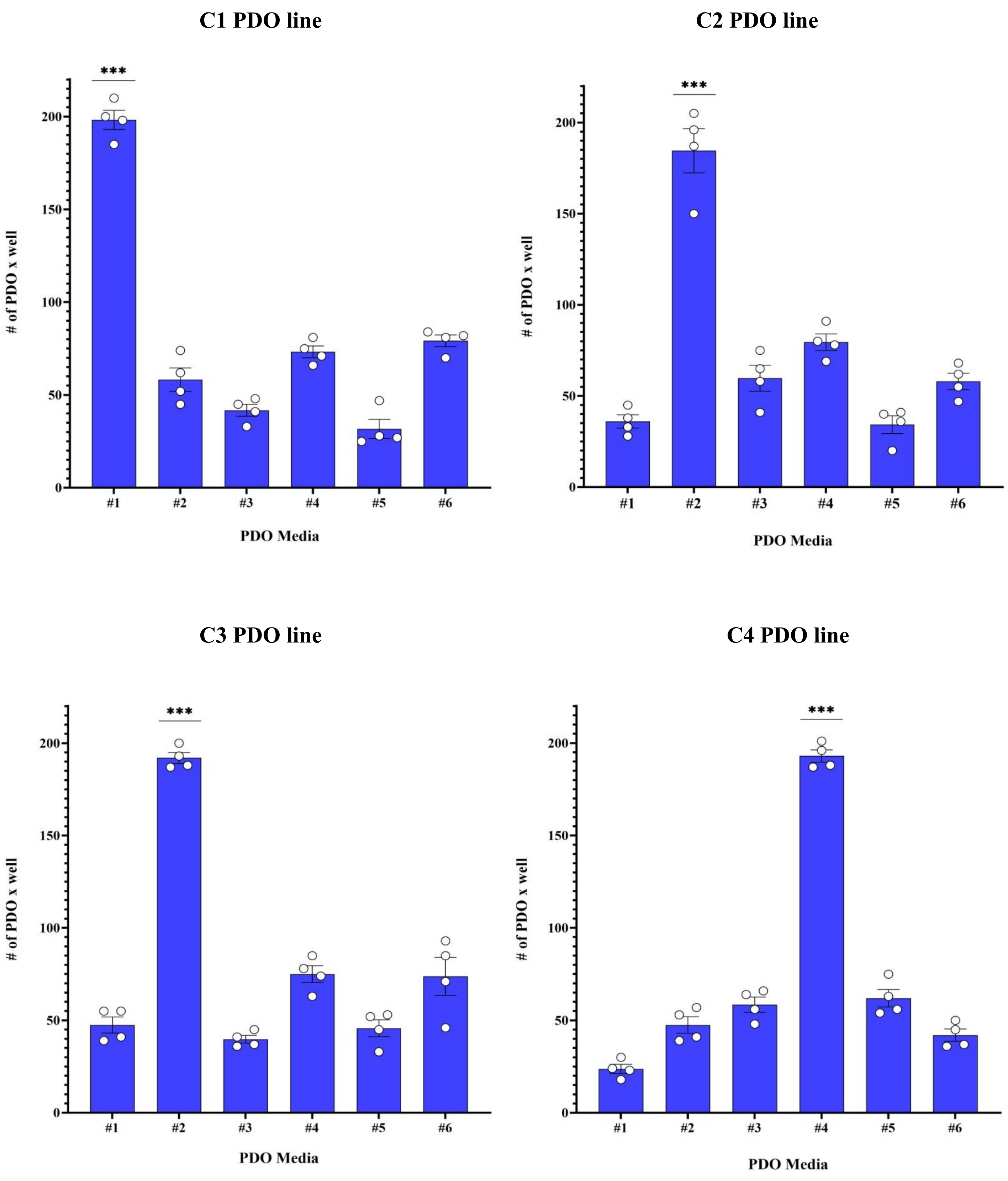

# Supplementary Figure S2

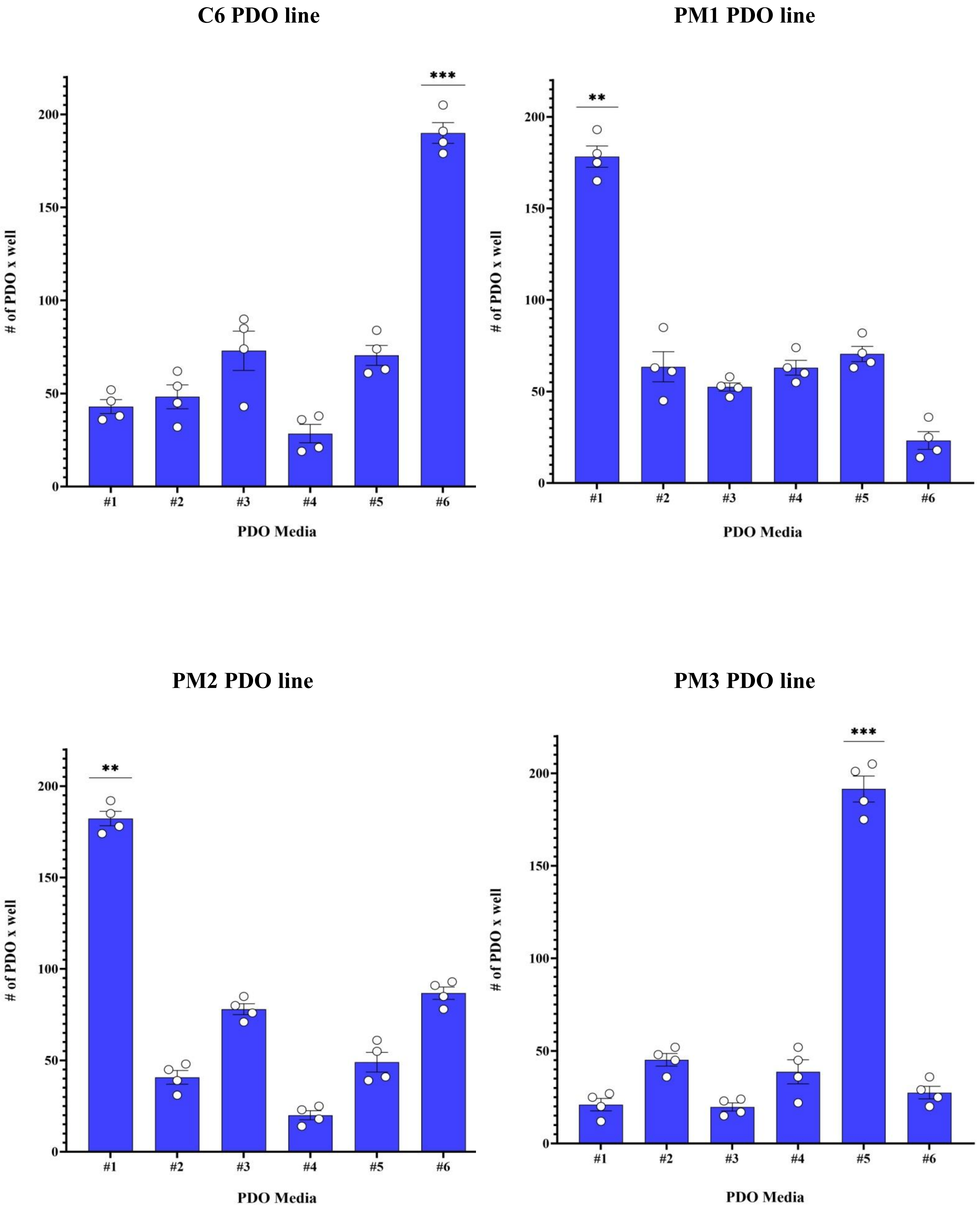

# Supplementary Figure S2

## PM4 PDO line

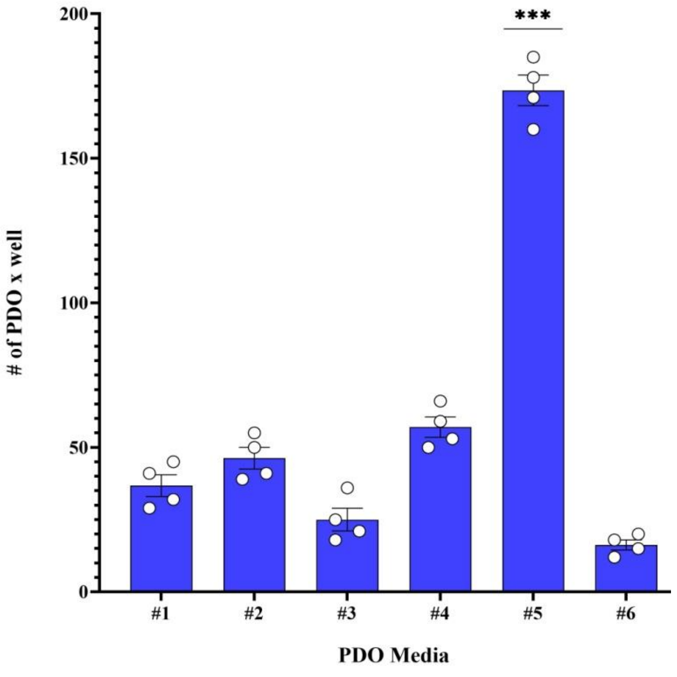


## PM5 PDO line

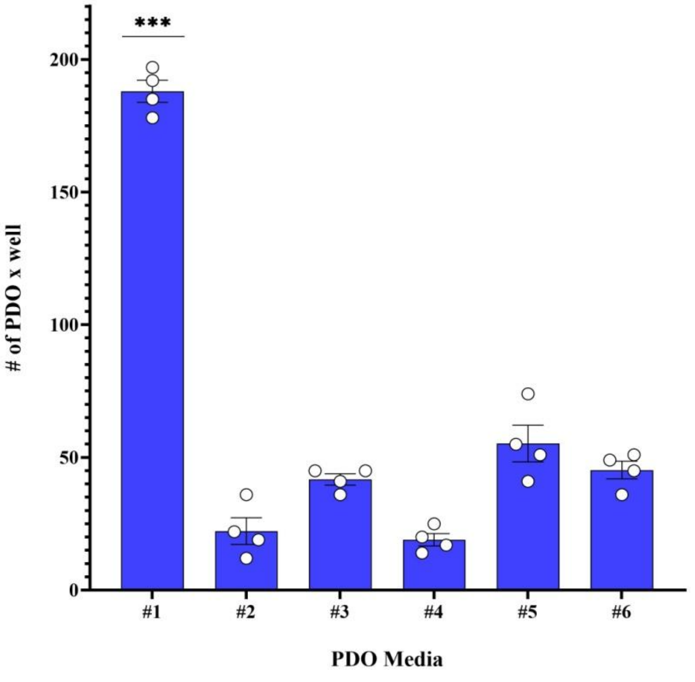


## PM6 PDO line

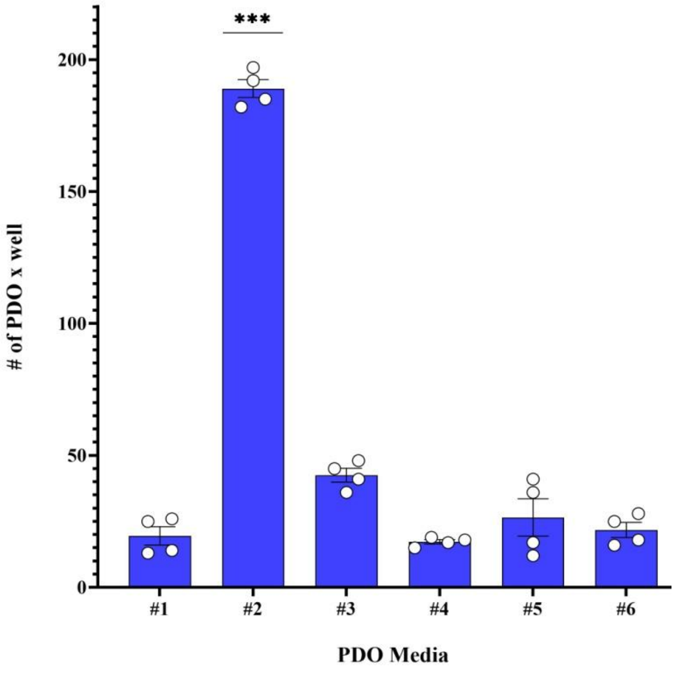


## PM7 PDO line

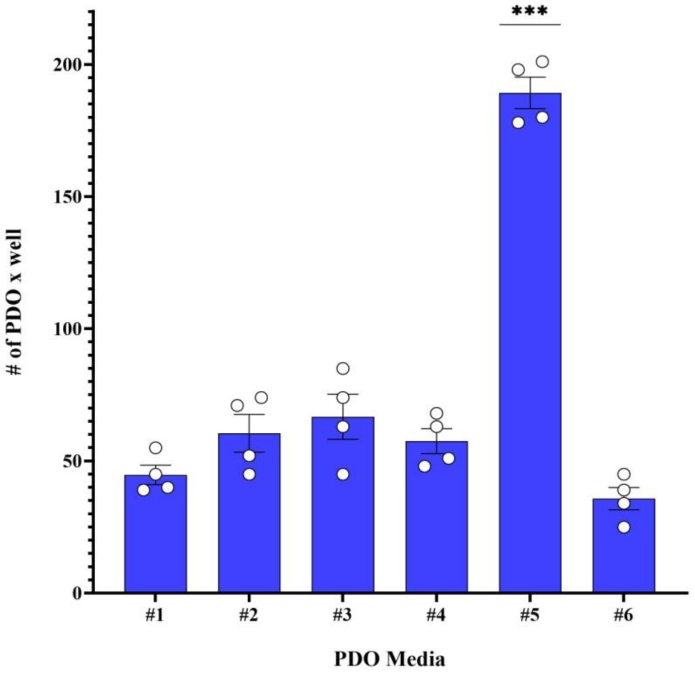

## Supplementary Figure S3

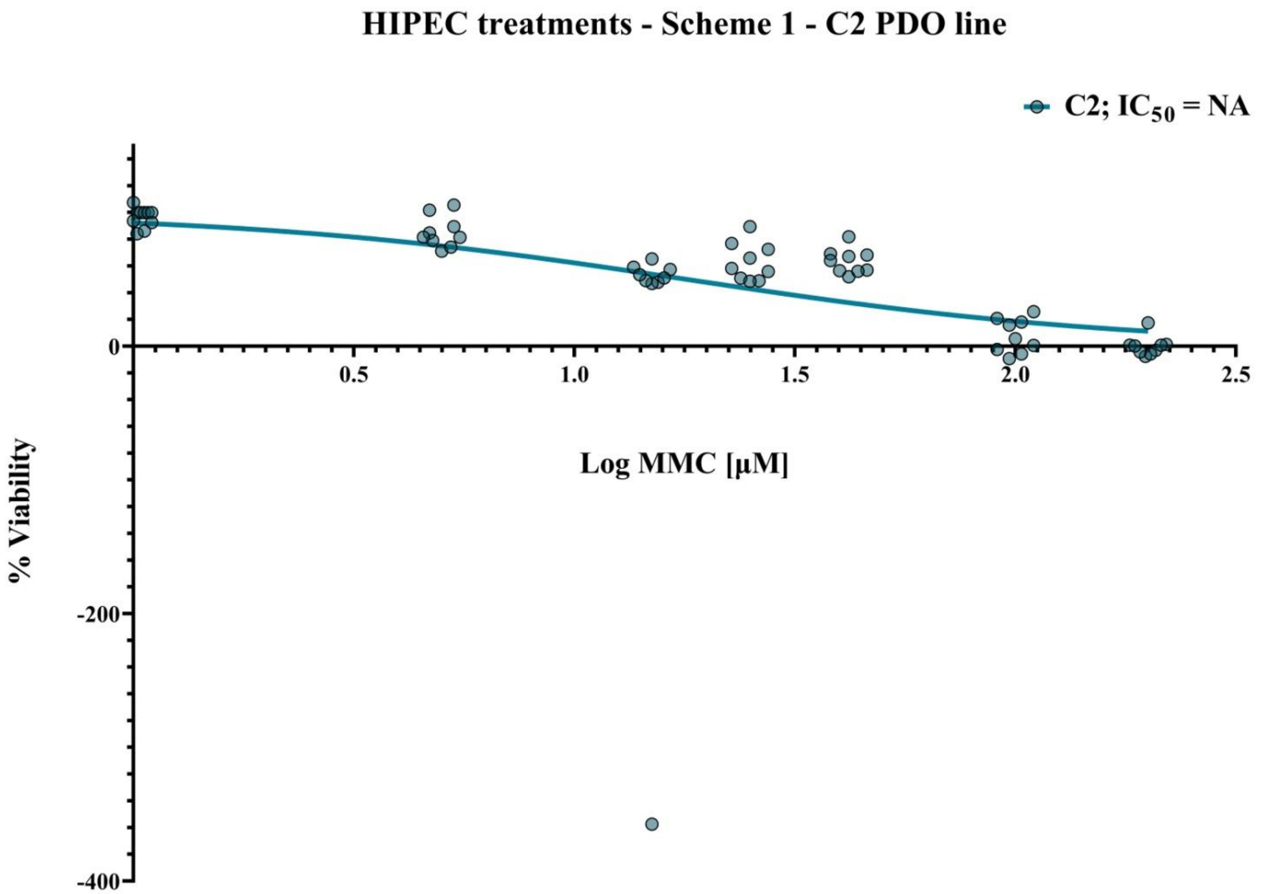


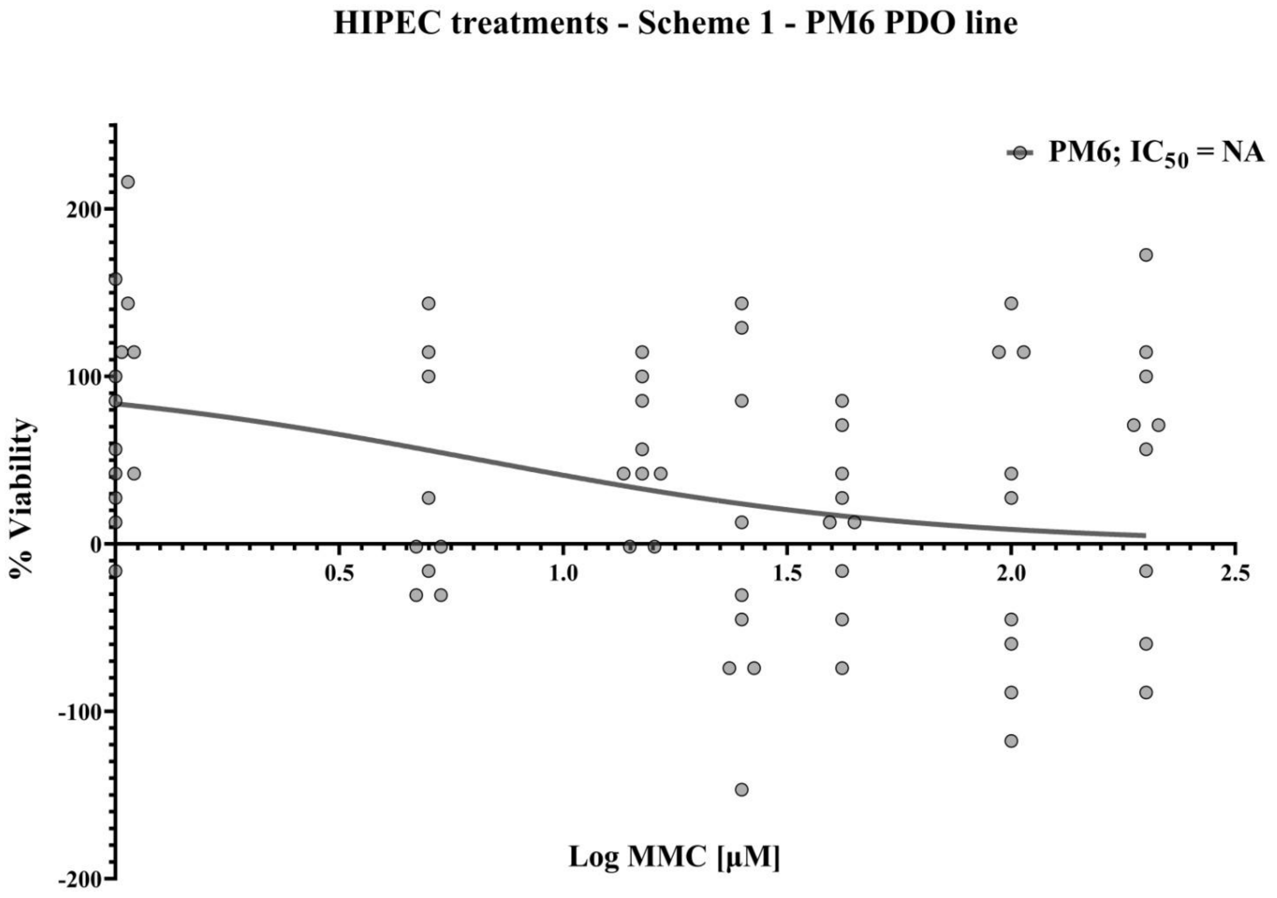

## Supplementary Figure S3

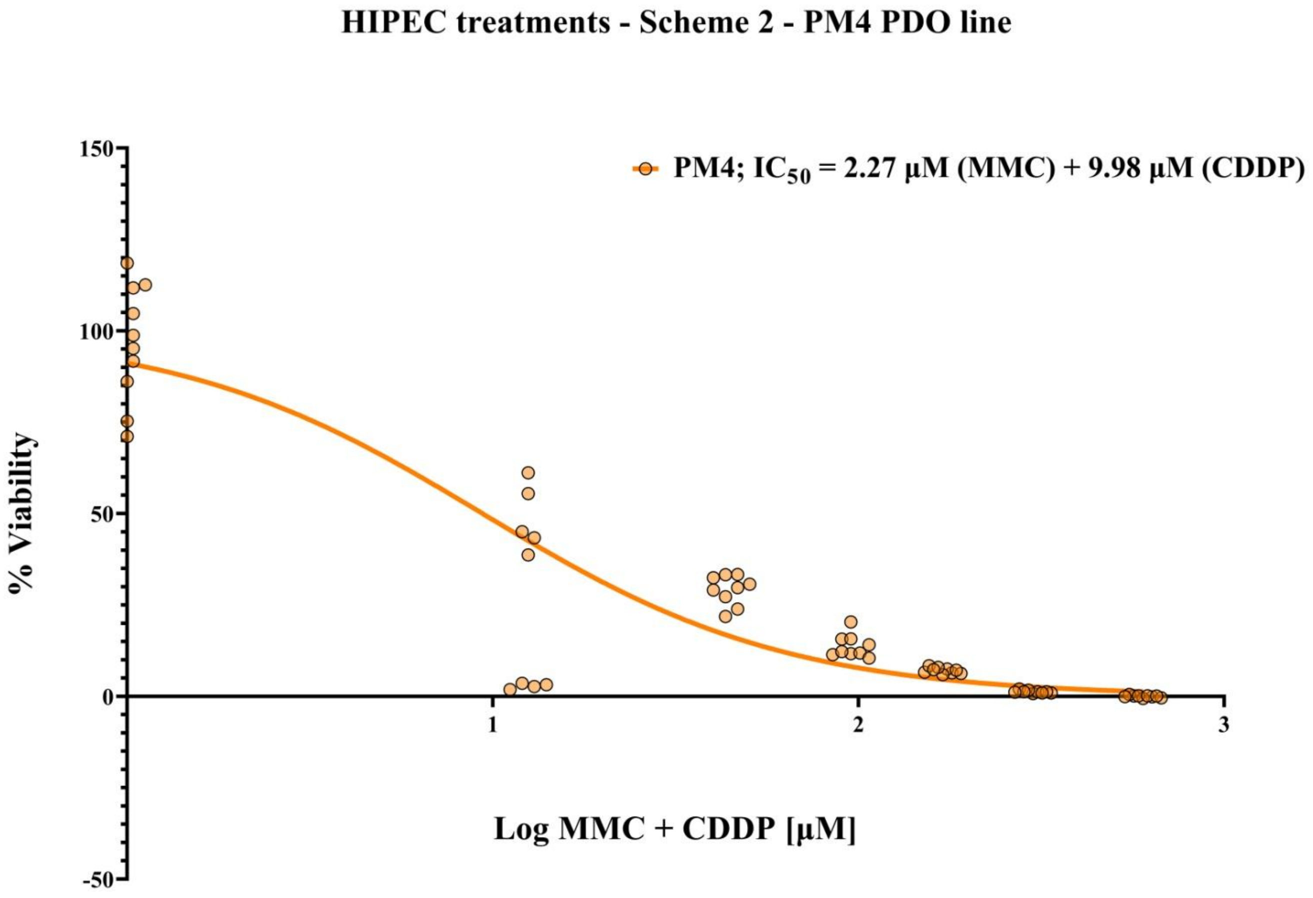


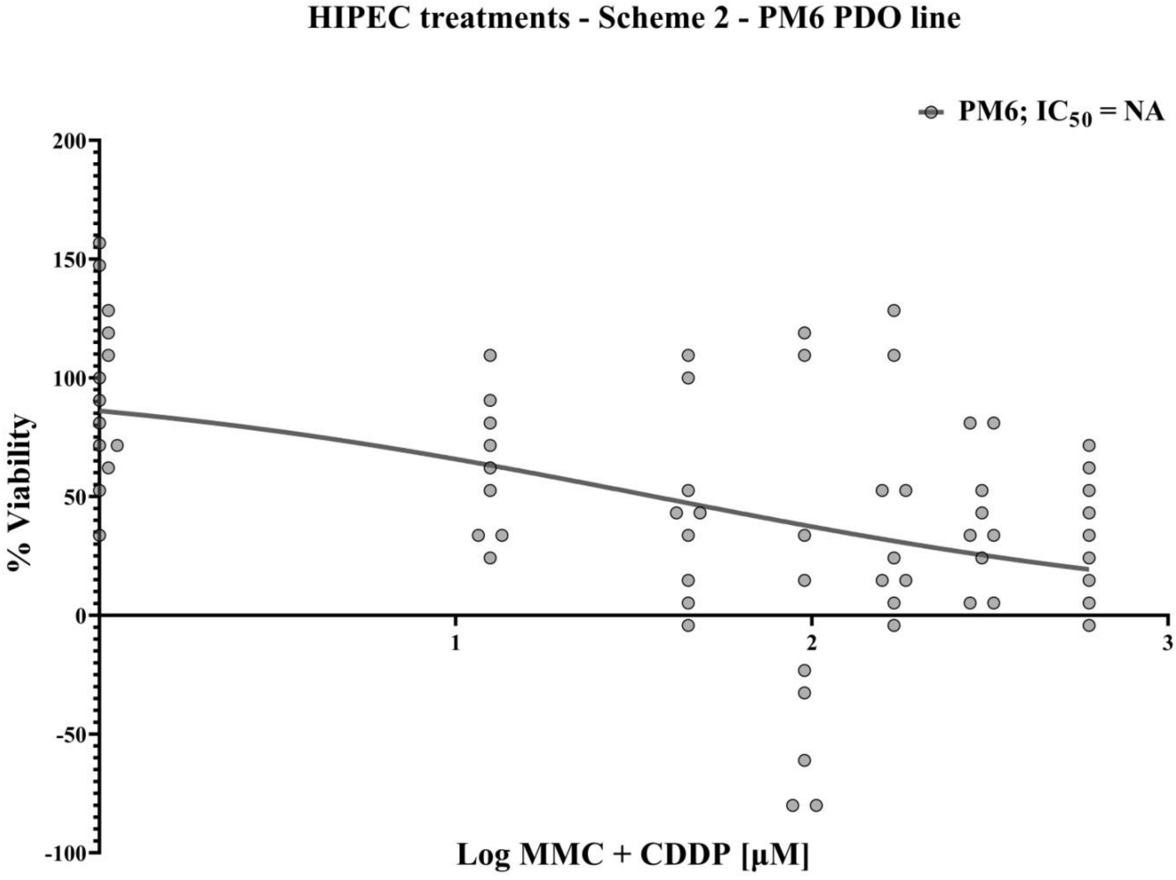

**Supplementary Figure S3**

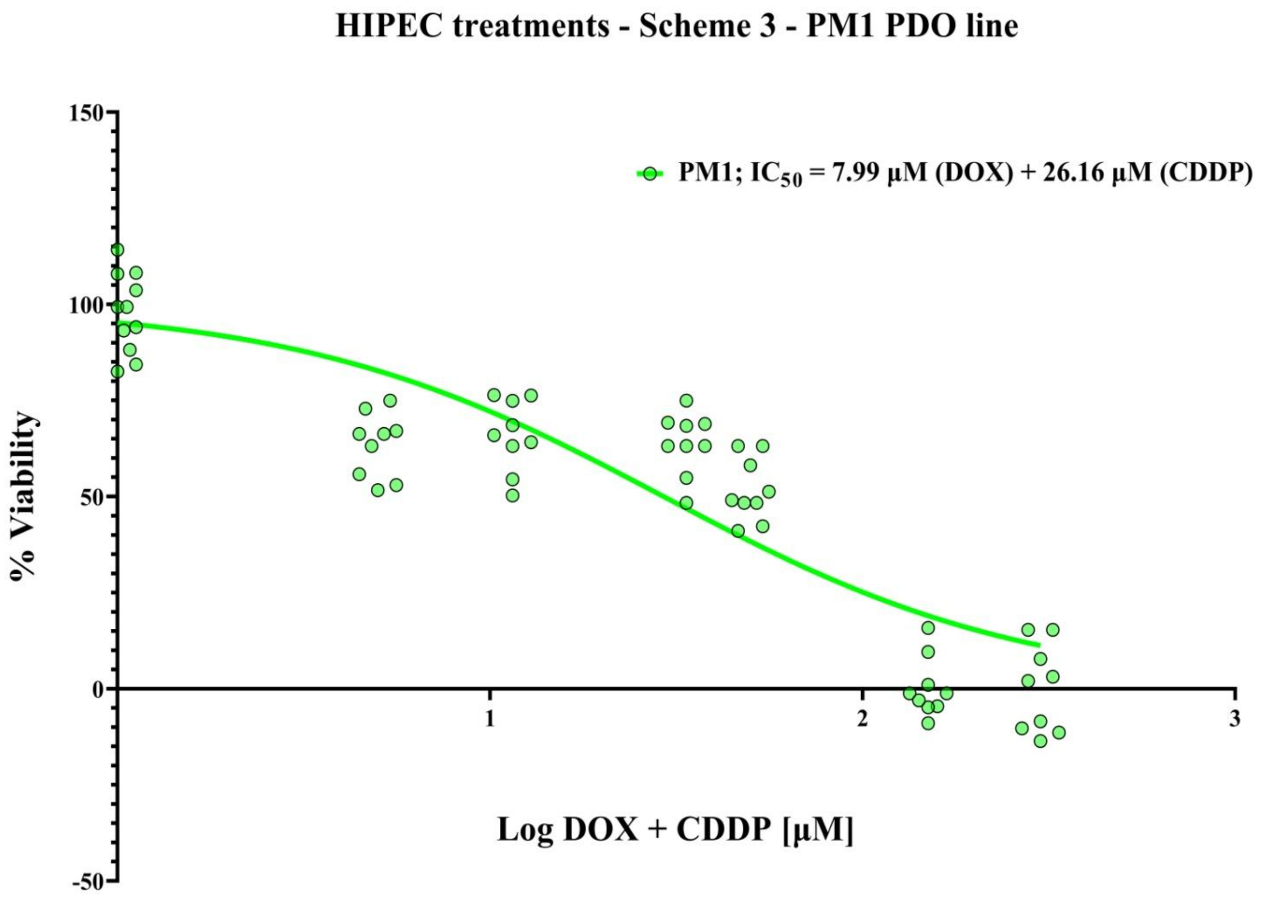


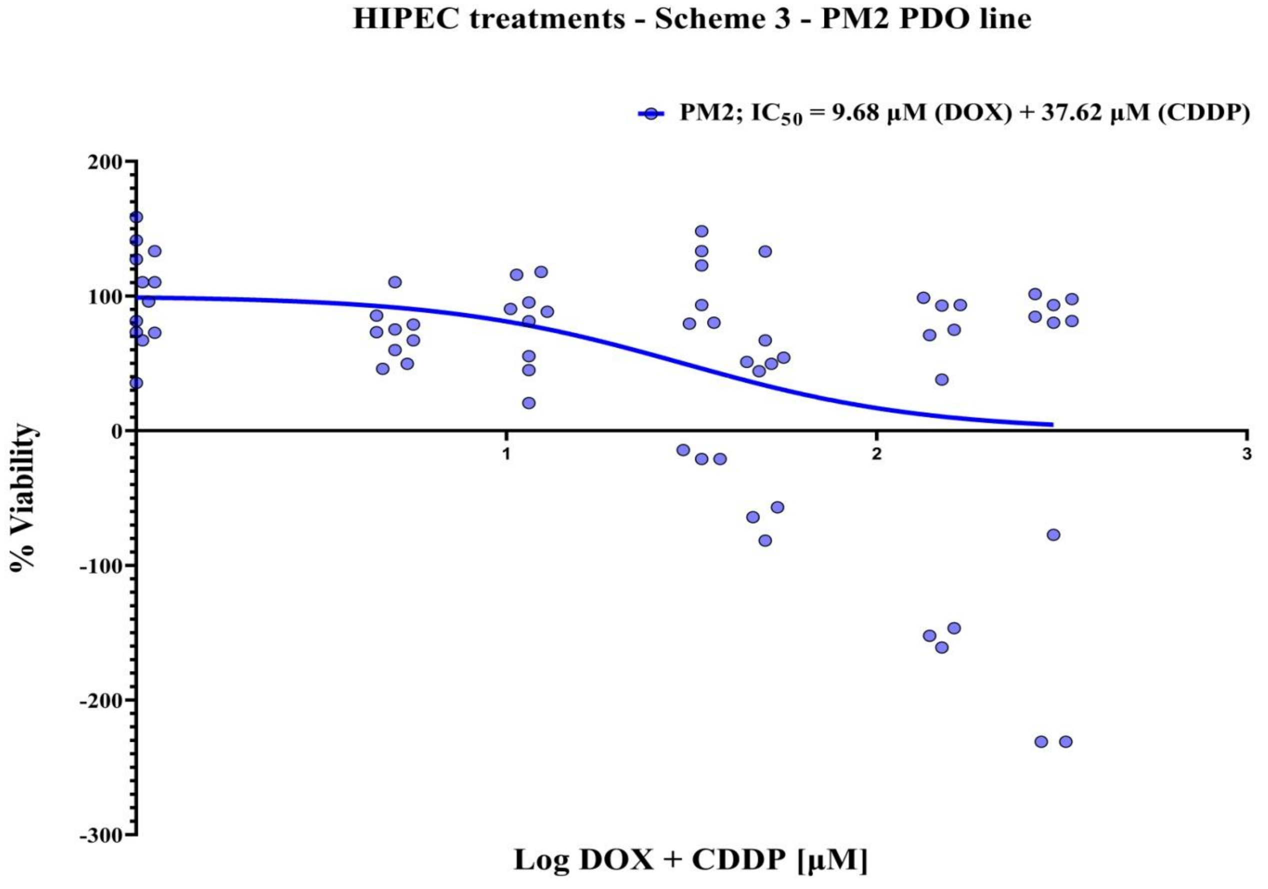

# Supplementary Figure S3

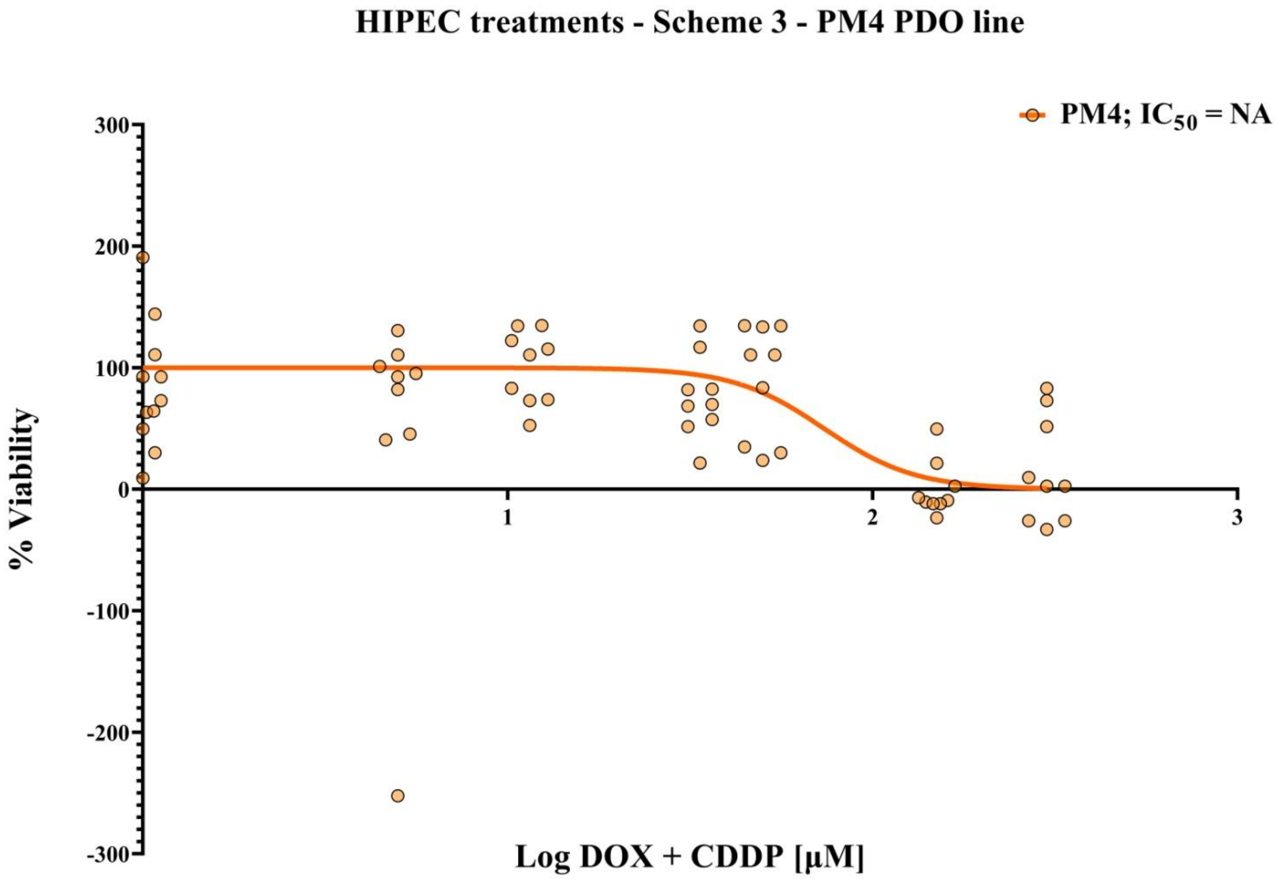


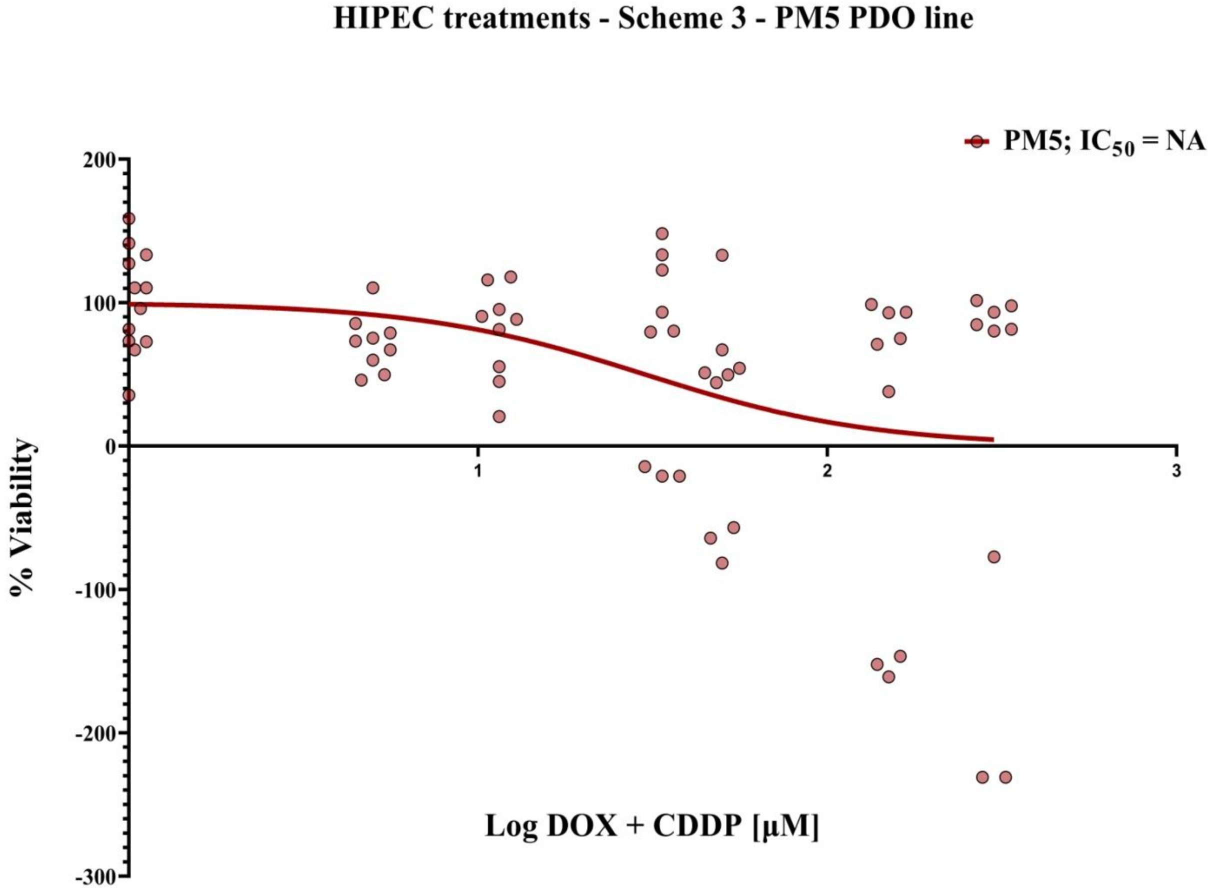

**Supplementary Figure S3**

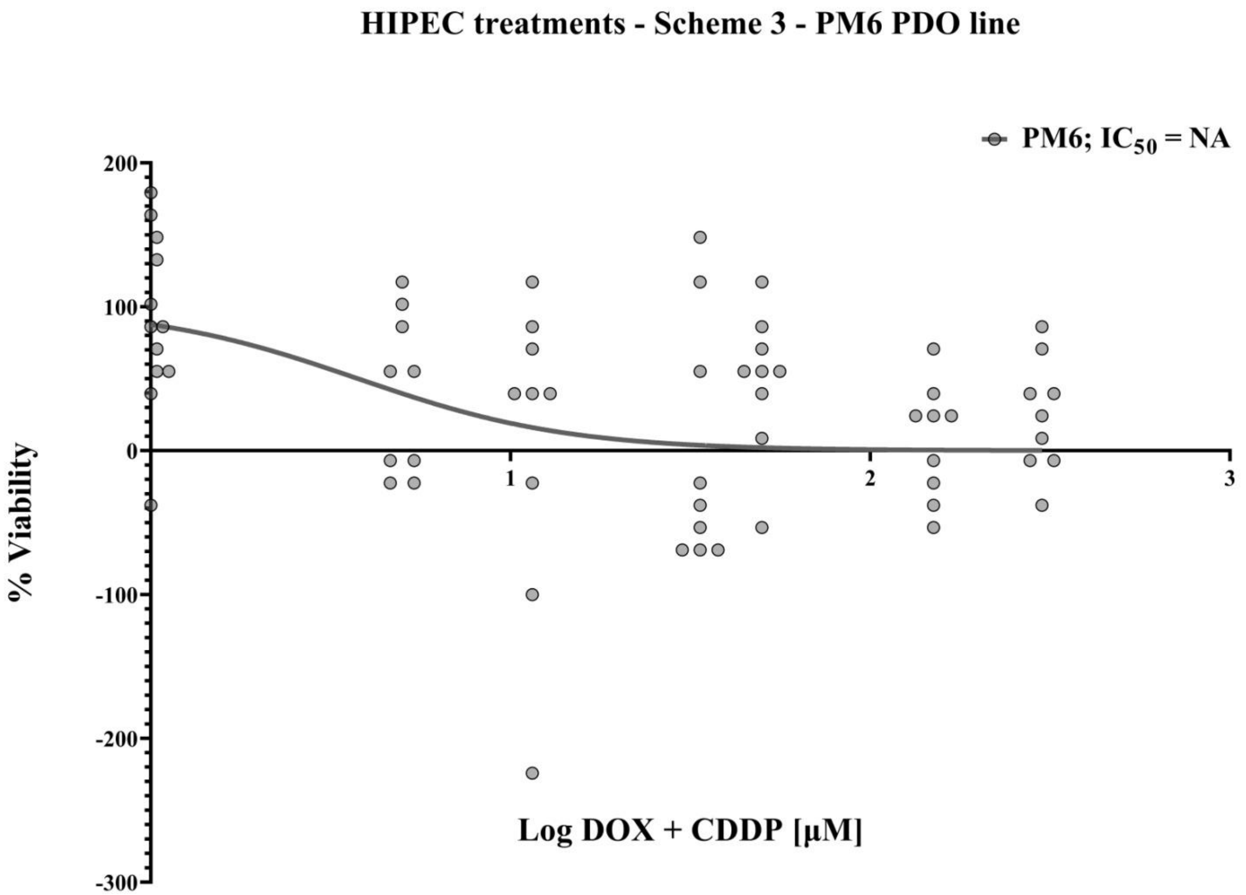


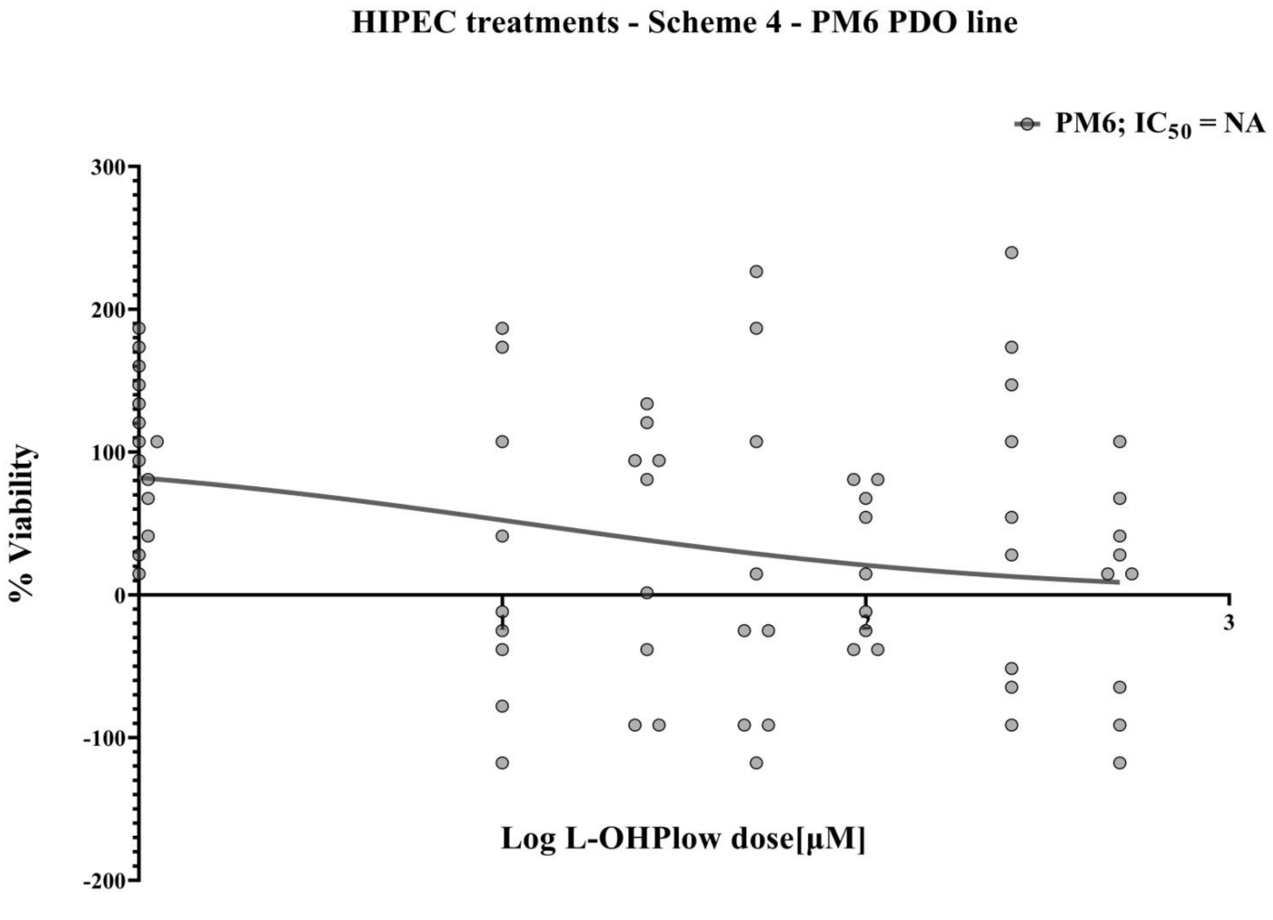

# Supplementary Figure S3

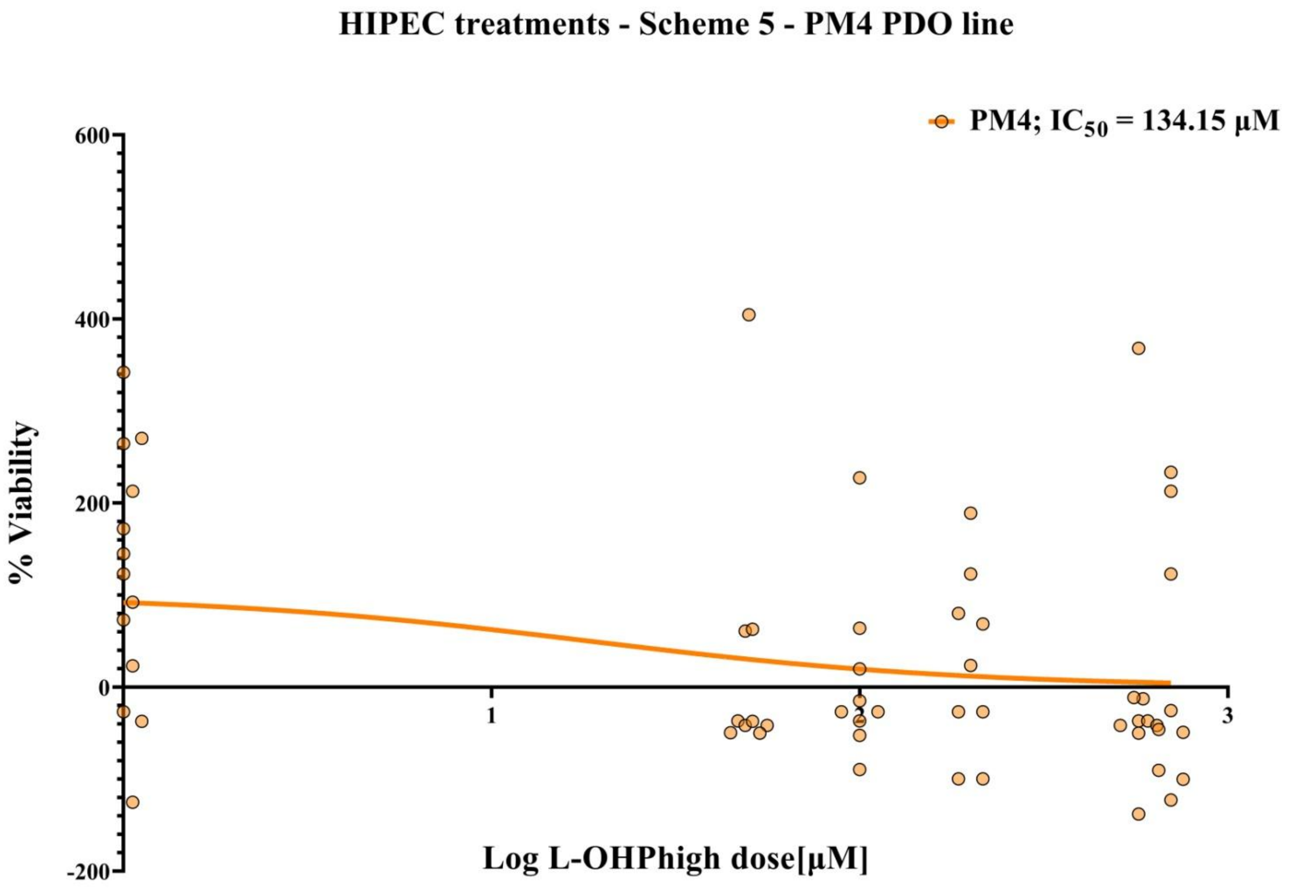


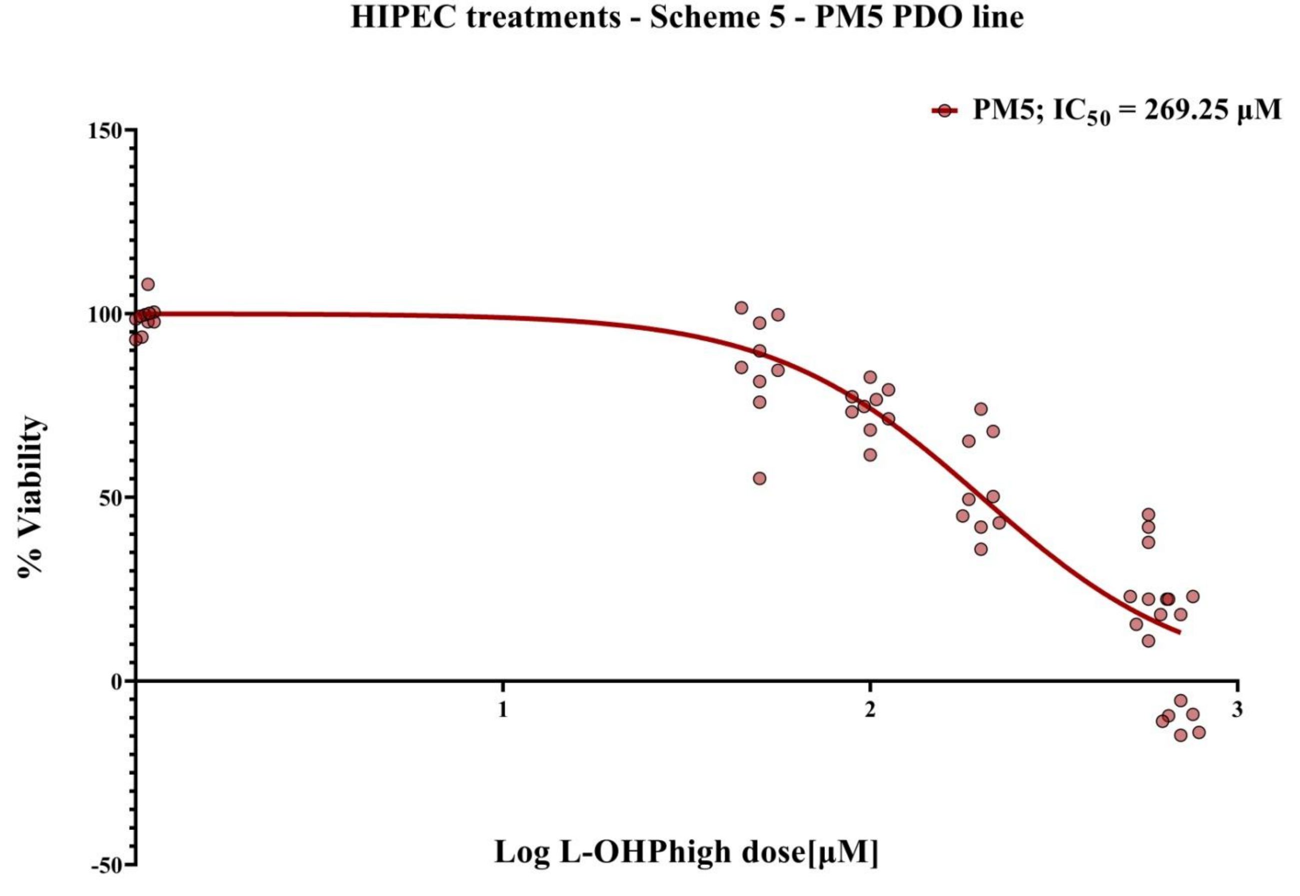

# Supplementary Figure S3

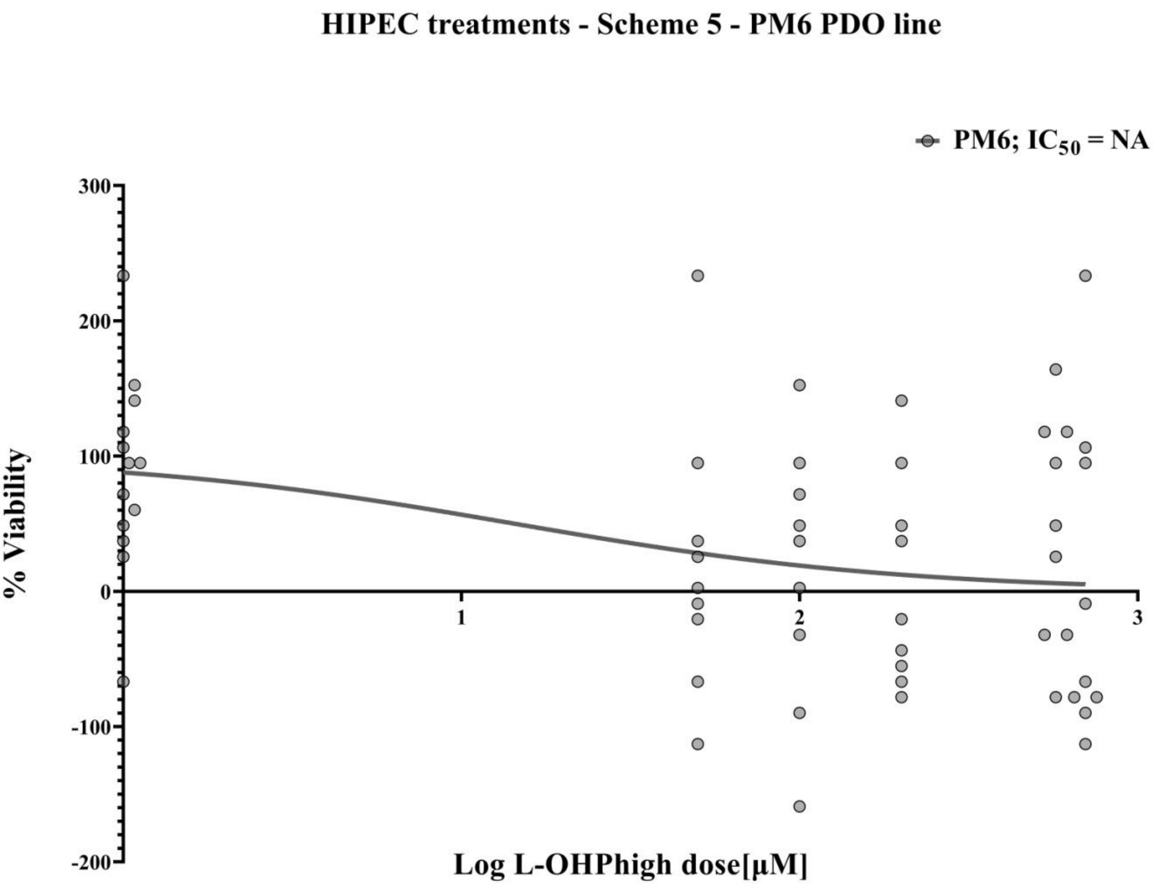

# Supplementary Figure S4

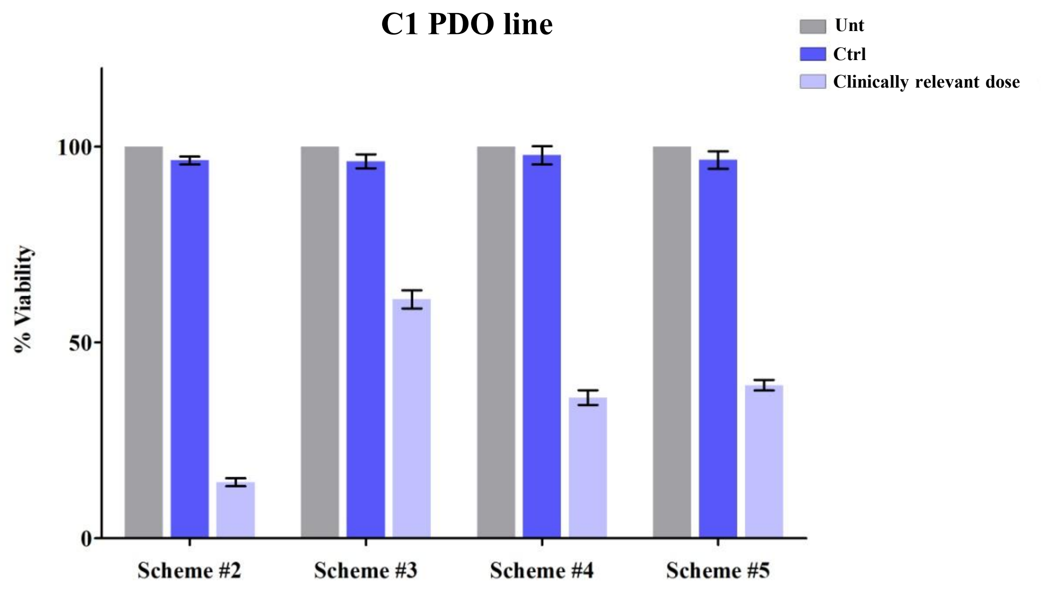


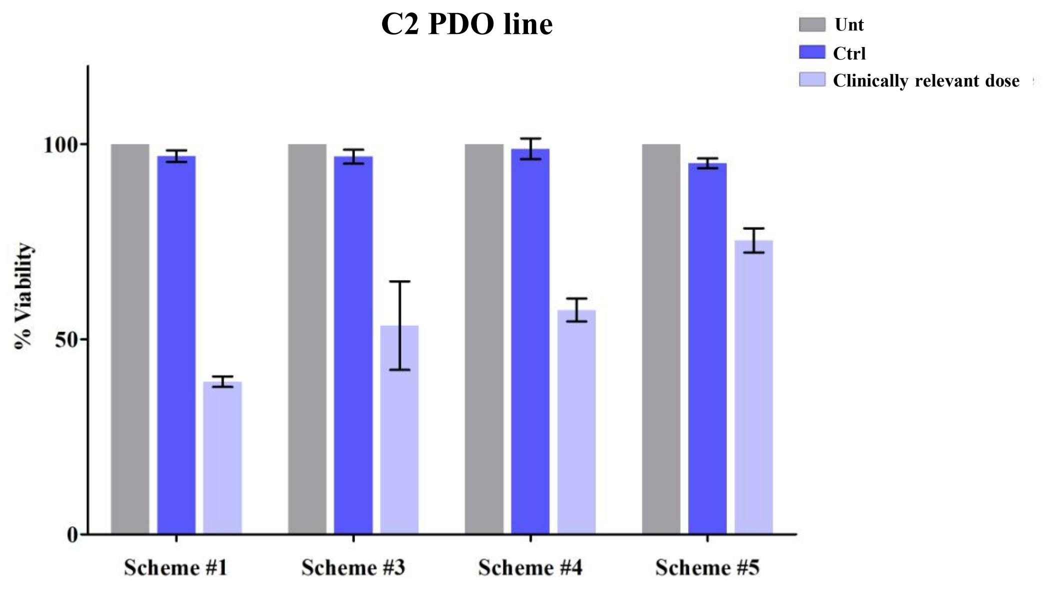


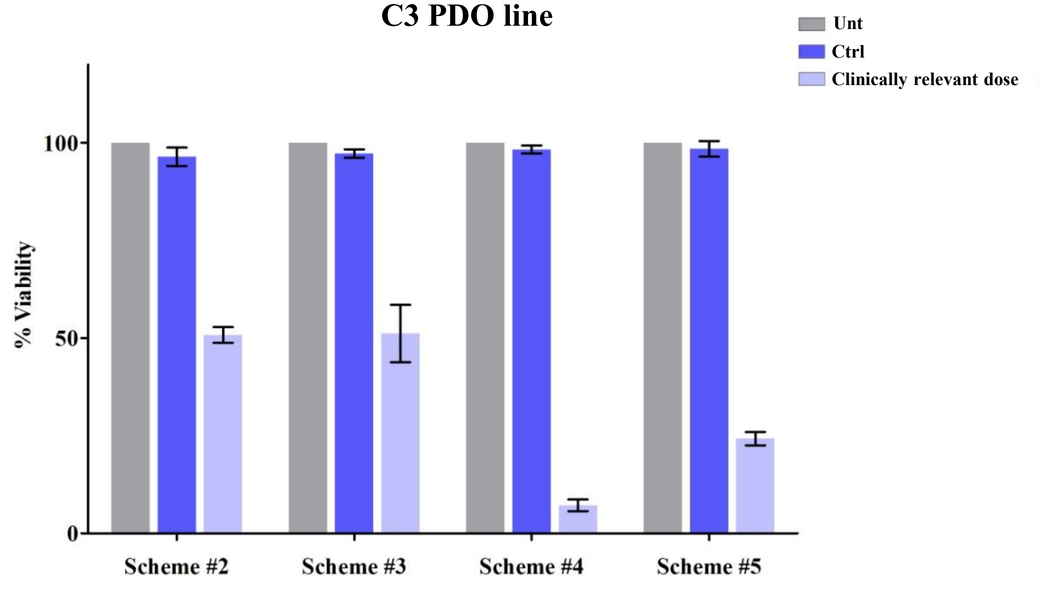

# Supplementary Figure S4

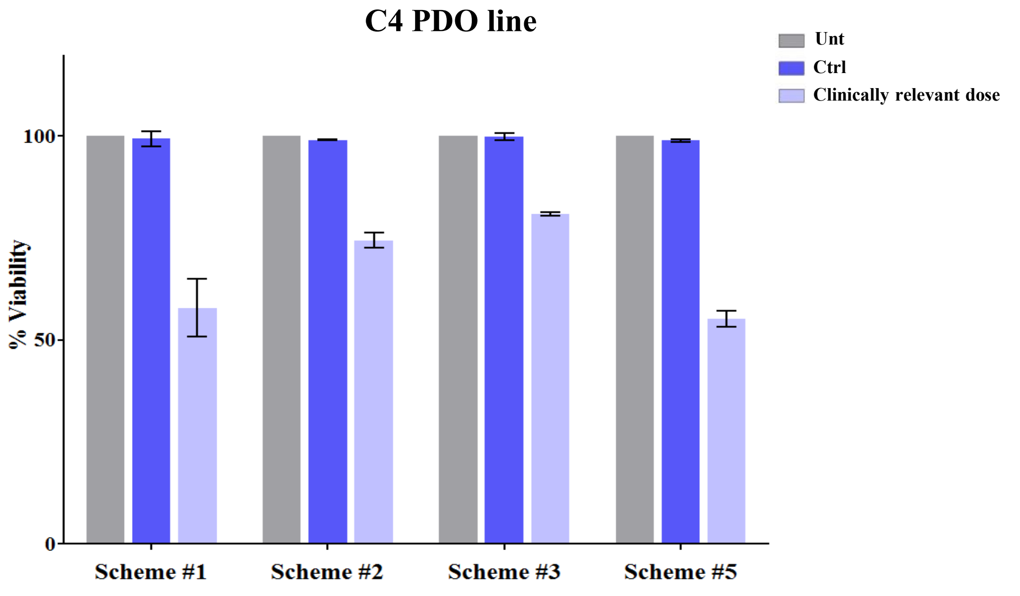


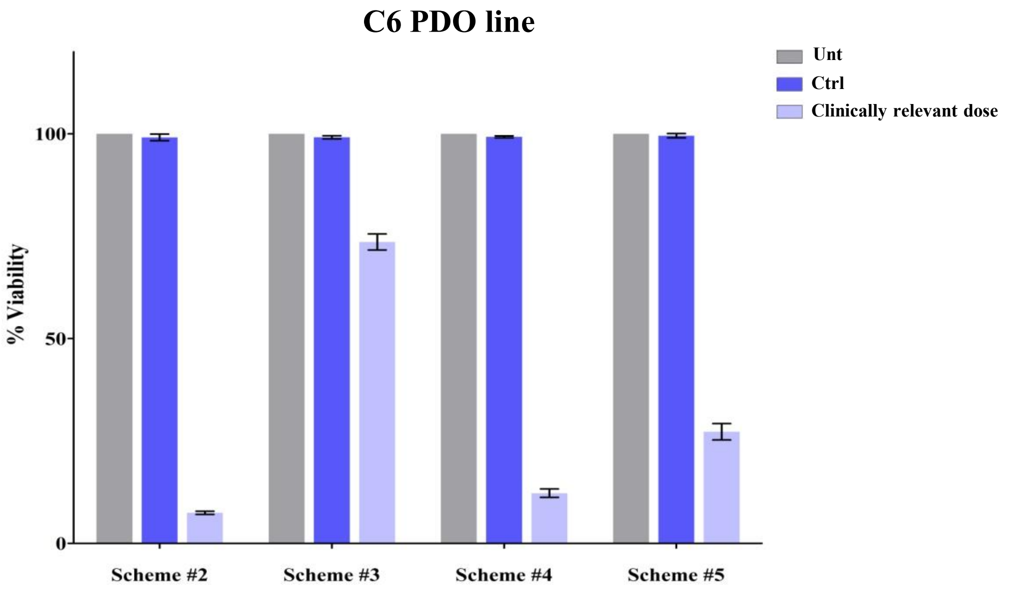


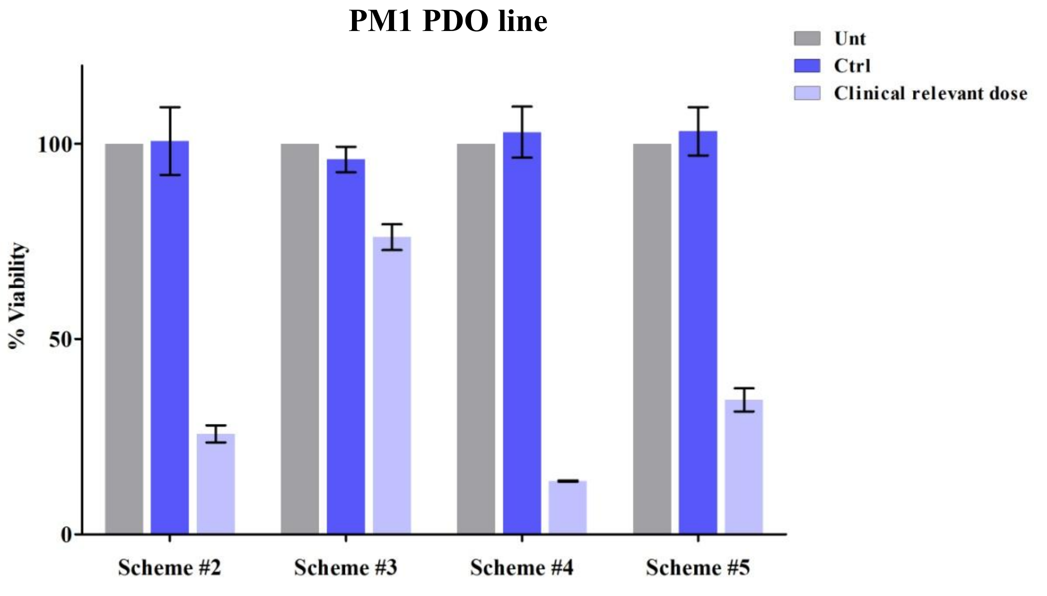

# Supplementary Figure S4

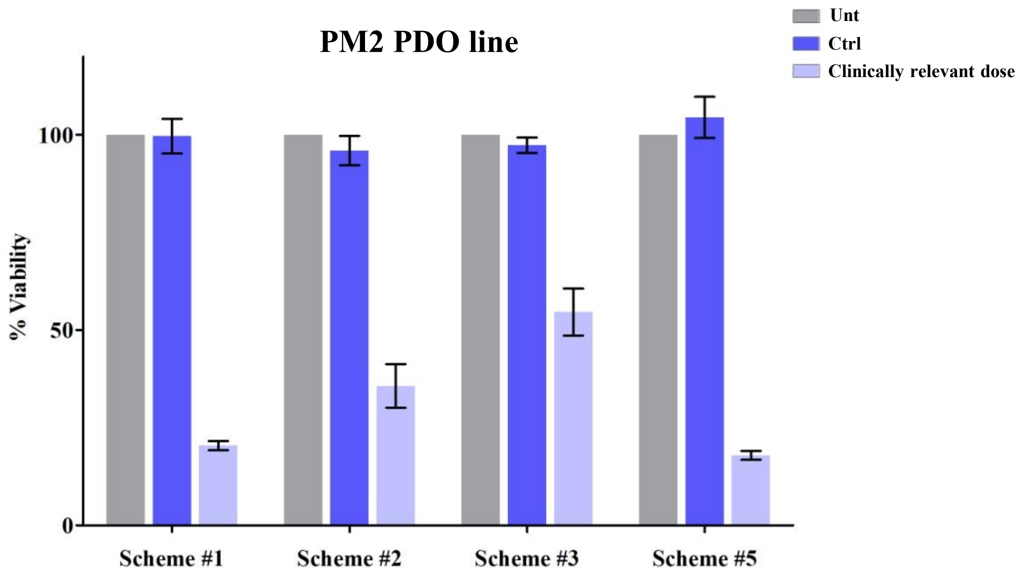


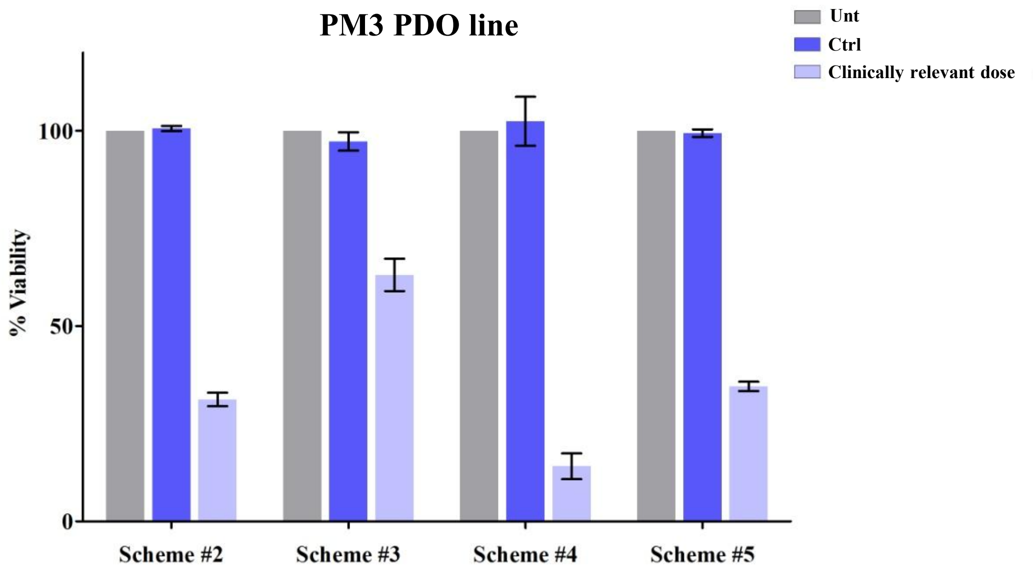


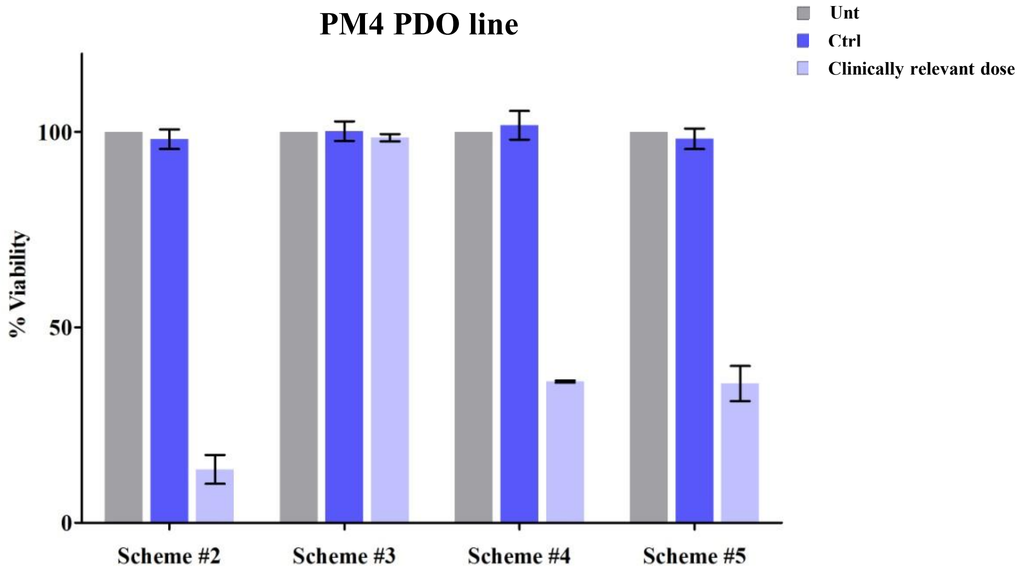

# Supplementary Figure S4

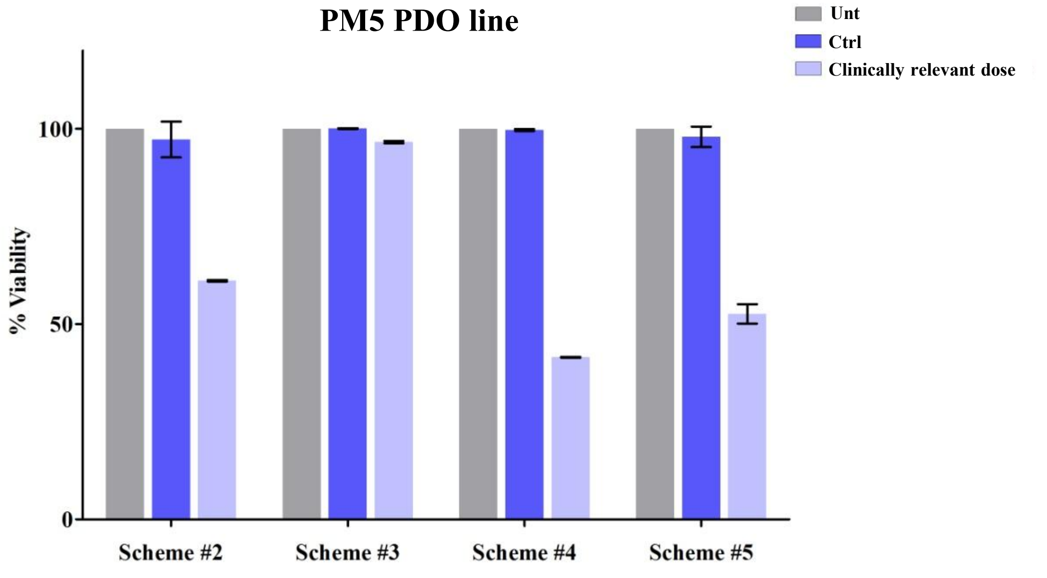


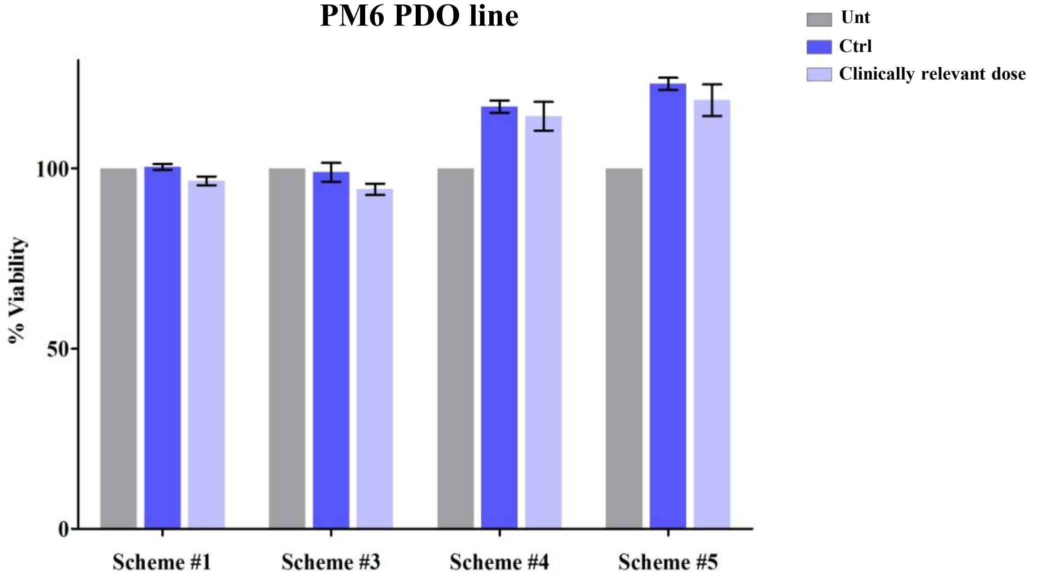


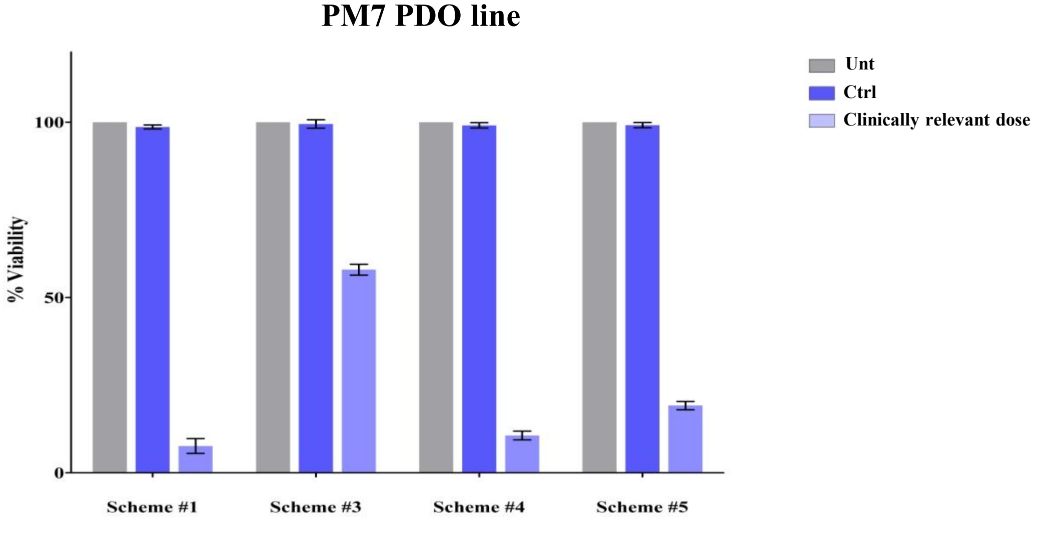

# Supplementary Figure S5

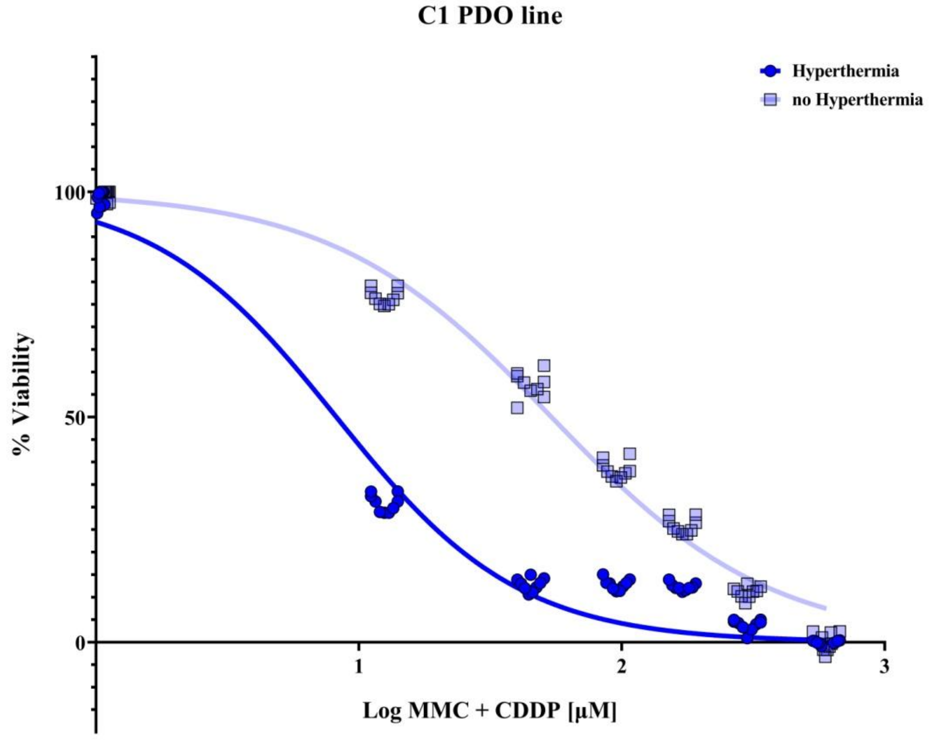


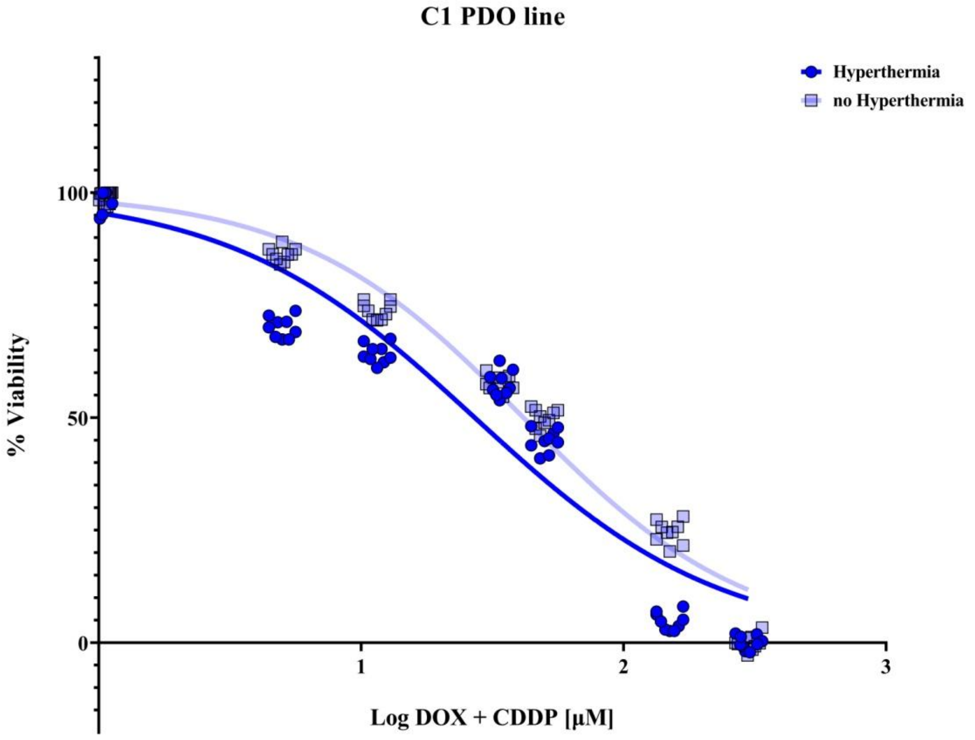

# Supplementary Figure S5

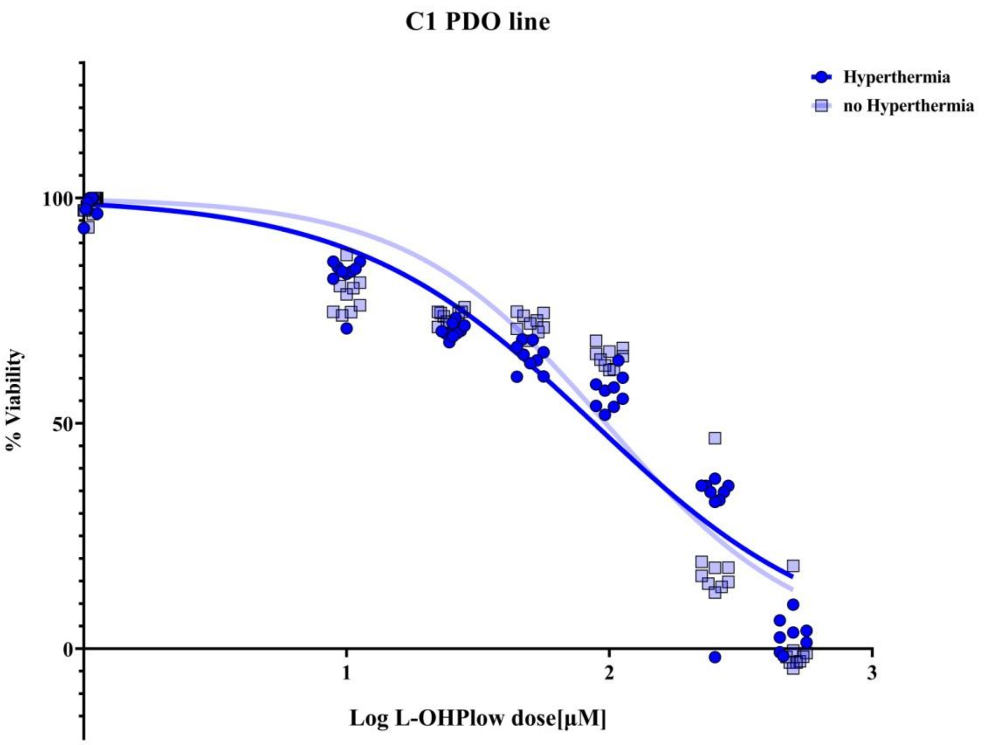


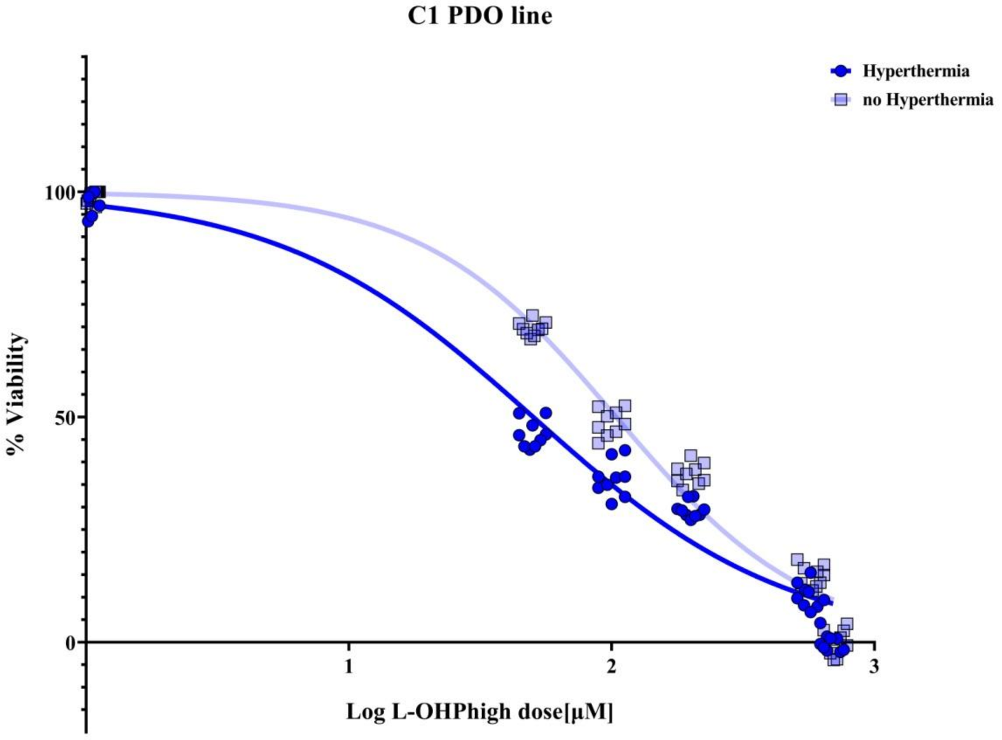

# Supplementary Figure S4

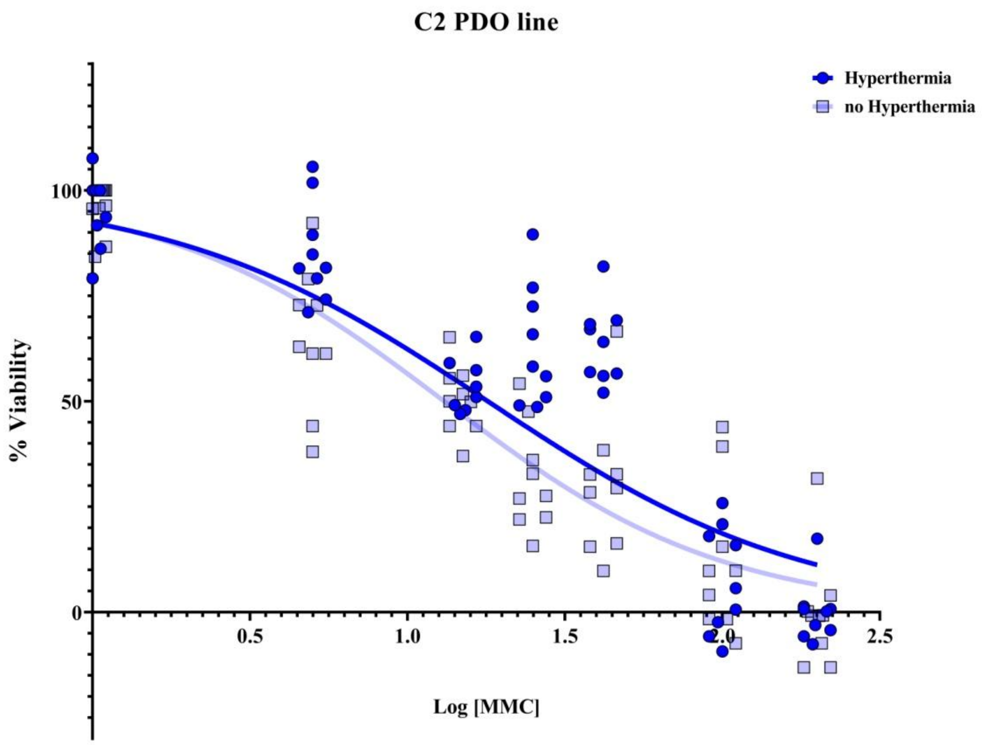


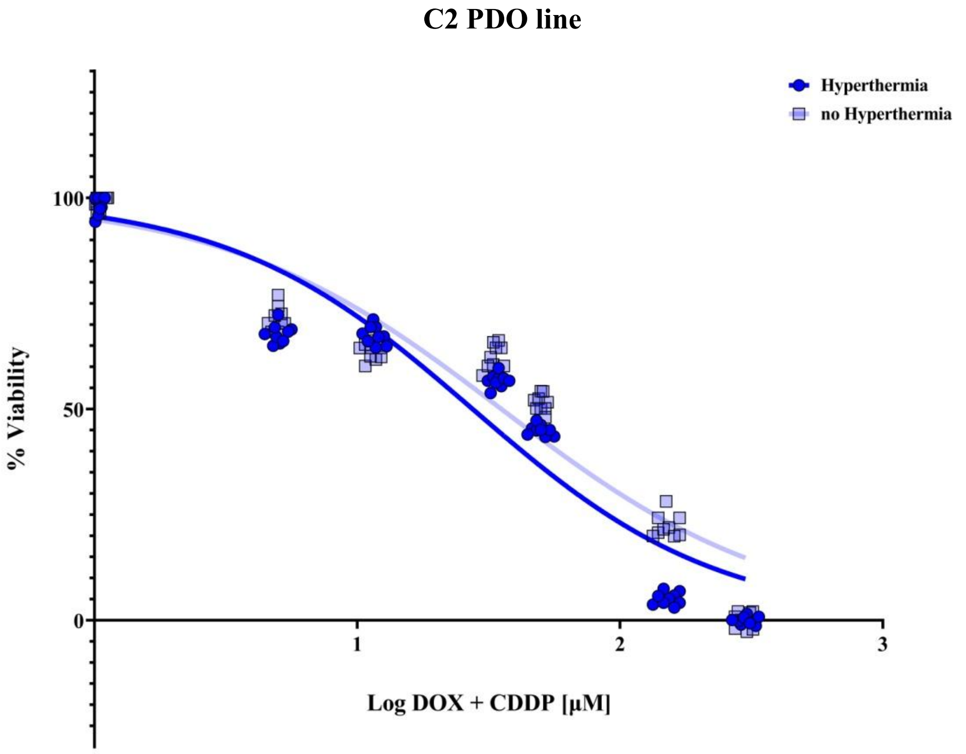

# Supplementary Figure S5

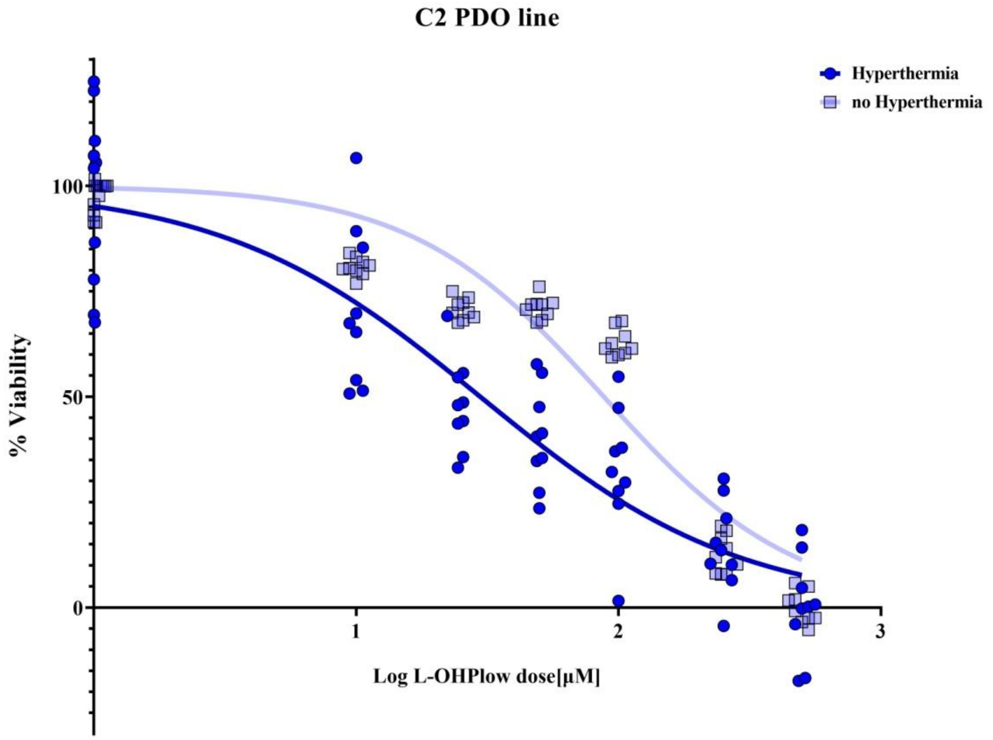


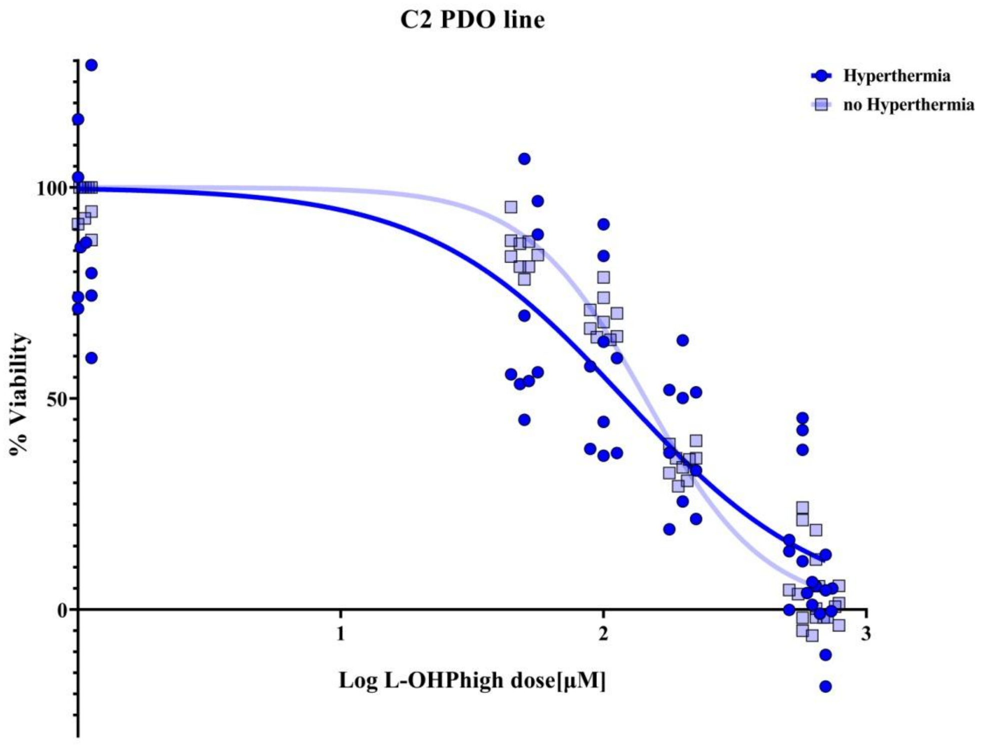

# Supplementary Figure S5

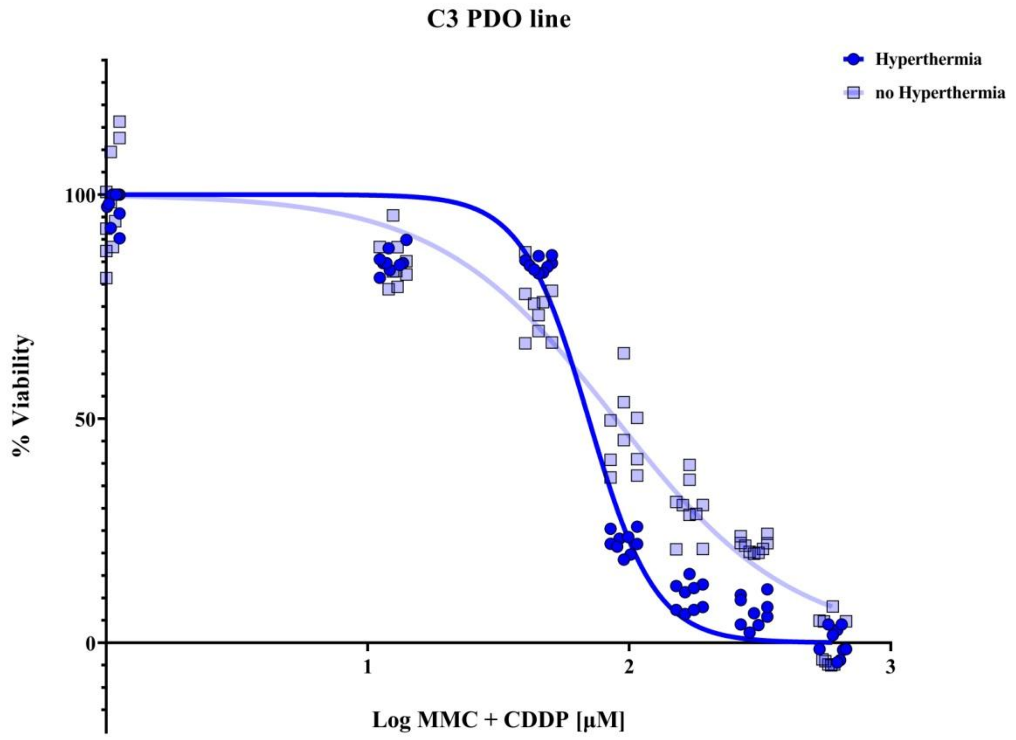


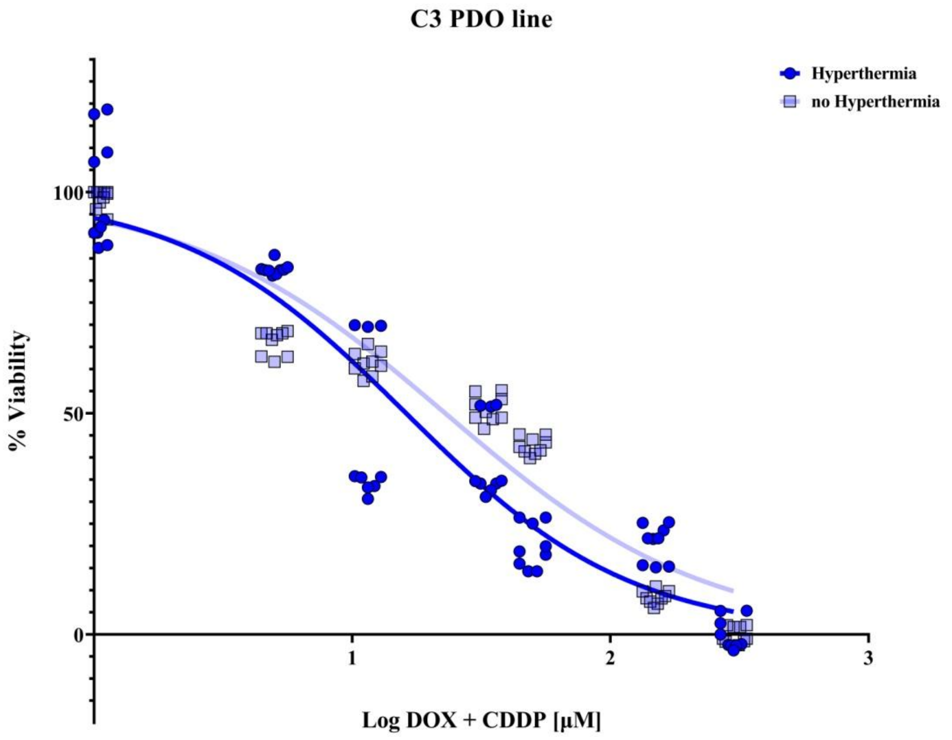

## Supplementary Figure S5

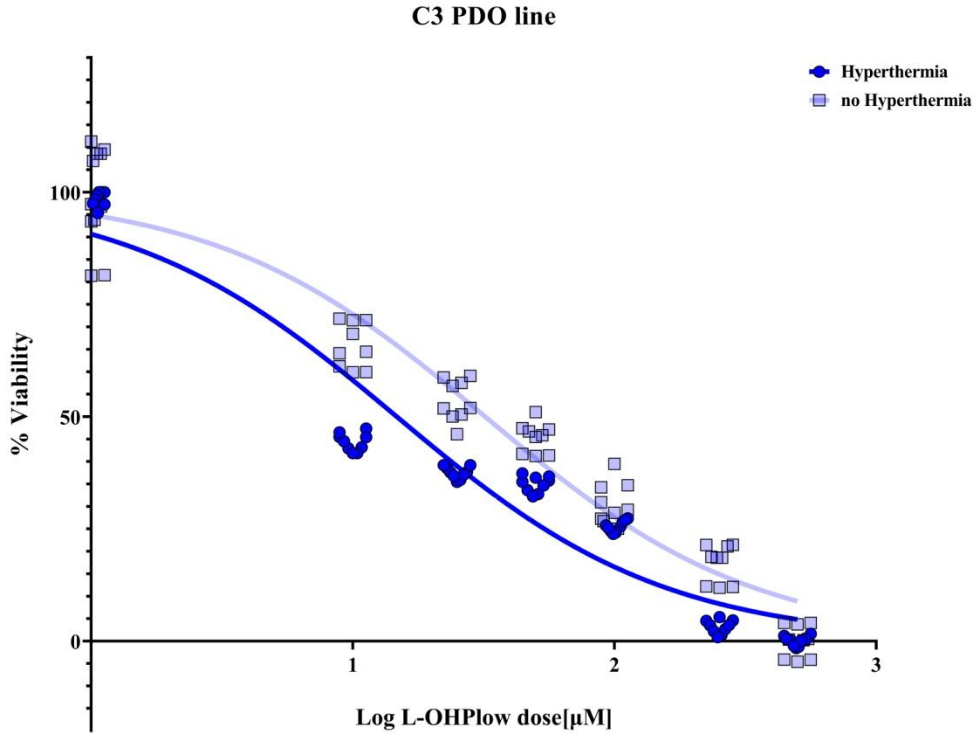


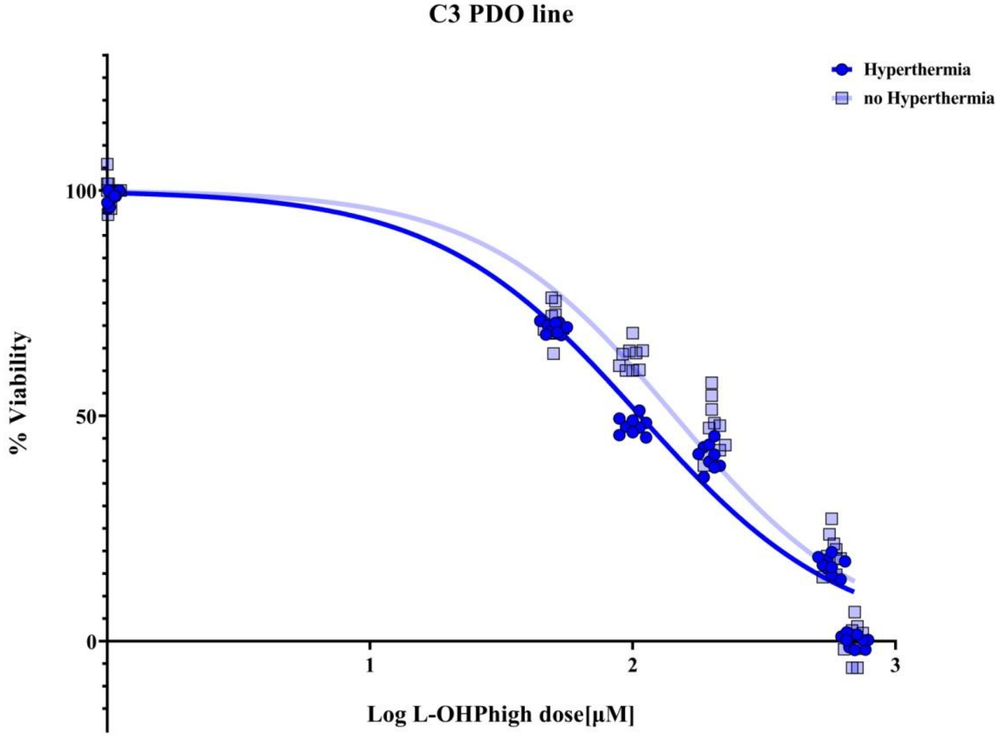

**Supplementary Figure S6**

# WB#1 CAF CRCPM

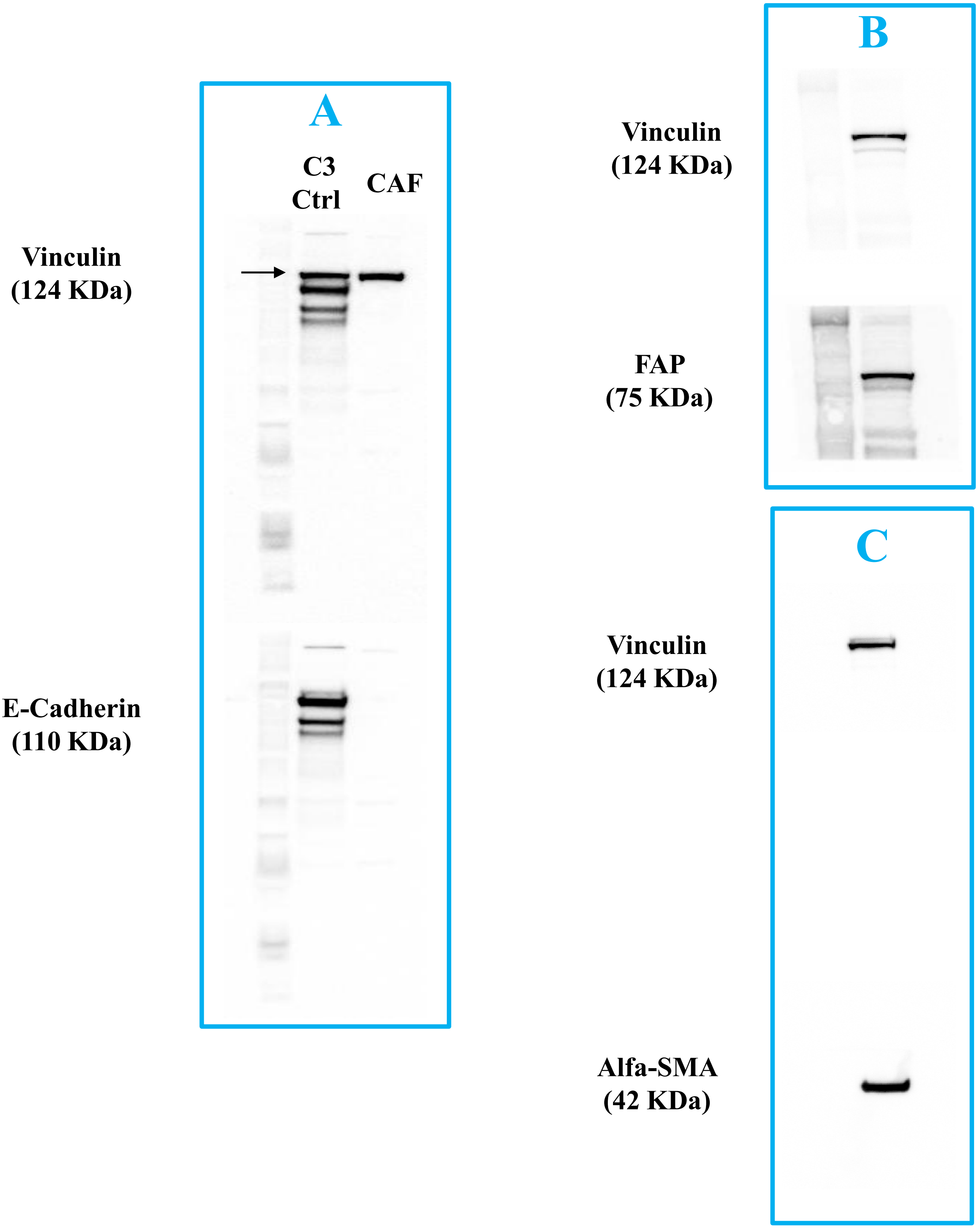

**Supplementary figure legends**

**Supplementary Figure S1 legend:** ***Characterization of CRCPM-derived PDOs.*** (**A**) Schematic representation of the protocol used to develop CRCPM-PDOs. Cells isolated from the tumor were cultured in presence of different growth factors, to mimic different niche factors conditions (WRENAS-based media). WRENAS: **W:** Wnt family member 3A; **R:** R-spondin 1 protein; **E:** Epidermal Grow Factor; **N:** Noggin protein; **A:** A83-01 – anti-p38 inhibitor; **S:** SB 202190 – anti-ROCK inhibitor

**Supplementary Figure S2 legend: PDO medium selection:** Bar plot indicating the best medium condition for each CRCPM-derived PDO cultures (C1, C2, C3, C4, C6, PM1, PM2, PM3, PM4, PM5, PM6 and PM7). The best medium formulation was determined by directly counting the number of PDO presented in each well. The experiments were performed at least in duplicate, counting three different fields per well. Differences among different media conditions were evaluated by *Student's t-test* (** $p < 0.01$; *** $p < 0.001$).

**Supplementary Figure S3 legend:** ***PDO response to HIPEC simulation.*** Dose-response curves of CRCPM-derived PDO illustrating the variation in sensitivity to MMC (scheme 1; PDO lines: C2 and PM6), MMC + CDDP (scheme 2; PDO lines: PM4 and PM6), DOX + CDDP (scheme 3; PDO lines: PM4, PM5 and PM6), L-OHP$_{low\text{-}dose}$ (scheme 4; PDO lines: PM6) and L-OHP$_{high\text{-}dose}$. (scheme 5; PDO lines: PM4, PM5 and PM6).

**Supplementary Figure S4 legend: PDO model systems treated with different HIPEC schemes.** Percentage of alive C1, C2, C3, C4, C6, PM1, PM2, PM3, PM4, PM5, PM6 and PM7 PDO cells after HIPEC treatments, measured as chemioluminescent signal of the concentration of intracellular ATP. PDO were treated with drug concentration corresponding to the calculated clinical relevant dose. Data are presented as median and SD and the experiments were performed in triplicate. UNT: PDO treated with media only at 42.5 °C; CTRL: PDO treated with 0.1 % of physiological solution at 42.5 °C.

**Supplementary Figure S5 legend:** Dose-response curves of CRCPM-derived PDO (C1, C2 and C3) illustrating the variation in sensitivity to MMC (scheme 1), MMC + CDDP (scheme 2), DOX + CDDP (scheme 3), L-OHP$_{low\text{-}dose}$ (scheme 4) and L-OHP$_{high\text{-}dose}$ (scheme 5) in hyperthermia and non-hyperthermia conditions.

**Supplementary Figure S6 legend: CRCPM-derived CAF characterization:** Immunoblots of (**A**) E-cadherin, (**B**) FAP, (**C**) and α-SMA protein expression levesl in CRCPM-derived CAF lysates. Vinculin was used as loading control. Uncropped images were reported.
